# Prediction of Back-splicing sites for CircRNA formation based on convolutional neural networks

**DOI:** 10.1186/s12864-022-08820-1

**Published:** 2022-08-12

**Authors:** Zhen Shen, Yan Ling Shao, Wei Liu, Qinhu Zhang, Lin Yuan

**Affiliations:** 1 School of Computer and Software, Nanyang Institute of Technology, Changjiang Road 80, Nanyang, 473004 Henan China; 2 Translational Medical Center for Stem Cell Therapy and Institute for Regenerative Medicine, Shanghai East Hospital, Bioinformatics Department, School of Life Sciences and Technology, Tongji University, Siping Road 1239, Shanghai, 200092 China; 3 Institute of Machine Learning and Systems Biology, School of Electronics and Information Engineering, Tongji University, Caoan Road 4800, Shanghai, 201804 China; 4 School of Computer Science and Technology, Qilu University of Technology (Shandong Academy of Sciences), Daxue Road 3501, Jinan, 250353 Shandong China

**Keywords:** CircRNA, Back-splicing sites prediction, Deep learning, Convolutional neural networks, Batch normalization

## Abstract

**Background:**

Circular RNAs (CircRNAs) play critical roles in gene expression regulation and disease development. Understanding the regulation mechanism of CircRNAs formation can help reveal the role of CircRNAs in various biological processes mentioned above. Back-splicing is important for CircRNAs formation. Back-splicing sites prediction helps uncover the mysteries of CircRNAs formation. Several methods were proposed for back-splicing sites prediction or circRNA-realted prediction tasks. Model performance was constrained by poor feature learning and using ability.

**Results:**

In this study, CircCNN was proposed to predict pre-mRNA back-splicing sites. Convolution neural network and batch normalization are the main parts of CircCNN. Experimental results on three datasets show that CircCNN outperforms other baseline models. Moreover, PPM (Position Probability Matrix) features extract by CircCNN were converted as motifs. Further analysis reveals that some of motifs found by CircCNN match known motifs involved in gene expression regulation, the distribution of motif and special short sequence is important for pre-mRNA back-splicing.

**Conclusions:**

In general, the findings in this study provide a new direction for exploring CircRNA-related gene expression regulatory mechanism and identifying potential targets for complex malignant diseases. The datasets and source code of this study are freely available at: https://github.com/szhh521/CircCNN.

**Supplementary Information:**

The online version contains supplementary material available at 10.1186/s12864-022-08820-1.

## Background

Circular RNAs (CircRNAs) are special non-coding RNA molecules formed by precursor mRNA back-splicing [1, 2]. These RNAs have single-stranded, covalent, and closed-loop structures [1]. With the help of this special structures, CircRNAs can resist degradation from RNA exonuclease, and the expression level of CircRNAs in the cell is more stable [3]. CircRNAs play a critical role in various cellular processes in many ways [4], such as miRNA sponge [5], RNA-protein binding [6] and gene transcription regulation [7]. For example, the interaction between CircRNA LPAR3 and microRNA-198 can facilitate esophageal cancer migration, invasion, and metastasis [8]. In addition to important genes for neurogenesis, Szabo et al. found that the expression level of CircRNA and its isoform is higher in the developing brain [9]. circSKA3 induces invadopodium formation by binding integrin β1, thus enhancing breast cancer invasion ability [10]. The interaction between EIciRNA–U1 snRNP complexes and Pol II transcription complex at the promoters of parental genes can enhance gene expression [11]. Most CircRNAs are non-coding RNA. However, a recent study found that some CircRNAs have translation ability like mRNA [12, 13]. In addition, researchers have found traces of CircRNAs in various complex malignant diseases [14, 15].

During CircRNA formation, narrowing the distance between a downstream splice-donor (SD) and an upstream splice-acceptor (SA) is a key step [1]. One way is with cis-element, SA flanking intron region and SD flanking intron region contain a ciselement (also be known as inverted repeat element, like Alu element), base pairing between cis-elements bring upstream SA and downstream SD closer, and then promote CircRNA formation [4]. On the other hand, some RBP (RNA Binding Protein) can play a role in linear RNA splicing and regulate pre-mRNA back-splicing. These RBPs first bind with motif sites in the flanking intron of SA and SD, RBPs dimerization can also bring upstream SA into proximity with downstream SD [1, 4]. During murine embryonic stem cell-derived motor neuron formation, RBP FUS regulates CircRNA expression by binding the intronic FUS-binding motifs flanking the back-splicing junctions [16]. In summary, circRNA formation is a very complicated biological process, special function sites in the flanking exons or introns are important for CircRNA formation. However, there is still a lack of details about the back-splicing regulation mechanism and the special function sites in the flanking introns and exons.

Identifying the sequence specificities of back-splicing sites is considered as a binary classification task, and some algorithms have been proposed. These algorithms use sequence features as input data and can find important features for identifying the types of splicing events (linear splicing or back-splicing). In 2017, SVM (Support Vector Machine) and RF (Random Forest) were introduced as classifiers for predicting pre-mRNA back-splicing sites [17]. Since short sequence features lack biological explanation, the application of these models was limited.

Deep learning not only solves the shortcomings of machine learning algorithms but also explores potential laws from huge amounts of raw data and has achieved excellent performance in computer vision (CV) [18], natural language processing (NLP) [19], speech recognition [20] and other fields. DeepBind was the first deep learning model for DNA/RNA motif site analysis [21]. Since then, many deep learning-based models had been proposed for genome sequence analysis [22, 23], like MSCGRU [24], iDeepS [25], DeepCirCode [26], PASSION [27], circDeep [28]. CNN (Convolutional Neural Network) and RNN (Recurrent Neural Network) are the basic elements that make up these models. A convolution kernel can extract potential functional subsequence features, combining the output and weights of the convolution layer can get visual motif site information. RNN can learn the mutual regulation features between different motif sites.

In this study, we proposed CircCNN to explore the special function sites of back-splicing. For each circRNA, the SA and SD related to circRNA formation are unique. To avoid mutual interference, CircCNN uses a convolution layer to extract the specific features of SA sites and SD sites respectively. Experimental results showed that CircCNN outperforms other baseline models. Compared with existing models, CircCNN can not only predict whether the input sequence contains back-splicing sites but also give the visual motif sites information. The research found that some human motifs are conserved in mouse and fruit fly [29, 30], motifs obtained by CircCNN from three datasets (human, mouse, and fruit fly) also proved this conclusion.

## Results

### Experimental settings

In this study, the parameters of CNN used to extract features from input data are shown in Table 1. RMSProp is used to optimize model training. In addition to dropout, Early stopping is also used to avoid overfitting. The number of early stopping rounds is 20. About epochs, because of using early stopping in our model, we only set the max epoch to 100. The batch size is set to 1024. Unlike the cross-validation strategy used in DeepCirCode, in CircCNN, all datasets may be involved in model training or testing with 7-fold cross-validation (see Supplementary section “k-fold Cross-Validation”). We can obtain the parameter combination that meets our requirements by comparing model performance with different parameter combinations (See Supplementary Table S1). The bold and italic numbers in Table 1 represent the optimal parameters for our model.

**Table 1:** Model parameter

Layer	Parameter
Conv1	Kernel number: 256, Kernel size: 10,***12***,15 Padding mode: Valid, Stride window: 1
Conv2	Kernel number: 128, Kernel size: 20,***30***,40 Padding mode: Same, Stride window: 1,***2***
Drop1	0.2,0.5,***0.7***
MP	(5,5)
Drop2	0.2,0.5,***0.7***

Conv represents Convolution layer, Drop represents dropout layer, MP represents Max pooling layer

Here, not only were the existing models introduced as comparison models, but we also compared CircCNN with the different combinations of encoding methods (one-hot or word2vec), CNN, LSTM (Long Short-Term Memory), and attention. For the model using word2vec, its input data was divided into kmer sequences (6-mer). All comparison models are shown as follows: ①Onehot+CNN + LSTM,②Onehot+CNN + Attention, ③Onehot+CNN + LSTM+Attention,④Word2vec + LSTM, ⑤Word2vec + LSTM+Attention, ⑥ DeepCirCode, ⑦CircCNN (CVLD),⑧CircCNN.

It should be noted that the three datasets we used in this study are imbalanced. When choosing model evaluation metrics, we must consider the situation of data imbalance. Based on the statistics of model prediction results provided by the confusion matrix (see Supplementary section “Confusion Matrix”), various metrics can be calculated: ACC, AUC, Precision, Recall, and so on. Here, five metrics were used to evaluate model performance: ACC (Accuracy), Sensitivity (also known as True Positive Rate, TPR), Specificity, MCC (Matthews correlation coefficient), and AUC (Area under the ROC curve).

### Experimental results and analysis

**Fig. 1:**
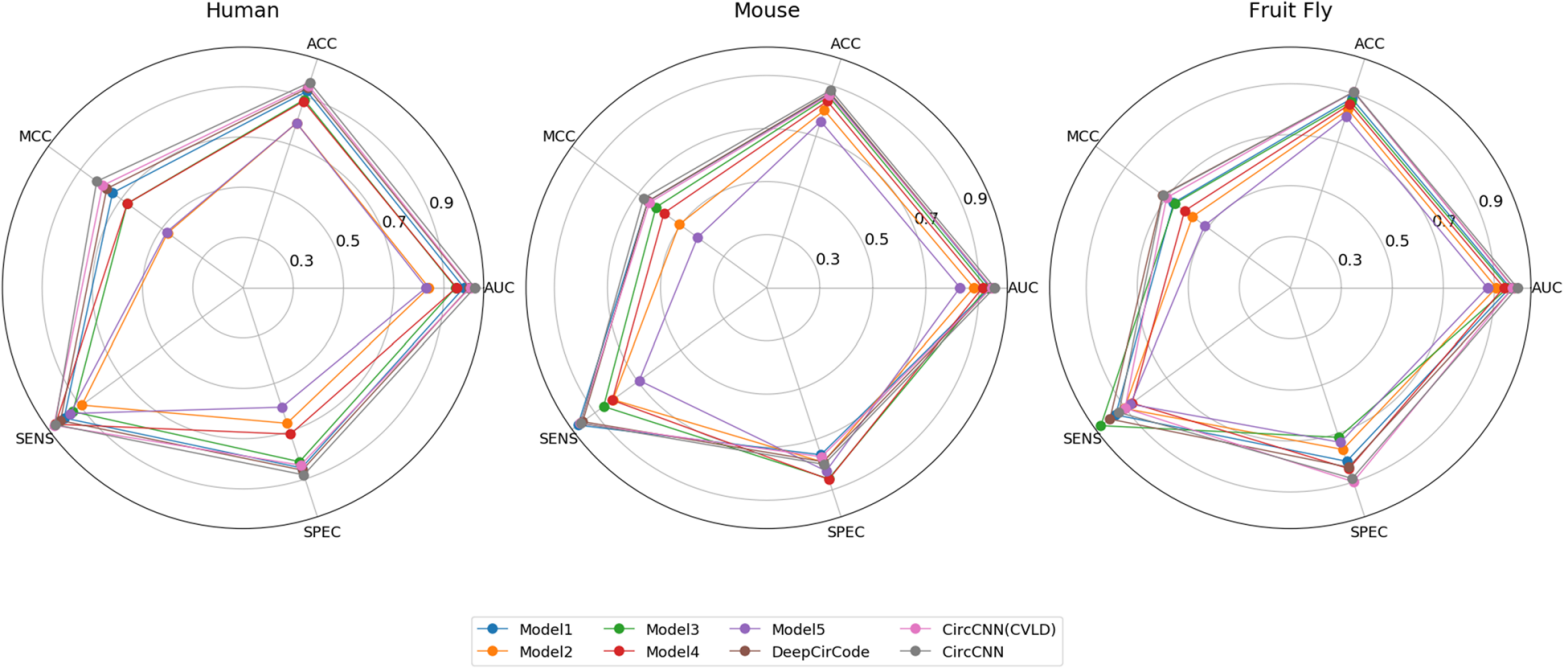
Best performance of circCNN and other baseline models

In this study, CircCNN and all comparison models are performed on three datasets described in the “Data” section. The average value of AUC and ACC of these models are shown in Table 2, and the other three metrics of these models are shown in Supplementary Table S2. Figure 1 shows the best performance of CircCNN and other baseline models in 7-fold cross-validation. As can be seen, CircCNN outperforms other baseline models. CircRNA is the product of pre-mRNA alternative back-splicing, which means that the association between different functional sites in the pre-mRNA sequence is weakened. The functional sites in the CircRNA sequence are treated as a word in the text. Word2vec is not good at learning word features from disrupted text to generate word vectors. Therefore, the model using word2vec is not better than the one-hot method. Drawing on existing embedding methods, we can study a new embedding method suitable for CircRNA to improve model performance.

**Table 2:** Comparison of CircCNN and other baseline models in cross-validation

Model	Human		Mouse		Fruit Fly	
	AUC	ACC	AUC	ACC	AUC	ACC
Model①	0.8614	0.8019	0.8347	0.7669	0.8518	0.7755
Model②	0.7245	0.6744	0.7715	0.7054	0.7716	0.703
Model③	0.8393	0.7793	0.82	0.7525	0.8415	0.7593
Model④	0.8334	0.762	0.7915	0.7188	0.8231	0.7398
Model⑤	0.7117	0.6647	0.7242	0.6637	0.743	0.676
DeepCircCode	0.8827	0.8232	0.8391	0.7653	0.8611	0.7796
CircCNN (CVLD)	0.9026	0.8348	0.8431	0.7572	0.8704	0.7807
CircCNN	**0.9049**	**0.8421**	**0.8514**	**0.7705**	**0.8708**	**0.7869**

CVLD represents the cross-validation strategy used in CircCNN training is same as DeepCirCode

### Explore the influence of batch normalization and dropout on model performance

Since its launch in 2015, batch normalization is widely used in CV, NLP, and other fields. To explore the influence of batch normalization (BN) on model performance, we perform CircCNN without BN (No BN) or use dropout to replace the batch-normalization (BN → Dropout) in CircCNN, and then compare it with the original CircCNN.

We found that model training time did not change significantly during the experiment. As can be seen from Table 3, the performance difference between CircCNN (No BN) and CircCNN (BN → Dropout) is not obvious. In addition to Specificity, CircCNN outperforms other models on four metrics. Generally, dropout can only be used to deal with over-fitting problems during model training, while BN can reduce the impact of over-fitting and vanishing gradients on model performance. Whether BN is used and where BN is located in the model still needs to be determined by experiments.

**Table 3:** CircCNN with BN outperforms other modified CircCNN

		AUC	ACC	MCC	Sens	Spec
Human	CircCNN (No BN)	0.8963	0.8317	0.6613	0.8871	**0.766**
	CircCNN (BN → Dropout)	0.8979	0.8325	0.6632	0.8891	0.7655
	CircCNN	**0.9049**	**0.8421**	**0.6849**	**0.9147**	0.7562
Mouse	CircCNN (No BN)	0.8401	0.7629	0.5275	0.8003	**0.7255**
	CircCNN (BN → Dropout)	0.8407	0.7623	0.5263	0.8001	0.7246
	CircCNN	**0.8514**	**0.7705**	**0.5508**	**0.8614**	0.6797
Fruit Fly	CircCNN (No BN)	0.858	0.773	0.5483	0.8058	**0.7402**
	CircCNN (BN → Dropout)	0.86	0.7753	0.5527	0.8165	0.7341
	CircCNN	**0.8708**	**0.7869**	**0.5773**	**0.8374**	0.7365

Each number represents the average metric value of model in cross-validation

### Motif analysis

#### Motif found by CircCNN match the known motif in motif database

Accurate identification of back-splicing sites helps to explore the CircRNA-related regulation mechanism. In this study, we first convert the output of the first convolution layer to PPMs (Position Probability Matrix), and then use TOMTOM [31] to compare it with the known motifs. The whole process can be described as the following three steps.

1. Input data→CMEM: The output of the first convolution layer can be called CMEM (Candidate Motif Evaluation Matrix) set. Each element in CMEM is a probability given by a convolution kernel, which represents whether the subsequence is a motif or not.
2. CMEM→PPM: Based on the evaluation score in CMEM, all positive samples provided a lot of subsequences. Each PPM corresponds to a CMEM. To calculate a PPM, the first thing to do is to count the number of each nucleotide at a position, and then divide the count result by the total number of subsequences.
3. PPM → motif logo: In this step, PPM was uploaded to TOMTOM for motif comparison and motif logo generation.

**Fig. 2:**
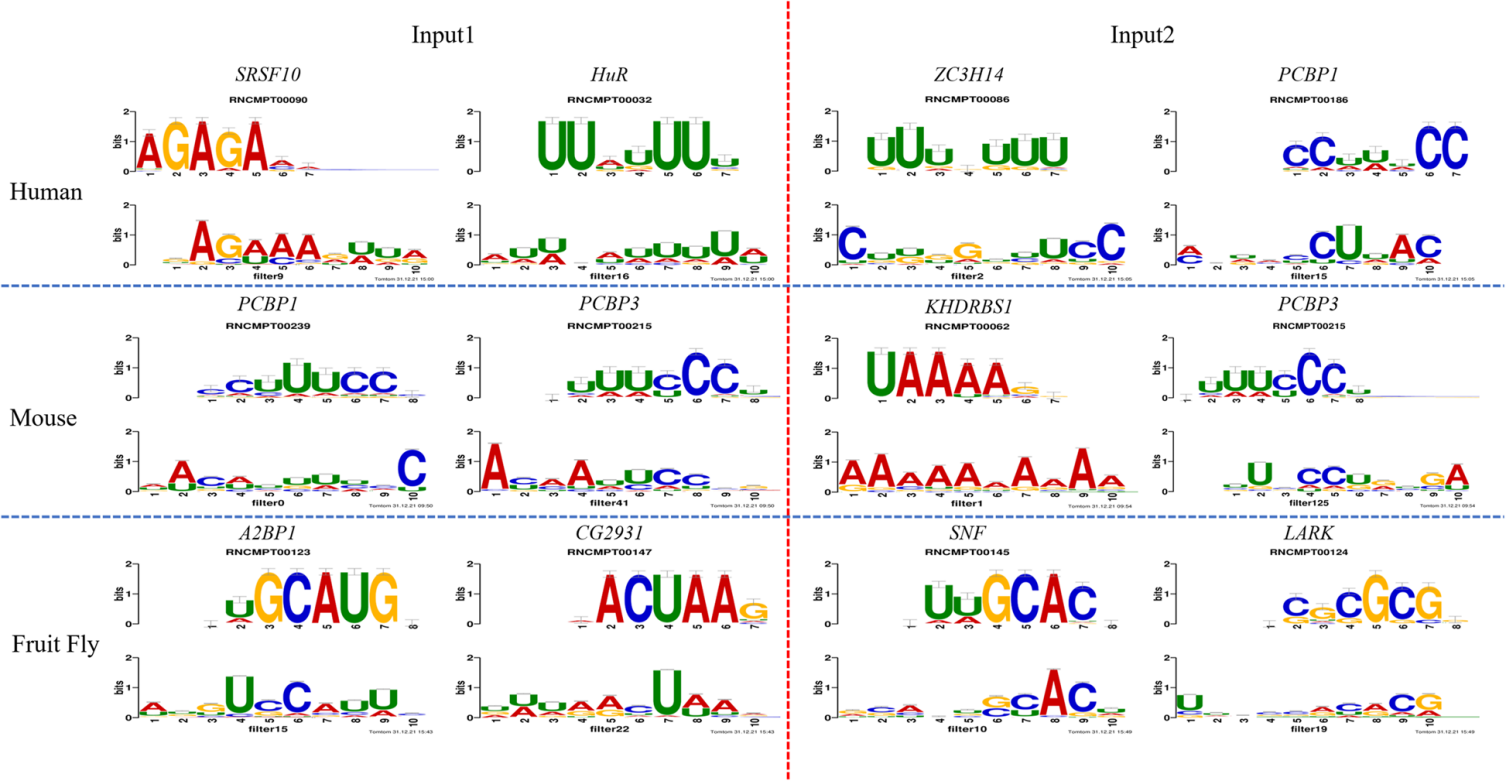
Sequence logos of three species matched motifs. From top to bottom, motif logos of three species are shown respectively, both sides of the red line are the motifs of input1 module and input2 module, respectively. The gene name is shown above each motif logos

Three known motif databases obtained from MEME Suite [32] were used in motif analysis: RNA/Ray2013_rbp_Homo_sapiens.meme, RNA/Ray2013_rbp_Mus_musculus.meme, RNA/Ray2013_rbp_Drosophila_melanogaster.meme. It should be clear that motifs in this section and the next section are found by CircCNN, which has the best performance in 7-fold cross-validation. Part of the motif found by CircCNN is shown in Table 4 and Fig. 2, and the rest is shown in Supplementary Table S3, S4, S5, S6, S7, and S8.

**Table 4:** Three species motifs found by CircCNN match three known motif databases by TOMTOM

		FilterID	Motif found by CircCNN	Known motif in database	Known motif sequence	Gene Annotation	E-value
Human	Input1	**filter9**	**GAGAAAGUUA**	**RNCMPT00090**	**AGAGAAA**	**SRSF10**	**0.0638**
		**filter16**	**AUUUAUUUUA**	**RNCMPT00032**	**UUAUUUU**	**HuR**	**0.0115**
		filter36	UCUCUUUUUG	RNCMPT00012	CUUUUUU	CPEB2	0.0205
	Input2	**filter2**	**CUUGGUUUCC**	**RNCMPT00086**	**UUUGUUU**	**ZC3H14**	**0.0766**
		**filter15**	**AGUACCUUAC**	**RNCMPT00186**	**CCUUUCC**	**PCBP1**	**0.0418**
		filter18	CCAUUUUCUU	RNCMPT00269	ACUUUCU	PTBP1	0.0133
Mouse	Input1	filter0	**AACAUUUUCC**	**RNCMPT00239**	**CCUUUCCC**	**PCBP1**	**0.0072**
		filter41	ACAAUUCCCG	RNCMPT00239	CCUUUCCC	PCBP1	0.0498
			**ACAAUUCCCG**	**RNCMPT00215**	**CUUUCCCU**	**PCBP3**	**0.0956**
	Input2	**filter1**	**AAAAAAAAAA**	**RNCMPT00062**	**UAAAAGG**	**KHDRBS1**	**0.0189**
		**filter125**	**UUCCCUGUGA**	**RNCMPT00215**	**CUUUCCCU**	**PCBP3**	**0.0452**
		filter160	UGUAUGAGGA	RNCMPT00051	GUGUGUG	RBM38	0.0673
			UGUAUGAGGA	RNCMPT00062	UAAAAGG	KHDRBS1	0.0972
Fly	Input1	**filter15**	**AUGUCCAUUC**	**RNCMPT00123**	**GUGCAUGC**	**A2BP1**	**0.0499**
		**filter22**	**UUUAACUAAA**	**RNCMPT00147**	**AACUAAG**	**CG2931**	**0.023**
		filter32	GUUGGGUUUA	RNCMPT00120	UUUAGUU	FNE	0.0536
	Input2	**filter10**	**GCACUGCACU**	**RNCMPT00145**	**AUUGCACA**	**SNF**	**0.0465**
		**filter19**	**UUACCACACG**	**RNCMPT00124**	**CCGCGCGG**	**LARK**	**0.0375**
		filter46	UAAUAAACUU	RNCMPT00142	AUAAUAA	QKR58E-1	0.0377

According to the records in UniProt, the proteins encoded by genes interacting with known motifs in Table 4 and the supplementary table are involved in gene expression regulation. For example, HuR/TIA1 is involved in alternative splicing regulation of SIRT1 Pre-mRNA by promoting or inhibiting exon8 exclusion [33]. TIA1, which functions as an ARE-binding regulatory factor, is involved in cyclooxygenase-2 translational regulation by binding the AU-rich element (ARE) within the COX-2 mRNA 3′-untranslated region (3’UTR) [34].

By searching the literature and querying disease-related databases, we confirmed that some motifs found by CircCNN are associated with disease-related genes. Table 5 shows some of the associations we found. In lung cancer, RNA binding Protein QKI, whose binding motif RNCMPT00047 is matched with filter209, regulates the alternative splicing of NUMB by binding to two RNA elements in the pre-mRNA of NUMB, thereby inhibiting the proliferation and transformation of lung cancer cells [35]. HNRNPC is associated with cell proliferation and tumor growth in breast cancer, and its binding motif RNCMPT00025 matched with filter188 [36]. In Pancreatic ductal adenocarcinoma cells, the regulation of PTBP1 on pyruvate kinase gene alternative splicing affects the therapeutic effect of gemcitabine [37]. RNA binding protein TIA1 can be targeted by mir-19a, thus affecting cell proliferation and migration in colorectal cancer cells [38]. For HNRNPK, filter162 matches with its motif RNCMPT00026, it can affect the expression of splicing regulator SRSF1, thereby indirectly regulating CD44E alternative splicing, which in turn affects cancer cell proliferation, migration, and invasion [39]. Taken together, CircCNN discovers protein binding sites associated with cancer.

**Table 5:** Association between motif, gene and disease

FilterID	Motif found by CircCNN	Known motif in database	Known motif sequence	Gene Annotation	Disease
filter188	UAUCUUUUUA	RNCMPT00025	AUUUUUU	HNRNPC	Breast Cancer
filter16	AUUUAUUUUA	RNCMPT00032	UUAUUUU	HUR	Gastric Cancer
filter169	UAGACACACA	RNCMPT00027	ACACACA	HNRNPL	Prostate Cancer
filter209	AACAAACAGG	RNCMPT00047	ACUAACA	QKI	Lung Cancer
filter28	UUUUUUCCGA	RNCMPT00165	UUUUUUC	TIA1	Colorectal Cancer
filter162	GACCCAUCCA	RNCMPT00026	CCAACCC	HNRNPK	Gastric Cancer
filter34	AGACUUUUUC	RNCMPT00268	CUUUUCU	PTBP1	Pancreatic Cancer

In future research about biomedical, we have two directions. One is to collect more data related to circRNAs, proteins, genes, and diseases from existing databases, literature, and other materials and use algorithms to discover disease-related regulatory information from these data. Another is to cooperate with hospitals to obtain and analyze disease-related sequence data, gene expression data, etc. with patients’ consent. By comparing the conclusions of the two directions, we can confirm the known disease-related gene expression regulation information, and it is possible to discover new disease-related gene expression regulation pathways and identify potential targets for cancer.

### Motif distribution analysis

**Fig. 3:**
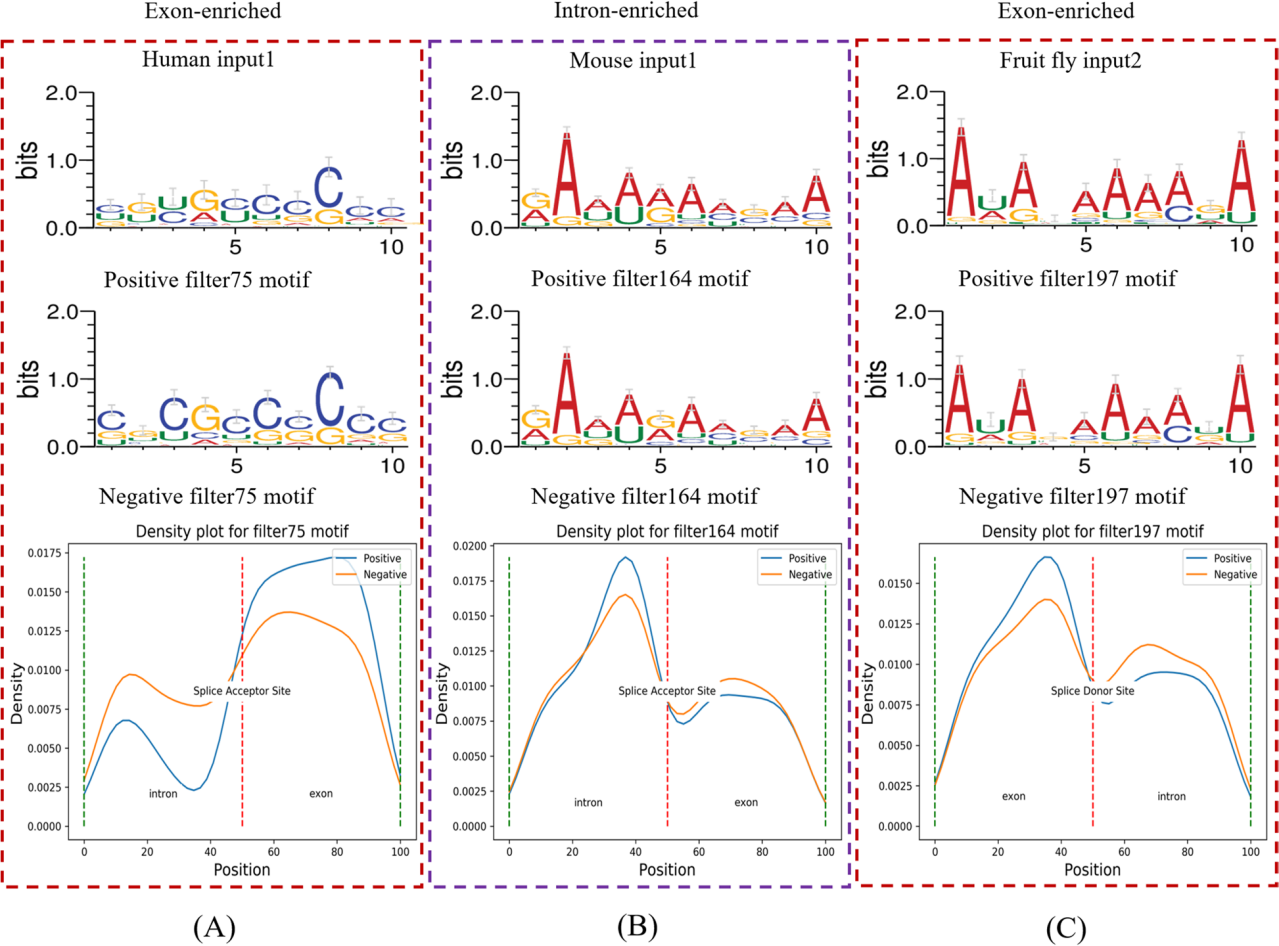
Distributions of RNA motifs found by CircCNN in the positive and negative samples. Two red bordered squares represents exon-enriched motif and its distribution, the purple bordered squares represents intron-enriched motif and its distributions. For the motif distribution plot, the red line represents splice acceptor site or splice donor site, blue line and orange line represents positive samples and negative samples respectively. For the red line in motif distribution plot (**A** and **B**), its left and right are intron and exon respectively. For the red line in motif distribution plot (**C**), its left and right are exon and intron respectively

Motif-related features are important to identify whether the current sequence contains back-splicing sites. In this section, RNA motifs were obtained by CircCNN from positive samples (Supplementary Tables S9). We analyzed the positive and negative samples for RNA motif distribution. The motif distribution pattern in three datasets is shown in supplementary Fig. S2 to S7. All RNA motifs found by CircCNN exist in positive and negative samples, and different motifs have different distribution patterns. For example, in human input1(SA input), the filter75 motif often appears in the flanking exon in the positive samples, its density in the flanking exon in the negative samples is much lower than in the positive samples, and the situation is reversed in the upstream intron (Fig. 3A). For filter164 motif (mouse input1), it is enriched in the upstream intron in the positive samples (Fig. 3B). In fruit fly input2 (SD input), density of filter197 motif in the flanking exon is lower in the negative samples than in the positive samples (Fig. 3C).

Previous study indicated that RNA motifs related CircRNA formation were often found in the flanking introns [40, 41]. In this study, we count the distribution of RNA motifs found by CircCNN. The ratio of motifs in the flanking introns and the flanking exons is basically around 50%. Table 4 and Supplementary Table S3 to S8 shows RNA motifs match known RNA motif database. From the density plot of these motifs, we found that it is often found in both the flanking introns and the flanking exons. Generally speaking, the flanking exons are also important for CircRNA formation.

**Fig. 4:**
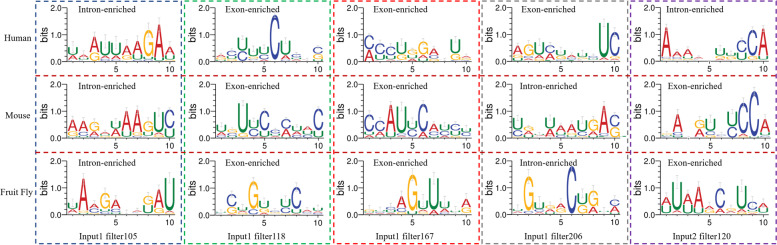
Sequence logos of several RNA motifs shared in three species. Here, three filters in three species are intron-enriched, exon-enriched and exon-enriched respectively. For filter 206(input1), it is exon-enriched motif in human and is intron-enriched motif in mouse and fruit fly. For filter120(input2), it is intron-enriched motif in human and is exon-enriched motif in mouse and fruit fly

In this study, CircCNN was also used to extract motifs from mouse and fruit fly CircRNA samples (Table 4 and Supplementary Table S3 to S8). We compared all RNA motifs found by CircCNN from human, mouse and fruit fly CircRNA samples. The comparison results were shown in Supplementary Tables S10 and S11. For input1 (SA input), 25 of 256 human motifs were also found in mouse motifs, 20 human motifs were also found in fruit fly motifs. For input2(SD input), 25 of 256 human motifs were also found in mouse motifs, 19 human motifs were also found in fruit fly motifs. From Table 6 and Fig. 4, we found that five RNA motifs are present in three species: human, mouse, and fruit fly. For human motifs, filter105(input1) and filter120(input2) were enriched in the flanking introns. Filter118(input1), filter167(input1) and filter206(input1) were enriched in the flanking exons. For mouse and fruit flies, filter105(input1) and filter206(input1) were enriched in the flanking introns, filter118(input1), filter167(input1), and filter120(input2) were enriched in the flanking exons. These motifs may be important for the conserved CircRNA formation.

**Table 6:** Several RNA motifs shared between human, mouse, and fruit fly

	FilterID	Human motif sequence	Mouse motif sequence	Fruit Fly motif sequence
Input1	filter105	UAAUUAAGAA	AAGAUAAGUC	UAAGAGAGAU
	filter118	ACUUUCUCAC	UGUUCCCUAC	UCUGUCUCAU
	filter167	CCCUGGAUUA	CCAUUCAUCU	GUCAGUUUUA
	filter206	AGUCUAUCUC	UGUUAAUGAC	UGUGACUGUC
Input2	filter120	AAAAAUUCCA	GAUGUCUCCA	AUAAACGUCA

## Conclusion

As special non-coding RNAs, Circular RNAs play a critical role in complex biological processes. Studying the regulation mechanism of CircRNA formation can reveal the function of CircRNAs in gene expression and disease development. Back-splicing is the key step in circRNA formation. In this study, we propose circCNN to predict the back-splicing sites of CircRNA formation. Unlike existing prediction methods, CircCNN uses two feature learning modules to extract features from SA input and SD input respectively. Batch-normalization is also used in CircCNN to improve model performance. The features captured by the convolution layer can be converted as motifs logos.

We perform CircCNN on three datasets. Experimental results show that CircCNN achieves the best results compared with other baseline models. Further analysis indicated that pre-mRNA back-splicing is controlled by multiple sequence features, including the distributions of RNA motifs, special function short sequence, and complementary sequences.

Although CircCNN has good performance, the prediction of back-splicing sites still faces the following challenges. Firstly, many CircRNAs have the same start or end locations in the genome sequence. The back-splicing pattern of these CircRNAs is resemblances. For a deep learning-based model, it is difficult to distinguish CircRNAs with similar back-splicing patterns. Secondly, unlike linear RNAs, sequence data about CircRNAs is not full enough. This is not only unfavorable for feature learning but also unfavorable for large-scale pre-training model training about data encoding. In the future, sequence data about CircRNA should be collected to help model training， build pre-trained encoding models, and so on. In addition, identifying the back-splicing patterns from nucleotide resolution is another direction. This is similar to using Fully Convolutional Networks (FCN) in image Semantic Segmentation [42]. This idea requires not only very reliable CircRNA sequence data but also requires us to modify existing models [43, 44]. There is a lot of work to be done in the future. We hope that CircCNN and our future work can provide useful information for studying the back-splicing regulation of CircRNA formation.

## Materials and methods

**Fig. 5:**
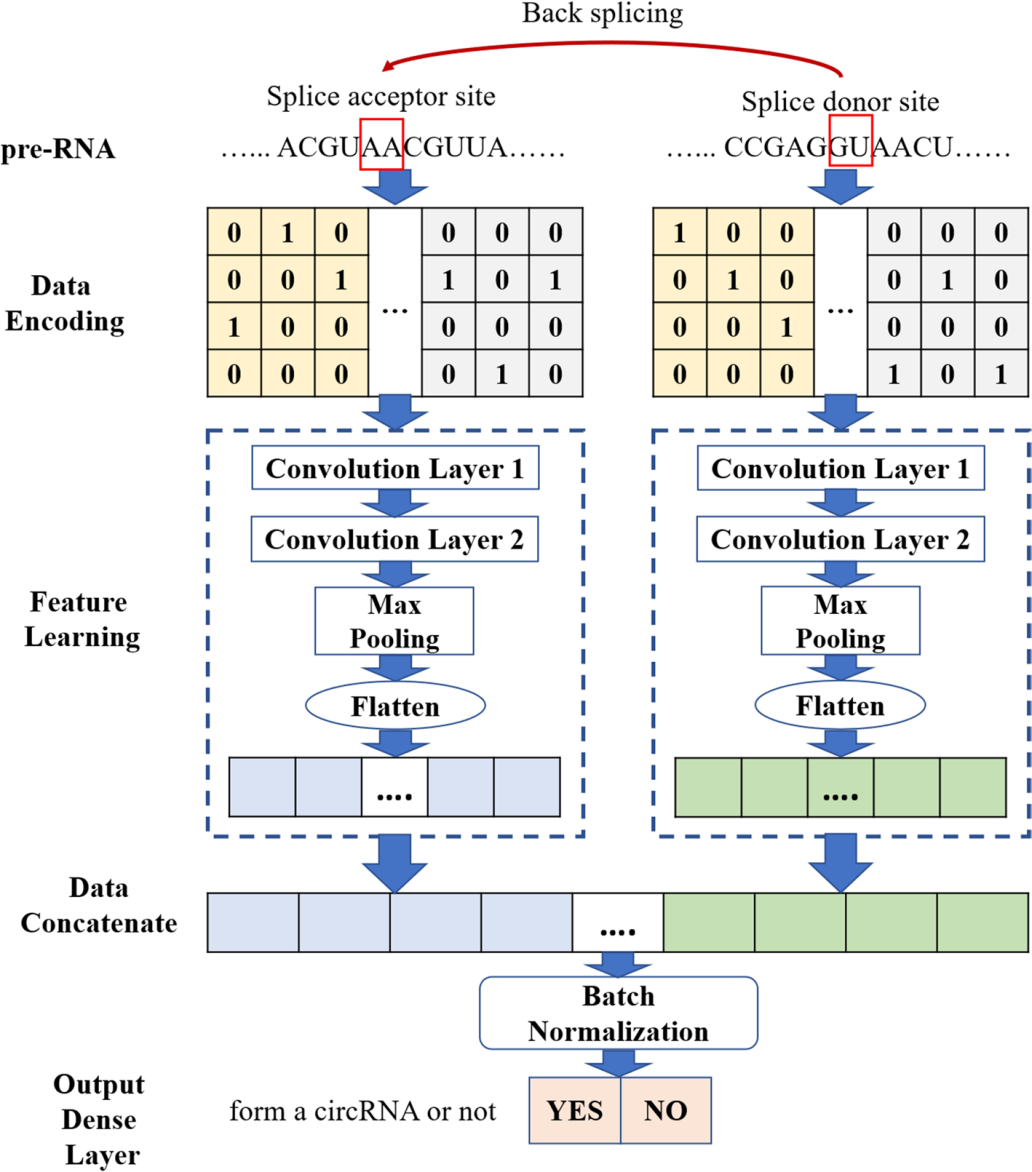
Workflow of CircCNN

CircCNN is a CNN-based model that extracts important back-splicing site features for CircRNA formation. CircCNN’s workflow is shown in Fig. 5. After converting the input sequence into a one-hot matrix, a CNN-based feature learning module is introduced. In the feature learning module, the first CNN layer detects potential motif sites and provides relevant data for visual motif sites. The second CNN layer can extract high-level abstract features. The most important features are selected by the max-pooling layer. After data concatenate and batch normalization, the final prediction result is obtained by the last dense layer.

Table 7 shows the feature shape output by each layer in CircCNN. Taking SA input as an example, for each input data, the shape of feature data extracted by the first conv layer in feature learning module is (89,256), which can be used to explore potential motif sites. The shape of feature data extracted by feature learning module is (1152). The shape of feature data extracted from SD input is consistent with SA input. The final feature data with shape (2304) is obtained by concatenating SA feature data and SD feature data, and will be sent to Dense layer to identify whether the current input data contains motif sites.

**Table 7:** The data output shape of each layer in CircCNN

	SA Input		SD Input	
Type	Layer	Output Shape	Layer	Output Shape
Input Layer	input_1	(None, 100,4)	input_2	(None, 100,4)
Conv1D	conv1	(None, 89,256)	conv3	(None, 89,256)
Dropout	dropout_1	(None, 89,256)	dropout_4	(None, 89,256)
Conv1D	Conv2	(None, 45,128)	conv4	(None, 45,128)
Dropout	dropout_2	(None, 45,128)	dropout_5	(None, 45,128)
MaxPooling 1D	max_pooling 1d_1	(None, 9128)	max_pooling 1d_2	(None, 9128)
Dropout	dropout_3	(None, 9128)	dropout_6	(None, 9128)
Flatten	flatten_1	(None, 1152)	flatten_2	(None, 1152)
Concatenate	cvout	(None, 2304)		
Batch Normalization	batch normalization_1	(None, 2304)		

“None” represents batch size

### Data

Three datasets we used in this paper are the same as DeepCirCode: human (GRCH37) [45, 46], mouse (GRCm38) [47], and fruit fly (BDGP5.4) [48]. CircRNA and back-splicing sites information of three datasets are shown in Table 8.

**Table 8:** Details about experimental data

Class	Source	
	Back splicing sites	circRNA datasets
Human	GRCH37, GTF	circRNADb (Ref45), circBase (Ref46)
Mouse	GRCm38, GTF	Ref47
Fruit Fly	BDGP5.4, GTF	Ref48

Take the human dataset as an example, CircRNA records were obtained from two databases CircRNADb and CircBase. The duplicated CircRNAs in the two databases were removed to improve dataset quality. If a CircRNA is only identified by one independent public study, it would be removed. The flanking sequence of two back-splicing sites was extracted by the genomic locations contained in CircRNA records. Consider the fact that what we want to do in this paper is find the back-splicing sites that are important to CircRNA formation, long flanking sequence may introduce interfering information, model efficiency is also reduced, and the input length of each input module (SA input and SD input) is fixed to 100. If the flanking intron (or exon) sequence length of SA or SD sites is less than 50 nt, this input sequence was not included in the final input data. Finally, 7964 human CircRNA sequences without any redundancy were obtained. To get the negative instance, one way is to randomly select a pair of canonical splicing sites covering 2–3 exons from the same transcript, and make sure this pair of splicing sites arenot in the positive sample. The method for processing human datasets is used for mouse data and fruit fly data. Using the same way already applied in the human dataset, 9953 mouse CircRNA instances and 5743 fruit fly CircRNA instances were generated. Details about three experimental datasets are shown in xlsx file Supplementary_Data2, which contain six sheets: human_positive, human_negative, mouse_positive, mouse_negative, fruit fly_positive, fruit fly_ negative (Supplementary Table S12 to S17), the training-to-test ratio is 4:1.

### Model structural

#### Data encoding

CircCNN has two input modules, SA input and SD input, with a fixed length of 100. For each set of input data, the one-hot method is used to encode the input sequence. Four nucleotides are represented as follows: *A*(1, 0, 0, 0), *C*(0, 1, 0, 0), *G*(0, 0, 1, 0), *U*(0, 0, 0, 1). Finally, each set of input data was converted to two 4*100 matrixes as the input of the feature learning module.

#### Feature learning module

Consider the fact that SA input and SD input are two kinds of circRNA sequence data representing different function, concatenating SA input and SD input and then using a convolution module to extract features, the feature information contained in SA input and SD input may interfere with each other, and affect the model prediction performance. Therefore, we use two feature learning modules. The internal structure of the feature learning module, which extracts features from SA sequence input and SD sequence input, is the same. Take the SA sequence inputs as an example.

Two convolution layers were introduced at first. The convolution kernel of the first convolution layer was used as a motif scanner to extract potential motif features from the input data. Its output and weights help us get visual motif sites. High-level abstract features extracted by the second convolution layer are important for identifying back-splicing or not.

1 $$ConvOut=Conv2\left(Conv1\left(SA\circ ker1,b\_1\right)\circ ker2,b\_2\right)$$

Where, *SA* represents input SA sequence data, *ker*1 and *ker*2 represents the convolution kernel of *Conv*1 and *Conv*2 respectively, *b* _ 1 and *b* _ 2 represents the bias term of *Conv*1 and *Conv*2 respectively.

The max-pooling layer can reduce the dimensionality of convolution layer output and select important features from *ConvOut*. The role of flatten layer is to convert multi-dimensional data into one vector. For example, if the input data dimension of flatten layer is: (none, 1,10,64), the output of flatten layer is one vector: (none, 640).

2 $$SA\_ out= flatten\left( MaxPooling(ConvOut)\right)$$

*SD* _ *out*was obtained by applying the above process to SD input data. The next work we need to do is to concatenate *SA* _ *out* and *SD* _ *out*. The role of batch normalization (BN) in CircCNN is to keep input data in the same distribution and avoid vanishing gradient, and overfitting.

3 $$BNOut= Bat\_ Norm\left( Concatenate\left( SA\_ out, SD\_ out\right)\right)$$

Identifying the back-splicing sites for CircRNA formation could be treated as a binary classification. The output layer of CircCNN was a fully connected layer with a sigmoid function. *BNOut* was fed into this layer to calculate the probability, which represents whether the input data contain back-splicing sites or not.

## Supplementary Information

**Additional file 1.**

**Additional file 2.**

## Data Availability

The datasets and source code of this study are freely available at: https://github.com/szhh521/CircCNN.
