# Supplementary material for: Prediction of Back-splicing sites for CircRNA formation based on convolutional neural networks
Source: BMC Genomics. 2022 Aug 12;23:581. doi: 10.1186/s12864-022-08820-1 (PMC9373444; doi:10.1186/s12864-022-08820-1)
Supplement: Supplementary file 1 — Additional file 1. [file 12864_2022_8820_MOESM1_ESM.docx]

**Supplementary data for**

**Prediction of Back-Splicing Sites for CircRNA Formation Based on Convolutional Neural Networks**

Zhen Shen^1^, YanLing Shao^1^, Wei Liu^1^, Qinhu Zhang^2,3^ and Lin Yuan^4*^

^1^School of Computer and Software, Nanyang Institute of Technology, Changjiang Road 80, Nanyang, Henan 473004, China. ^2^Translational Medical Center for Stem Cell Therapy and Institute for Regenerative Medicine, Shanghai East Hospital, Bioinformatics Department, School of Life Sciences and Technology, Tongji University, Siping Road 1239, Shanghai 200092, China. ^3^Institute of Machine Learning and Systems Biology, School of Electronics and Information Engineering, Tongji University, Caoan Road 4800, Shanghai 201804, China.^4^School of Computer Science and Technology, Qilu University of Technology (Shandong Academy of Sciences), Daxue Road 3501, Jinan, Shandong 250353, China.

*To whom correspondence should be addressed.

**Section 1** **k-fold Cross-Validation**

K-Fold cross-validation is where a given data set is split into a K number of sections/folds where each fold is used as a testing set at some point. Let’s take the scenario of 7-Fold cross validation(K=7). Here, the data set is split into 7 folds. In the first iteration, the first fold is used to test the model and the rest are used to train the model. In the second iteration, 2nd fold is used as the testing set while the rest serve as the training set. This process is repeated until each fold of the 7 folds have been used as the testing set.

The performance measure reported by k-fold cross-validation is then the average of the values computed in the loop. This approach can be computationally expensive, but does not waste too much data (as is the case when fixing an arbitrary validation set), which is a major advantage in problems, such as inverse inference, binary classification, where the number of samples is very small.

As we all known, deep learning model requires a large amount of data to obtain better model performance through feature learning. In this study, the number of three datasets (human, mouse, fruit fly) are 14696, 19905, and 11485, respectively. For deep learning, the number of the records of three species datasets is not a lot. Therefore, cross-validation is a feasible approach when the number of data records is limited. According to the definition of K-Fold cross-validation, the number of data records in each fold and K-fold are negatively correlated. If we use 5-fold cross-validation, the number of data used for model training is small, which is not conducive to feature learning. If we use 10-fold cross-validation, the number of data used for model testing is small and cannot reflect the real data composition, so it cannot show the real performance of our model. Above all, we use 7-fold cross-validation in our paper. To ensure fairness, not only CircCNN, but other baseline models also use 7-fold cross-validation.

**Section 2** **Confusion Matrix**

The confusion matrix, also known as the error matrix, is mainly used for statistical classification. It is a specific table layout that allows visualization of the performance of an algorithm. Each row of the matrix represents an instance in a predicted value while the column represents the actual value, or vice versa. The output matrix has four cells, true positive (TP), true negative (TN), false positive (FP), and false negative (FN). TP means the actual value and the predicted value are both positive, TN means the actual value is positive but the model predicted value is negative, FP means the actual value is negative but the model predicted value is positive, and, finally, FN means both the actual and predicted values are negative. In this study, five metrics were used to evaluate model performance: ACC(Accuracy), Sensitivity (also known as True Positive Rate, TPR), Specificity, MCC (Matthews correlation coefficient), and AUC (Area under the ROC curve).

ACC represents the proportion of all prediction results that are predicted correctly (See Eq.1). Sensitivity, represents the positive correctly classified samples to the total number of positive samples (See Eq.2). Specificity represents the ratio of the correctly classified negative samples to the total number of negative samples (See Eq.3). The value of MCC (See Eq. 4) represents the correlation between model predict results and the known labels (0: random guess, 1: perfect model). AUC is often used to evaluate model performance, and the value is between 0.5 and 1. 0.5 denotes model output is random guess, 1 denotes model performance is perfect.


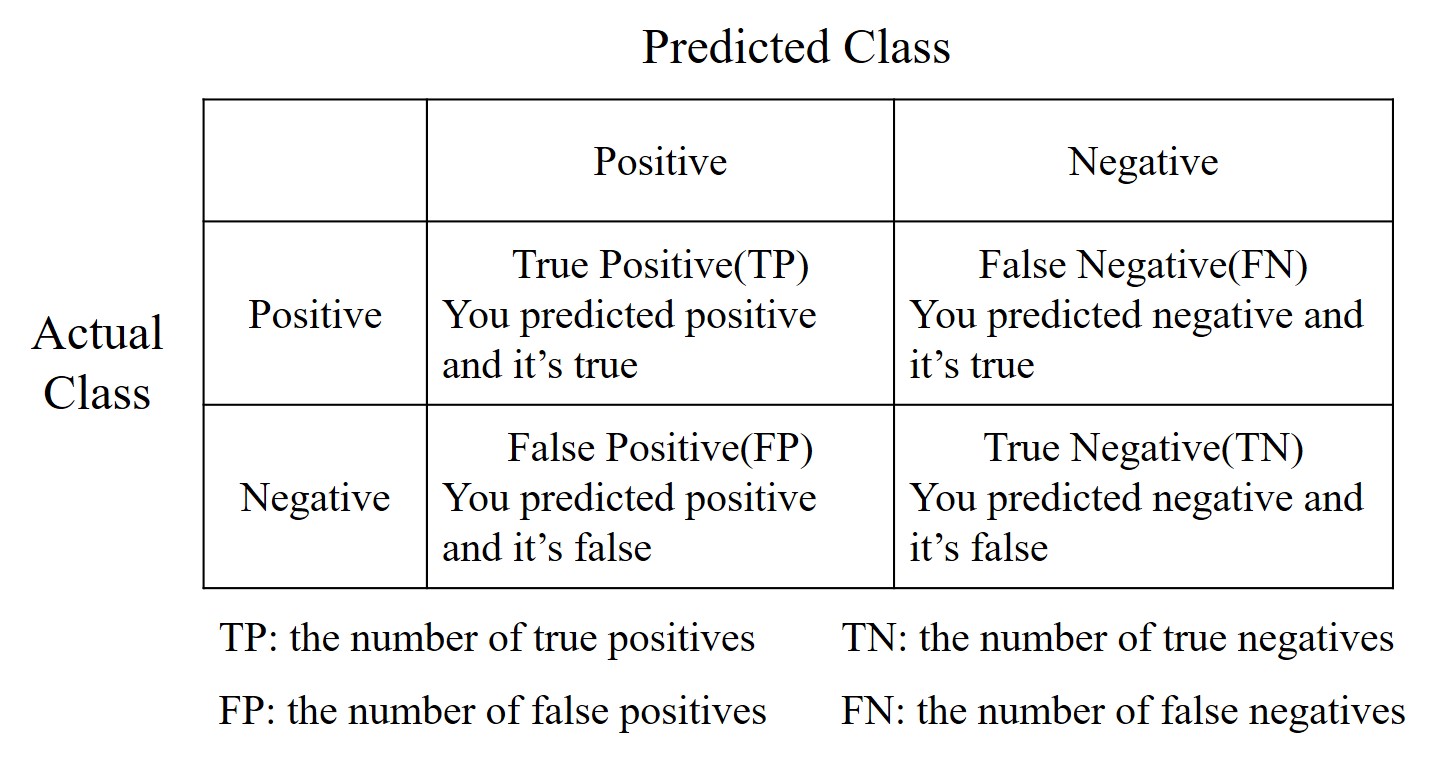


**Figure S1.** Confusion matrix.

**Section 3 Supplementary data for experimental results**

**Table S1.** Performance comparison of CircCNN with different parameter combinations

|  | Human | | | | | Mouse | | | | | Fruit Fly | | | | |
| --- | --- | --- | --- | --- | --- | --- | --- | --- | --- | --- | --- | --- | --- | --- | --- |
|  | AUC | ACC | MCC | Sens | Spec | AUC | ACC | MCC | Sens | Spec | AUC | ACC | MCC | Sens | Spec |
| 10,20,1,0.2 | 0.882 | 0.8205 | 0.6402 | 0.8875 | 0.7412 | 0.8159 | 0.7412 | 0.4877 | 0.782 | 0.6775 | 0.8288 | 0.744 | 0.4963 | 0.8114 | 0.6766 |
| 10,20,1,0.5 | 0.8992 | 0.8359 | 0.6719 | 0.9063 | 0.7525 | 0.8473 | 0.765 | 0.5433 | 0.8604 | 0.6566 | 0.8611 | 0.772 | 0.5499 | 0.8312 | 0.7027 |
| 10,20,1,0.7 | 0.903 | 0.8414 | 0.68 | 0.9131 | 0.7561 | 0.8487 | 0.7683 | 0.5483 | 0.8582 | 0.6779 | 0.8695 | 0.7866 | 0.5758 | 0.826 | 0.7271 |
| 10,20,2,0.2 | 0.8831 | 0.8152 | 0.629 | 0.866 | 0.755 | 0.8126 | 0.7324 | 0.4672 | 0.7391 | 0.6757 | 0.8319 | 0.7417 | 0.4981 | 0.8326 | 0.6437 |
| 10,20,2,0.5 | 0.9015 | 0.8387 | 0.6781 | 0.9102 | 0.754 | 0.8423 | 0.7659 | 0.5405 | 0.8513 | 0.6765 | 0.8608 | 0.7726 | 0.5556 | 0.8352 | 0.6799 |
| 10,20,2,0.7 | 0.9045 | 0.8368 | 0.6847 | 0.9128 | 0.7536 | 0.849 | 0.7692 | 0.5498 | 0.8591 | 0.6673 | 0.8703 | 0.7837 | 0.5734 | 0.8281 | 0.7353 |
| 10,30,1,0.2 | 0.8807 | 0.8208 | 0.6413 | 0.8877 | 0.7417 | 0.8197 | 0.7371 | 0.4785 | 0.7339 | 0.6784 | 0.8244 | 0.734 | 0.4854 | 0.8276 | 0.6404 |
| 10,30,1,0.5 | 0.8981 | 0.8387 | 0.6806 | 0.9134 | 0.742 | 0.8449 | 0.7578 | 0.5318 | 0.8551 | 0.6405 | 0.8607 | 0.7714 | 0.5535 | 0.8348 | 0.6879 |
| 10,30,1,0.7 | 0.9032 | 0.8381 | 0.6782 | 0.912 | 0.7521 | 0.8505 | 0.7672 | 0.5502 | 0.8578 | 0.6735 | 0.8697 | 0.7797 | 0.5741 | 0.8234 | 0.7326 |
| 10,30,2,0.2 | 0.887 | 0.8164 | 0.6332 | 0.8715 | 0.7512 | 0.8138 | 0.7309 | 0.464 | 0.7223 | 0.6696 | 0.8306 | 0.7478 | 0.5025 | 0.8073 | 0.6884 |
| 10,30,2,0.5 | 0.903 | 0.8404 | 0.682 | 0.9139 | 0.7536 | 0.8449 | 0.7581 | 0.5326 | 0.8527 | 0.6394 | 0.8607 | 0.7754 | 0.5548 | 0.8307 | 0.7201 |
| 10,30,2,0.7 | 0.9042 | 0.8398 | 0.7025 | 0.9129 | 0.7411 | 0.8493 | 0.7702 | 0.5492 | 0.8608 | 0.6756 | 0.8684 | 0.7856 | 0.5752 | 0.8337 | 0.7344 |
| 10,40,1,0.2 | 0.8828 | 0.8172 | 0.6315 | 0.8644 | 0.7543 | 0.8189 | 0.7405 | 0.4838 | 0.7729 | 0.6788 | 0.8324 | 0.7378 | 0.4969 | 0.8271 | 0.6135 |
| 10,40,1,0.5 | 0.9026 | 0.8355 | 0.6736 | 0.9091 | 0.7485 | 0.8331 | 0.7465 | 0.5067 | 0.8325 | 0.6605 | 0.859 | 0.7708 | 0.5499 | 0.8318 | 0.6909 |
| 10,40,1,0.7 | 0.9035 | 0.8357 | 0.6831 | 0.9079 | 0.7493 | 0.8507 | 0.7693 | 0.5504 | 0.8552 | 0.6759 | 0.8702 | 0.7786 | 0.5709 | 0.837 | 0.7302 |
| 10,40,2,0.2 | 0.8862 | 0.8229 | 0.6466 | 0.8998 | 0.732 | 0.8125 | 0.729 | 0.4632 | 0.7074 | 0.6746 | 0.8354 | 0.7451 | 0.4994 | 0.8233 | 0.667 |
| 10,40,2,0.5 | 0.903 | 0.8378 | 0.6773 | 0.9129 | 0.7491 | 0.8421 | 0.7545 | 0.5258 | 0.8574 | 0.6316 | 0.8585 | 0.7728 | 0.5527 | 0.8342 | 0.6985 |
| 10,40,2,0.7 | 0.9046 | 0.8403 | 0.6789 | 0.9137 | 0.7503 | 0.8489 | 0.7705 | 0.5471 | 0.8607 | 0.6783 | 0.8689 | 0.7832 | 0.5726 | 0.8177 | 0.7336 |
| 12,20,1,0.2 | 0.8804 | 0.8156 | 0.6295 | 0.8771 | 0.7429 | 0.8182 | 0.7324 | 0.4682 | 0.6965 | 0.6684 | 0.832 | 0.7403 | 0.4985 | 0.8294 | 0.6311 |
| 12,20,1,0.5 | 0.8979 | 0.8408 | 0.6842 | 0.9133 | 0.7408 | 0.8436 | 0.7572 | 0.5336 | 0.8598 | 0.6306 | 0.8599 | 0.773 | 0.5531 | 0.8351 | 0.7109 |
| 12,20,1,0.7 | 0.898 | 0.8414 | 0.6817 | 0.914 | 0.7458 | 0.8501 | 0.7703 | 0.5439 | 0.8573 | 0.6632 | 0.869 | 0.7825 | 0.5689 | 0.8367 | 0.7283 |
| 12,20,2,0.2 | 0.8806 | 0.8127 | 0.6237 | 0.8634 | 0.7527 | 0.8162 | 0.7381 | 0.4787 | 0.7437 | 0.6725 | 0.8309 | 0.7399 | 0.4941 | 0.8335 | 0.6463 |
| 12,20,2,0.5 | 0.8993 | 0.8387 | 0.6789 | 0.9138 | 0.7475 | 0.8433 | 0.7645 | 0.541 | 0.8606 | 0.661 | 0.8582 | 0.7748 | 0.5542 | 0.8367 | 0.7128 |
| 12,20,2,0.7 | 0.9028 | 0.8365 | 0.6827 | 0.9118 | 0.7503 | 0.8493 | 0.7675 | 0.5458 | 0.8562 | 0.6789 | 0.8692 | 0.7819 | 0.5682 | 0.8301 | 0.7317 |
| 12,30,1,0.2 | 0.8855 | 0.8204 | 0.6393 | 0.8668 | 0.7525 | 0.8213 | 0.7408 | 0.4838 | 0.7365 | 0.6751 | 0.834 | 0.7363 | 0.4894 | 0.8339 | 0.6256 |
| 12,30,1,0.5 | 0.8978 | 0.8356 | 0.6741 | 0.9133 | 0.7378 | 0.8444 | 0.7607 | 0.5324 | 0.8538 | 0.6676 | 0.8572 | 0.7702 | 0.5464 | 0.8311 | 0.7094 |
| 12,30,1,0.7 | 0.901 | 0.8386 | 0.6821 | 0.9119 | 0.7545 | 0.8506 | 0.7702 | 0.5466 | 0.8612 | 0.6738 | 0.867 | 0.7854 | 0.5754 | 0.8324 | 0.7283 |
| 12,30,2,0.2 | 0.8832 | 0.8184 | 0.6351 | 0.8812 | 0.7441 | 0.8179 | 0.7312 | 0.4679 | 0.6847 | 0.6777 | 0.8231 | 0.7354 | 0.4844 | 0.8245 | 0.6463 |
| 12,30,2,0.5 | 0.901 | 0.8389 | 0.6788 | 0.9117 | 0.7527 | 0.8398 | 0.7538 | 0.5226 | 0.8592 | 0.6425 | 0.8608 | 0.7715 | 0.5508 | 0.8326 | 0.693 |
| **12,30,2,0.7** | **0.9049** | **0.8421** | **0.6849** | **0.9147** | **0.7562** | **0.8514** | **0.7705** | **0.5508** | **0.8614** | **0.6797** | **0.8708** | **0.7869** | **0.5773** | **0.8374** | **0.7365** |
| 12,40,1,0.2 | 0.8779 | 0.8138 | 0.6268 | 0.8777 | 0.7383 | 0.8174 | 0.7333 | 0.469 | 0.7011 | 0.6655 | 0.8343 | 0.7435 | 0.4972 | 0.7675 | 0.7194 |
| 12,40,1,0.5 | 0.8993 | 0.8371 | 0.6764 | 0.9136 | 0.7442 | 0.8449 | 0.7563 | 0.5253 | 0.8608 | 0.6509 | 0.8591 | 0.7701 | 0.5477 | 0.837 | 0.7032 |
| 12,40,1,0.7 | 0.9011 | 0.8358 | 0.6822 | 0.9338 | 0.7417 | 0.8499 | 0.7704 | 0.5505 | 0.8564 | 0.6743 | 0.8659 | 0.7848 | 0.571 | 0.8158 | 0.7335 |
| 12,40,2,0.2 | 0.8885 | 0.8195 | 0.6365 | 0.8651 | 0.7536 | 0.8137 | 0.7315 | 0.4653 | 0.7056 | 0.6574 | 0.8308 | 0.7424 | 0.4943 | 0.8182 | 0.6665 |
| 12,40,2,0.5 | 0.9017 | 0.8369 | 0.6744 | 0.9031 | 0.7586 | 0.8419 | 0.7542 | 0.5221 | 0.8609 | 0.6456 | 0.8581 | 0.7744 | 0.5515 | 0.8147 | 0.7341 |
| 12,40,2,0.7 | 0.9038 | 0.8413 | 0.6811 | 0.9137 | 0.7543 | 0.8501 | 0.7684 | 0.5449 | 0.8508 | 0.6861 | 0.8659 | 0.7846 | 0.5763 | 0.8314 | 0.7317 |
| 15,20,1,0.2 | 0.8814 | 0.8151 | 0.628 | 0.8682 | 0.7522 | 0.8143 | 0.7344 | 0.473 | 0.7105 | 0.6583 | 0.8351 | 0.7492 | 0.5062 | 0.8306 | 0.6677 |
| 15,20,1,0.5 | 0.8964 | 0.8354 | 0.6717 | 0.9096 | 0.7476 | 0.8452 | 0.7613 | 0.535 | 0.8637 | 0.6589 | 0.8536 | 0.7646 | 0.5365 | 0.8368 | 0.6923 |
| 15,20,1,0.7 | 0.895 | 0.8416 | 0.6838 | 0.9146 | 0.7552 | 0.8497 | 0.7697 | 0.5502 | 0.8691 | 0.6702 | 0.8663 | 0.7857 | 0.5735 | 0.8194 | 0.7352 |
| 15,20,2,0.2 | 0.8813 | 0.8089 | 0.616 | 0.8498 | 0.7495 | 0.8167 | 0.7313 | 0.4694 | 0.7125 | 0.675 | 0.8407 | 0.7449 | 0.5033 | 0.8274 | 0.6403 |
| 15,20,2,0.5 | 0.8944 | 0.8357 | 0.6736 | 0.9109 | 0.7374 | 0.8394 | 0.7574 | 0.5296 | 0.8612 | 0.6437 | 0.8581 | 0.7699 | 0.545 | 0.837 | 0.7027 |
| 15,20,2,0.7 | 0.9001 | 0.8372 | 0.6784 | 0.9126 | 0.7491 | 0.8463 | 0.7695 | 0.5491 | 0.8522 | 0.6667 | 0.8674 | 0.787 | 0.5755 | 0.8357 | 0.7283 |
| 15,30,1,0.2 | 0.8824 | 0.8165 | 0.6312 | 0.8762 | 0.746 | 0.8165 | 0.7392 | 0.4823 | 0.7543 | 0.6741 | 0.8272 | 0.7452 | 0.4951 | 0.7869 | 0.7036 |
| 15,30,1,0.5 | 0.8965 | 0.8364 | 0.6756 | 0.9125 | 0.7346 | 0.8424 | 0.7537 | 0.5226 | 0.8603 | 0.6452 | 0.8557 | 0.7624 | 0.5369 | 0.8337 | 0.659 |
| 15,30,1,0.7 | 0.8993 | 0.8395 | 0.6805 | 0.9135 | 0.7502 | 0.8485 | 0.7684 | 0.5454 | 0.847 | 0.6679 | 0.8649 | 0.7843 | 0.5714 | 0.8224 | 0.7463 |
| 15,30,2,0.2 | 0.8838 | 0.8174 | 0.6362 | 0.8812 | 0.742 | 0.8083 | 0.7297 | 0.4623 | 0.7413 | 0.6718 | 0.8308 | 0.745 | 0.4985 | 0.8285 | 0.6614 |
| 15,30,2,0.5 | 0.8957 | 0.8379 | 0.6792 | 0.9093 | 0.7334 | 0.8368 | 0.7543 | 0.5232 | 0.8576 | 0.6411 | 0.8574 | 0.7683 | 0.543 | 0.8322 | 0.6984 |
| 15,30,2,0.7 | 0.9026 | 0.8419 | 0.6795 | 0.9128 | 0.7519 | 0.8481 | 0.7689 | 0.5466 | 0.8442 | 0.6655 | 0.8643 | 0.7804 | 0.5649 | 0.8325 | 0.7283 |
| 15,40,1,0.2 | 0.8839 | 0.8158 | 0.632 | 0.8697 | 0.7521 | 0.8203 | 0.7387 | 0.4795 | 0.7517 | 0.6757 | 0.8348 | 0.7416 | 0.4989 | 0.8287 | 0.6334 |
| 15,40,1,0.5 | 0.8964 | 0.837 | 0.6789 | 0.9097 | 0.7249 | 0.84 | 0.7537 | 0.5187 | 0.8442 | 0.6632 | 0.856 | 0.7702 | 0.5452 | 0.8174 | 0.7231 |
| 15,40,1,0.7 | 0.8995 | 0.8389 | 0.6785 | 0.9127 | 0.7515 | 0.8462 | 0.7642 | 0.5359 | 0.843 | 0.6755 | 0.8637 | 0.7797 | 0.5609 | 0.8151 | 0.7343 |
| 15,40,2,0.2 | 0.8857 | 0.8178 | 0.6337 | 0.864 | 0.7531 | 0.8136 | 0.733 | 0.4693 | 0.7471 | 0.6589 | 0.8402 | 0.756 | 0.5197 | 0.8297 | 0.6823 |
| 15,40,2,0.5 | 0.8971 | 0.8357 | 0.6735 | 0.9143 | 0.7393 | 0.8383 | 0.7528 | 0.521 | 0.8593 | 0.6364 | 0.8565 | 0.7741 | 0.5529 | 0.8367 | 0.7114 |
| 15,40,2,0.7 | 0.9014 | 0.8395 | 0.6823 | 0.9137 | 0.7522 | 0.8464 | 0.7668 | 0.5401 | 0.8394 | 0.6742 | 0.8644 | 0.7828 | 0.5681 | 0.8132 | 0.7324 |

**Table S2.** Comparison of CircCNN and other baseline models in cross-validation

| Model | Human | | | Mouse | | | Fruit Fly | | |
| --- | --- | --- | --- | --- | --- | --- | --- | --- | --- |
|  | MCC | Sens | Spec | MCC | Sens | Spec | MCC | Sens | Spec |
| Model① | 0.6023 | 0.8795 | 0.7102 | 0.5403 | 0.8396 | 0.6942 | 0.5542 | 0.8182 | 0.7328 |
| Model② | 0.3408 | 0.7967 | 0.5297 | 0.4119 | 0.731 | 0.6797 | 0.4077 | 0.7425 | 0.6635 |
| Model③ | 0.5568 | 0.8587 | 0.6852 | 0.5097 | 0.8115 | 0.6934 | 0.5327 | 0.8609 | 0.6576 |
| Model④ | 0.5228 | 0.8193 | 0.6943 | 0.4406 | 0.7311 | 0.7065 | 0.4869 | 0.7519 | 0.7278 |
| Model⑤ | 0.3285 | 0.7833 | 0.5243 | 0.3303 | 0.7031 | 0.6243 | 0.3567 | 0.747 | 0.605 |
| DeepCircCode | 0.6449 | 0.8864 | 0.7485 | 0.5383 | 0.8473 | 0.6833 | 0.5635 | 0.837 | 0.7222 |
| CircCNN(CVLD) | 0.6818 | 0.9297 | 0.7415 | 0.5321 | 0.859 | 0.6641 | 0.571 | 0.8124 | 0.7667 |
| CircCNN | **0.6849** | 0.9147 | 0.7562 | **0.5508** | 0.8614 | 0.6797 | **0.5773** | 0.8374 | 0.7365 |

**Table S3.** Motifs found by CircCNN from input1data for human back-splicing prediction match the known human RNA motifs in the RNA/Ray2013_rbp_Homo_sapiens.meme

| FilterID | Motif found by CircCNN | Known motif in database | Known motif sequence | Gene Annotation | E-value |
| --- | --- | --- | --- | --- | --- |
| filter9 | GAGAAAGUUA | RNCMPT00090 | AGAGAAA | SRSF10 | 0.0638 |
|  | GAGAAAGUUA | RNCMPT00019 | AGAGAAA | SRSF10 | 0.0867 |
| filter16 | AUUUAUUUUA | RNCMPT00032 | UUAUUUU | HuR | 0.0115 |
|  | AUUUAUUUUA | RNCMPT00167 | AUUUUUU | HNRNPCL1 | 0.1513 |
|  | AUUUAUUUUA | RNCMPT00274 | UUUUUUU | HuR | 0.1692 |
|  | AUUUAUUUUA | RNCMPT00025 | AUUUUUU | HNRNPC | 0.1692 |
|  | AUUUAUUUUA | RNCMPT00012 | CUUUUUU | CPEB2 | 0.2388 |
|  | AUUUAUUUUA | RNCMPT00053 | AUACAUU | RBM41 | 0.3852 |
|  | AUUUAUUUUA | RNCMPT00158 | CUUUUUU | CPEB4 | 0.3852 |
| filter36 | UCUCUUUUUG | RNCMPT00012 | CUUUUUU | CPEB2 | 0.0205 |
|  | UCUCUUUUUG | RNCMPT00158 | CUUUUUU | CPEB4 | 0.0247 |
|  | UCUCUUUUUG | RNCMPT00274 | UUUUUUU | HuR | 0.0603 |
|  | UCUCUUUUUG | RNCMPT00268 | CUUUUCU | PTBP1 | 0.1156 |
|  | UCUCUUUUUG | RNCMPT00269 | ACUUUCU | PTBP1 | 0.2742 |
|  | UCUCUUUUUG | RNCMPT00165 | UUUUUUC | TIA1 | 0.2942 |
|  | UCUCUUUUUG | RNCMPT00077 | UUUUUUG | TIA1 | 0.3153 |
|  | UCUCUUUUUG | RNCMPT00079 | UUUUUUC | U2AF2 | 0.3153 |
|  | UCUCUUUUUG | RNCMPT00159 | UUUUUUG | RALY | 0.3153 |
|  | UCUCUUUUUG | RNCMPT00025 | AUUUUUU | HNRNPC | 0.4125 |
|  | UCUCUUUUUG | RNCMPT00167 | AUUUUUU | HNRNPCL1 | 0.4417 |
| filter60 | CUGAUAGUAA | RNCMPT00050 | GAUACGA | RBM3 | 0.0493 |
| filter63 | GAAAAAAAAA | RNCMPT00043 | AAAAAAA | PABPC4 | 0.0076 |
|  | GAAAAAAAAA | RNCMPT00064 | AGAAAAA | SART3 | 0.0217 |
|  | GAAAAAAAAA | RNCMPT00155 | AGAAAAA | PABPC1 | 0.0217 |
| filter70 | UUCCUUUUUU | RNCMPT00012 | CUUUUUU | CPEB2 | 0.0914 |
|  | UUCCUUUUUU | RNCMPT00053 | AUACAUU | RBM41 | 0.1196 |
|  | UUCCUUUUUU | RNCMPT00158 | CUUUUUU | CPEB4 | 0.2018 |
|  | UUCCUUUUUU | RNCMPT00186 | CCUUUCC | PCBP1 | 0.2018 |
|  | UUCCUUUUUU | RNCMPT00268 | CUUUUCU | PTBP1 | 0.2018 |
| filter72 | AAAAAAAUCU | RNCMPT00043 | AAAAAAA | PABPC4 | 0.0107 |
| filter73 | UACAGACCGG | RNCMPT00149 | CAGACAG | ENOX1 | 0.0474 |
| filter76 | AAAAAAAGUC | RNCMPT00043 | AAAAAAA | PABPC4 | 0.0044 |
| filter83 | AGAUAAACUA | RNCMPT00185 | GAUAAAA | KHDRBS2 | 0.038 |
| filter84 | UUAGUUUUUU | RNCMPT00158 | CUUUUUU | CPEB4 | 0.042 |
|  | UUAGUUUUUU | RNCMPT00012 | CUUUUUU | CPEB2 | 0.0944 |
|  | UUAGUUUUUU | RNCMPT00274 | UUUUUUU | HuR | 0.0944 |
|  | UUAGUUUUUU | RNCMPT00159 | UUUUUUG | RALY | 0.1058 |
|  | UUAGUUUUUU | RNCMPT00165 | UUUUUUC | TIA1 | 0.1058 |
|  | UUAGUUUUUU | RNCMPT00077 | UUUUUUG | TIA1 | 0.1305 |
|  | UUAGUUUUUU | RNCMPT00079 | UUUUUUC | U2AF2 | 0.1586 |
|  | UUAGUUUUUU | RNCMPT00025 | AUUUUUU | HNRNPC | 0.2102 |
|  | UUAGUUUUUU | RNCMPT00167 | AUUUUUU | HNRNPCL1 | 0.2302 |
|  | UUAGUUUUUU | RNCMPT00032 | UUAUUUU | HuR | 0.2302 |
|  | UUAGUUUUUU | RNCMPT00268 | CUUUUCU | PTBP1 | 0.3971 |
|  | UUAGUUUUUU | RNCMPT00269 | ACUUUCU | PTBP1 | 0.5156 |
| filter86 | GUUUGUGUGA | RNCMPT00086 | UUUGUUU | ZC3H14 | 0.1331 |
|  | GUUUGUGUGA | RNCMPT00166 | UGUGUGU | BRUNOL5 | 0.1466 |
|  | GUUUGUGUGA | RNCMPT00004 | UGUGUGU | BRUNOL4 | 0.1763 |
|  | GUUUGUGUGA | RNCMPT00112 | UUUGUUU | HuR | 0.1763 |
|  | GUUUGUGUGA | RNCMPT00117 | UUUGUUU | HuR | 0.1928 |
| filter91 | GACAAGGGGG | RNCMPT00016 | GGACAAG | FMR1 | 0.0415 |
| filter107 | ACAAAAACCU | RNCMPT00153 | GAAAACC | PABPC3 | 0.0357 |
|  | ACAAAAACCU | RNCMPT00033 | ACAAACA | IGF2BP2 | 0.0879 |
|  | ACAAAAACCU | RNCMPT00172 | ACAAACA | IGF2BP3 | 0.1555 |
| filter117 | GAAUCAAUUA | RNCMPT00054 | AAUCAAU | RBM46 | 0.0426 |
| filter118 | ACUUUCUCAC | RNCMPT00044 | CCUUCCC | PCBP2 | 0.0529 |
|  | ACUUUCUCAC | RNCMPT00269 | ACUUUCU | PTBP1 | 0.0593 |
| filter123 | UACGAUCCUG | RNCMPT00075 | CGAUACU | TUT1 | 0.0053 |
| filter157 | GUAACUUUCC | RNCMPT00269 | ACUUUCU | PTBP1 | 0.0482 |
| filter169 | UAGACACACA | RNCMPT00027 | ACACACA | HNRNPL | 0.0241 |
|  | UAGACACACA | RNCMPT00178 | ACACACA | HNRPLL | 0.0241 |
| filter184 | GAGAAAGAUU | RNCMPT00064 | AGAAAAA | SART3 | 0.099 |
|  | GAGAAAGAUU | RNCMPT00155 | AGAAAAA | PABPC1 | 0.099 |
| filter188 | AAGACCUUCC | RNCMPT00044 | CCUUCCC | PCBP2 | 0.0039 |
| filter189 | AAAAAAAAAA | RNCMPT00043 | AAAAAAA | PABPC4 | 0.0004 |
| filter215 | CUUUUAUUUU | RNCMPT00274 | UUUUUUU | HuR | 0.0127 |
|  | CUUUUAUUUU | RNCMPT00032 | UUAUUUU | HuR | 0.0198 |
|  | CUUUUAUUUU | RNCMPT00158 | CUUUUUU | CPEB4 | 0.1163 |
|  | CUUUUAUUUU | RNCMPT00268 | CUUUUCU | PTBP1 | 0.2516 |
|  | CUUUUAUUUU | RNCMPT00012 | CUUUUUU | CPEB2 | 0.2758 |
|  | CUUUUAUUUU | RNCMPT00086 | UUUGUUU | ZC3H14 | 0.2758 |
|  | CUUUUAUUUU | RNCMPT00112 | UUUGUUU | HuR | 0.2758 |
|  | CUUUUAUUUU | RNCMPT00117 | UUUGUUU | HuR | 0.3271 |
| filter218 | AAGAAAAUAA | RNCMPT00171 | AGAAAAU | PABPC5 | 0.0009 |
| filter224 | UAUACUUUUG | RNCMPT00053 | AUACAUU | RBM41 | 0.0284 |
| filter234 | GUUCAAGAUC | RNCMPT00070 | GAUCAAG | SNRNP70 | 0.047 |
| filter235 | UGUGUCUAGG | RNCMPT00166 | UGUGUGU | BRUNOL5 | 0.0698 |
|  | UGUGUCUAGG | RNCMPT00004 | UGUGUGU | BRUNOL4 | 0.0787 |
| filter239 | GGGAGAAGAC | RNCMPT00157 | AGAAGAC | PABPN1 | 0.0139 |
| filter243 | CAUAAAAAAA | RNCMPT00043 | AAAAAAA | PABPC4 | 0.0341 |
|  | CAUAAAAAAA | RNCMPT00169 | AUAAAAG | KHDRBS1 | 0.1907 |
|  | CAUAAAAAAA | RNCMPT00064 | AGAAAAA | SART3 | 0.2064 |
|  | CAUAAAAAAA | RNCMPT00155 | AGAAAAA | PABPC1 | 0.2064 |

**Table S4.** Motifs found by CircCNN from input2 data for human back-splicing prediction match the known human RNA motifs in the RNA/Ray2013_rbp_Homo_sapiens.meme

| FilterID | Motif found by CircCNN | Known motif in database | Known motif sequence | Gene Annotation | E-value |
| --- | --- | --- | --- | --- | --- |
| filter2 | CUUGGUUUCC | RNCMPT00136 | UUGGUUU | HuR | 0.028 |
|  | CUUGGUUUCC | RNCMPT00086 | UUUGUUU | ZC3H14 | 0.0766 |
|  | CUUGGUUUCC | RNCMPT00117 | UUUGUUU | HuR | 0.0766 |
|  | CUUGGUUUCC | RNCMPT00112 | UUUGUUU | HuR | 0.115 |
| filter15 | AGUACCUUAC | RNCMPT00186 | CCUUUCC | PCBP1 | 0.0418 |
| filter18 | CCAUUUUCUU | RNCMPT00269 | ACUUUCU | PTBP1 | 0.0133 |
|  | CCAUUUUCUU | RNCMPT00268 | CUUUUCU | PTBP1 | 0.0413 |
|  | CCAUUUUCUU | RNCMPT00164 | UGUUCGU | ZNF638 | 0.063 |
| filter28 | UUUUUUCCGA | RNCMPT00165 | UUUUUUC | TIA1 | 0.0103 |
|  | UUUUUUCCGA | RNCMPT00079 | UUUUUUC | U2AF2 | 0.0138 |
|  | UUUUUUCCGA | RNCMPT00274 | UUUUUUU | HuR | 0.1124 |
| filter34 | AGACUUUUUC | RNCMPT00268 | CUUUUCU | PTBP1 | 0.0043 |
|  | AGACUUUUUC | RNCMPT00158 | CUUUUUU | CPEB4 | 0.0052 |
|  | AGACUUUUUC | RNCMPT00269 | ACUUUCU | PTBP1 | 0.0252 |
|  | AGACUUUUUC | RNCMPT00012 | CUUUUUU | CPEB2 | 0.032 |
|  | AGACUUUUUC | RNCMPT00079 | UUUUUUC | U2AF2 | 0.0709 |
|  | AGACUUUUUC | RNCMPT00165 | UUUUUUC | TIA1 | 0.1594 |
|  | AGACUUUUUC | RNCMPT00274 | UUUUUUU | HuR | 0.273 |
|  | AGACUUUUUC | RNCMPT00159 | UUUUUUG | RALY | 0.3025 |
|  | AGACUUUUUC | RNCMPT00167 | AUUUUUU | HNRNPCL1 | 0.3334 |
|  | AGACUUUUUC | RNCMPT00077 | UUUUUUG | TIA1 | 0.3334 |
|  | AGACUUUUUC | RNCMPT00025 | AUUUUUU | HNRNPC | 0.3662 |
|  | AGACUUUUUC | RNCMPT00044 | CCUUCCC | PCBP2 | 0.6067 |
| filter56 | UAUCAAAAAA | RNCMPT00043 | AAAAAAA | PABPC4 | 0.0468 |
| filter72 | AUAAGUAGAC | RNCMPT00001 | AUAAUUG | A1CF | 0.0305 |
| filter73 | UCUUUCUUUA | RNCMPT00268 | CUUUUCU | PTBP1 | 0.0098 |
|  | UCUUUCUUUA | RNCMPT00158 | CUUUUUU | CPEB4 | 0.0217 |
|  | UCUUUCUUUA | RNCMPT00012 | CUUUUUU | CPEB2 | 0.0382 |
|  | UCUUUCUUUA | RNCMPT00269 | ACUUUCU | PTBP1 | 0.0767 |
|  | UCUUUCUUUA | RNCMPT00079 | UUUUUUC | U2AF2 | 0.1483 |
|  | UCUUUCUUUA | RNCMPT00274 | UUUUUUU | HuR | 0.1777 |
|  | UCUUUCUUUA | RNCMPT00044 | CCUUCCC | PCBP2 | 0.4172 |
|  | UCUUUCUUUA | RNCMPT00165 | UUUUUUC | TIA1 | 0.4535 |
| filter101 | UUCCCCUCUC | RNCMPT00268 | CUUUUCU | PTBP1 | 0.0531 |
| filter104 | CUCAUCUUGU | RNCMPT00037 | AAUCUUG | MATR3 | 0.0158 |
| filter130 | CUACCCCUCC | RNCMPT00044 | CCUUCCC | PCBP2 | 0.039 |
| filter142 | CCCUCACACG | RNCMPT00044 | CCUUCCC | PCBP2 | 0.0404 |
| filter162 | GACCCAUCCA | RNCMPT00026 | CCAACCC | HNRNPK | 0.0303 |
| filter168 | AAAAAACAAA | RNCMPT00033 | ACAAACA | IGF2BP2 | 0.0082 |
|  | AAAAAACAAA | RNCMPT00172 | ACAAACA | IGF2BP3 | 0.0392 |
| filter174 | AUGUUGGUGU | RNCMPT00177 | GUAGUGU | SFPQ | 0.0407 |
| filter177 | UAGUUUCUCG | RNCMPT00269 | ACUUUCU | PTBP1 | 0.0569 |
| filter188 | UAUCUUUUUA | RNCMPT00158 | CUUUUUU | CPEB4 | 0.098 |
|  | UAUCUUUUUA | RNCMPT00167 | AUUUUUU | HNRNPCL1 | 0.1743 |
|  | UAUCUUUUUA | RNCMPT00274 | UUUUUUU | HuR | 0.1901 |
|  | UAUCUUUUUA | RNCMPT00025 | AUUUUUU | HNRNPC | 0.2069 |
| filter202 | UCCAUAACUG | RNCMPT00001 | AUAAUUG | A1CF | 0.021 |
| filter203 | GGACAAGCCA | RNCMPT00016 | GGACAAG | FMR1 | 0.0169 |
|  | GGACAAGCCA | RNCMPT00020 | GGACGGG | FXR2 | 0.0704 |
| filter209 | AACAAACAGG | RNCMPT00033 | ACAAACA | IGF2BP2 | 0.0152 |
|  | AACAAACAGG | RNCMPT00172 | ACAAACA | IGF2BP3 | 0.0182 |
|  | AACAAACAGG | RNCMPT00047 | ACUAACA | QKI | 0.0847 |
|  | AACAAACAGG | RNCMPT00178 | ACACACA | HNRPLL | 0.1455 |
|  | AACAAACAGG | RNCMPT00027 | ACACACA | HNRNPL | 0.1882 |
| filter226 | ACUAGAAAAU | RNCMPT00171 | AGAAAAU | PABPC5 | 0.0195 |

**Table S5.** Motifs found by CircCNN from input1data for Mouse back-splicing prediction match the known mouse RNA motifs in the RNA/Ray2013_rbp_Mus_musculus.meme

| FilterID | Motif found by CircCNN | Known motif in database | Known motif sequence | Gene Annotation | E-value |
| --- | --- | --- | --- | --- | --- |
| filter0 | AACAUUUUCC | RNCMPT00239 | CCUUUCCC | PCBP1 | 0.0072 |
|  | AACAUUUUCC | RNCMPT00215 | CUUUCCCU | PCBP3 | 0.0555 |
| filter1 | UAAUAGAAAA | RNCMPT00062 | UAAAAGG | KHDRBS1 | 0.0041 |
| filter10 | AAAAGAAAAU | RNCMPT00062 | UAAAAGG | KHDRBS1 | 0.0007 |
| filter13 | CAGAGAAAAA | RNCMPT00062 | UAAAAGG | KHDRBS1 | 0.021 |
| filter28 | GAAAAAAAAC | RNCMPT00062 | UAAAAGG | KHDRBS1 | 0.0067 |
| filter41 | ACAAUUCCCG | RNCMPT00239 | CCUUUCCC | PCBP1 | 0.0498 |
|  | ACAAUUCCCG | RNCMPT00215 | CUUUCCCU | PCBP3 | 0.0956 |
| filter62 | AAUAAAAGAU | RNCMPT00062 | UAAAAGG | KHDRBS1 | 0.0009 |
| filter68 | AAAAGAGGUC | RNCMPT00062 | UAAAAGG | KHDRBS1 | 0.0206 |
| filter85 | AGAAGGUCCA | RNCMPT00062 | UAAAAGG | KHDRBS1 | 0.0453 |
| filter91 | AAACGUAAGU | RNCMPT00062 | UAAAAGG | KHDRBS1 | 0.0129 |
|  | AAACGUAAGU | RNCMPT00051 | GUGUGUG | RBM38 | 0.0606 |
| filter94 | CUCUAGACCC | RNCMPT00062 | UAAAAGG | KHDRBS1 | 0.0436 |
| filter113 | GACUAAAACG | RNCMPT00062 | UAAAAGG | KHDRBS1 | 0.0081 |
| filter120 | AAAGAUAACG | RNCMPT00062 | UAAAAGG | KHDRBS1 | 0.0215 |
| filter140 | AUUCCAUUCC | RNCMPT00239 | CCUUUCCC | PCBP1 | 0.0394 |
| filter147 | CUUUUCGAUU | RNCMPT00215 | CUUUCCCU | PCBP3 | 0.0103 |
|  | CUUUUCGAUU | RNCMPT00239 | CCUUUCCC | PCBP1 | 0.0792 |
| filter150 | UUUAAAUACG | RNCMPT00062 | UAAAAGG | KHDRBS1 | 0.0384 |
| filter152 | GCAAAAUGGA | RNCMPT00062 | UAAAAGG | KHDRBS1 | 0.0015 |
| filter153 | CUCACAAGGG | RNCMPT00062 | UAAAAGG | KHDRBS1 | 0.0014 |
| filter156 | UGGGUAUGCC | RNCMPT00051 | GUGUGUG | RBM38 | 0.0181 |
| filter160 | CUGGUAUGUU | RNCMPT00051 | GUGUGUG | RBM38 | 0.0017 |
| filter164 | GAAAAAAGAA | RNCMPT00062 | UAAAAGG | KHDRBS1 | 0.0095 |
| filter167 | CCAUUCAUCU | RNCMPT00239 | CCUUUCCC | PCBP1 | 0.0318 |
| filter178 | ACAGUGUAGU | RNCMPT00051 | GUGUGUG | RBM38 | 0.0461 |
| filter181 | GGUGUGCGUC | RNCMPT00051 | GUGUGUG | RBM38 | 0.0258 |
| filter185 | GAAAAAUCGA | RNCMPT00062 | UAAAAGG | KHDRBS1 | 0.0325 |
| filter186 | AAAGAAUAAA | RNCMPT00062 | UAAAAGG | KHDRBS1 | 0.0224 |
| filter189 | AUCUAAGGGU | RNCMPT00062 | UAAAAGG | KHDRBS1 | 0.0126 |
| filter198 | AUAAAAGAAA | RNCMPT00062 | UAAAAGG | KHDRBS1 | 0.0041 |
| filter201 | CGUAAGAGGU | RNCMPT00062 | UAAAAGG | KHDRBS1 | 0.0065 |
| filter202 | ACUGAAAAAG | RNCMPT00062 | UAAAAGG | KHDRBS1 | 0.0167 |
| filter224 | UAAGGUGUUA | RNCMPT00051 | GUGUGUG | RBM38 | 0.06 |
|  | UAAGGUGUUA | RNCMPT00062 | UAAAAGG | KHDRBS1 | 0.0691 |
| filter240 | AAAAAAAGUC | RNCMPT00062 | UAAAAGG | KHDRBS1 | 0.0492 |

**Table S6.** Motifs found by CircCNN from input2 data for Mouse back-splicing prediction match the known mouse RNA motifs in the RNA/Ray2013_rbp_Mus_musculus.meme

| FilterID | Motif found by CircCNN | Known motif in database | Known motif sequence | Gene Annotation | E-value |
| --- | --- | --- | --- | --- | --- |
| filter1 | AAAAAAAAAA | RNCMPT00062 | UAAAAGG | KHDRBS1 | 0.0189 |
| filter37 | AAAAAAACAA | RNCMPT00062 | UAAAAGG | KHDRBS1 | 0.0333 |
| filter38 | AUAAAAGUGA | RNCMPT00062 | UAAAAGG | KHDRBS1 | 0.0056 |
| filter54 | CAUUAAAGAA | RNCMPT00062 | UAAAAGG | KHDRBS1 | 0.0224 |
| filter116 | AGAAAAAGUU | RNCMPT00062 | UAAAAGG | KHDRBS1 | 0.033 |
| filter117 | CAUAAAACGA | RNCMPT00062 | UAAAAGG | KHDRBS1 | 0.0184 |
| filter119 | GAAAAGAAAG | RNCMPT00062 | UAAAAGG | KHDRBS1 | 0.0242 |
| filter122 | UACCUAAAAG | RNCMPT00062 | UAAAAGG | KHDRBS1 | 0.0006 |
| filter125 | UUCCCUGUGA | RNCMPT00215 | CUUUCCCU | PCBP3 | 0.0452 |
| filter126 | AAAAAAAAAA | RNCMPT00062 | UAAAAGG | KHDRBS1 | 0.0173 |
| filter138 | UAAAACAUGA | RNCMPT00062 | UAAAAGG | KHDRBS1 | 0.0165 |
| filter160 | UGUAUGAGGA | RNCMPT00051 | GUGUGUG | RBM38 | 0.0673 |
|  | UGUAUGAGGA | RNCMPT00062 | UAAAAGG | KHDRBS1 | 0.0972 |
| filter165 | AGGAAAAAGC | RNCMPT00062 | UAAAAGG | KHDRBS1 | 0.0012 |
| filter190 | GUCGAAGGAC | RNCMPT00062 | UAAAAGG | KHDRBS1 | 0.025 |
| filter192 | AAACAGCAAG | RNCMPT00062 | UAAAAGG | KHDRBS1 | 0.0148 |
| filter208 | CUAAAUUGAA | RNCMPT00062 | UAAAAGG | KHDRBS1 | 0.0102 |
| filter209 | GAUUCAAAGG | RNCMPT00062 | UAAAAGG | KHDRBS1 | 0.0236 |
| filter211 | UUGAAGGUUC | RNCMPT00062 | UAAAAGG | KHDRBS1 | 0.0158 |
| filter238 | CAAUAUCACU | RNCMPT00215 | CUUUCCCU | PCBP3 | 0.0238 |
| filter242 | ACACUGUCCG | RNCMPT00062 | UAAAAGG | KHDRBS1 | 0.0224 |
| filter245 | AAAAAAAAUC | RNCMPT00062 | UAAAAGG | KHDRBS1 | 0.0134 |
| filter254 | CAUAAAAGUC | RNCMPT00062 | UAAAAGG | KHDRBS1 | 0.0102 |

**Table S7.** Motifs found by CircCNN from input1 data for fruit fly back-splicing prediction match the known fruit fly RNA motifs in the RNA/Ray2013_rbp_Drosophila_melanogaster.meme

| FilterID | Motif found by CircCNN | Known motif in database | Known motif sequence | Gene Annotation | E-value |
| --- | --- | --- | --- | --- | --- |
| filter15 | AUGUCCAUUC | RNCMPT00123 | GUGCAUGC | A2BP1 | 0.0499 |
| filter22 | UUUAACUAAA | RNCMPT00147 | AACUAAG | CG2931 | 0.023 |
| filter32 | GUUGGGUUUA | RNCMPT00120 | UUUAGUU | FNE | 0.0536 |
|  | GUUGGGUUUA | RNCMPT00132 | UUUGGUU | RBP9 | 0.0914 |
| filter40 | AUCAACGAGA | RNCMPT00007 | CAACGAA | CG2950 | 0.0331 |
| filter54 | GAGAGAGAAG | RNCMPT00060 | AGAGAAG | RNP4F | 0.0252 |
| filter110 | GCAUAUUUAA | RNCMPT00148 | CCAUUUU | ROX8 | 0.0496 |
| filter117 | UUUUUCCAAU | RNCMPT00080 | UUUUUUC | U2AF50 | 0.0305 |
| filter126 | AAUAGUUUGA | RNCMPT00010 | AUACUUUG | CG5213 | 0.028 |
| filter130 | AGCUGUGUCU | RNCMPT00270 | UGUGUGU | ARET | 0.0168 |
|  | AGCUGUGUCU | RNCMPT00003 | UGUGUGU | ARET | 0.0496 |
|  | AGCUGUGUCU | RNCMPT00011 | UGUGUGU | PAPI | 0.0496 |
|  | AGCUGUGUCU | RNCMPT00114 | UGUGUGUU | ARET | 0.1325 |
| filter139 | GGGGCGCACG | RNCMPT00124 | CCGCGCGG | LARK | 0.0154 |
|  | GGGGCGCACG | RNCMPT00097 | UCGCGCG | LARK | 0.0478 |
|  | GGGGCGCACG | RNCMPT00035 | UCGCGCG | LARK | 0.0888 |
| filter147 | UAAGUAAGUU | RNCMPT00147 | AACUAAG | CG2931 | 0.0393 |
| filter181 | AGGUUCAUGC | RNCMPT00123 | GUGCAUGC | A2BP1 | 0.0464 |
| filter185 | GGGGUGGGGU | RNCMPT00094 | GGUAGGG | HRB98DE | 0.0509 |
|  | GGGGUGGGGU | RNCMPT00095 | GGUAGGG | HRB98DE | 0.0715 |
|  | GGGGUGGGGU | RNCMPT00096 | GGUAGGG | HRB98DE | 0.0715 |
|  | GGGGUGGGGU | RNCMPT00029 | GGUAGGG | HRB87F | 0.0795 |
| filter197 | GUGUGCAUCC | RNCMPT00123 | GUGCAUGC | A2BP1 | 0.016 |

**Table S8.** Motifs found by CircCNN from input2 data for fruit fly back-splicing prediction match the known fruit fly RNA motifs in the RNA/Ray2013_rbp_Drosophila_melanogaster.meme

| FilterID | Motif found by CircCNN | Known motif in database | Known motif sequence | Gene Annotation | E-value |
| --- | --- | --- | --- | --- | --- |
| filter10 | GCACUGCACU | RNCMPT00145 | AUUGCACA | SNF | 0.0465 |
| filter19 | UUACCACACG | RNCMPT00124 | CCGCGCGG | LARK | 0.0375 |
| filter46 | UAAUAAACUU | RNCMPT00142 | AUAAUAA | QKR58E-1 | 0.0377 |
| filter59 | GGAGGGGCUC | RNCMPT00134 | GGAGGGG | B52 | 0.0001 |
| filter66 | ACACGGACCG | RNCMPT00015 | ACGGACA | FMR1 | 0.0267 |
| filter84 | AAGUCGUGCG | RNCMPT00035 | UCGCGCG | LARK | 0.0227 |
|  | AAGUCGUGCG | RNCMPT00097 | UCGCGCG | LARK | 0.0479 |
| filter94 | CCCCACGAAG | RNCMPT00124 | CCGCGCGG | LARK | 0.0174 |
| filter129 | UCCACACACA | RNCMPT00069 | ACACACA | SM | 4.26E-05 |
|  | UCCACACACA | RNCMPT00008 | ACACAUA | CNOT4 | 0.0346 |
| filter139 | UGGGCCAUUC | RNCMPT00148 | CCAUUUU | ROX8 | 0.0355 |
| filter174 | ACCCUAUUAA | RNCMPT00137 | AACCCUA | MUB | 0.026 |
|  | ACCCUAUUAA | RNCMPT00148 | CCAUUUU | ROX8 | 0.0593 |
| filter182 | UAUUUAAUUU | RNCMPT00174 | UAUAUUA | SHEP | 0.0365 |
| filter183 | GGUGGUGUAA | RNCMPT00122 | GUGGUGU | BRU-3 | 0.006 |
| filter201 | AGCCCUCGAC | RNCMPT00007 | CAACGAA | CG2950 | 0.0486 |
| filter223 | ACACACGGAG | RNCMPT00069 | ACACACA | SM | 0.0123 |
| filter224 | CAUACAUUUA | RNCMPT00175 | AUAUUUA | SHEP | 0.0227 |
|  | CAUACAUUUA | RNCMPT00068 | AUAUUAA | SHEP | 0.0977 |
| filter240 | UUUAAAUUAU | RNCMPT00105 | UGUAAUU | PUM | 0.0829 |
|  | UUUAAAUUAU | RNCMPT00174 | UAUAUUA | SHEP | 0.0829 |

**Table S9.** Motifs found by CircCNN from three datasets: human, mouse, and fruit fly.

|  | Human | | Mouse | | Fruit Fly | |
| --- | --- | --- | --- | --- | --- | --- |
|  | Input1 | Input2 | Input1 | Input2 | Input1 | Input2 |
| filter0 | GAUUGGAGGA | UAAUAAAAAC | AACAUUUUCC | ACCGUCCACA | AUCUAUCUGC | UUCGUGCUCC |
| filter1 | AGCACCAUGA | CAUUCAUCGG | UAAUAGAAAA | AAAAAAAAAA | UAAAAAAGAC | AGGGUCGAUA |
| filter2 | CCUACAAAAA | CUUGGUUUCC | UAUACAGACC | UUUAAGUAUU | CGCUUAUCAA | AUAGUUACAA |
| filter3 | UUCAGACCUU | AGAUCUGGAA | UACCGUCUCU | GUCCGGAAGG | CACAACAAUU | ACCGGCGUUU |
| filter4 | AAGACCAGCU | UCCAUCUUCU | GGGGGUCCAA | GGGUCGAAGC | CGUUUAACAC | CUUCCUUUAA |
| filter5 | CAUUAAGACA | AUUUCGAUUC | UCCAGCAAUU | AAAAGUCUAA | GAGUGGCUCC | UUGGCUCCAC |
| filter6 | AAGAAAGUCC | CAUAUUUUUA | AAUUUUUUAU | UCUCAGGAAC | CCUUUGUCAU | CGCGGCUGUC |
| filter7 | UUAAUCCACU | UUCAGAUUCG | UACGUCUUCC | AAACAAAGAC | UCCCCUUCGA | AGGGUUUUGC |
| filter8 | CAAGUUUAUA | CCGAUAUCAA | AAGUAUUGUU | GCAUUACUAU | UCUAUUAUAU | UUUGUAUAUA |
| filter9 | GAGAAAGUUA | CACCUAGGAG | AUUUUACGAA | GUCUCCAAUU | CAGUUAGACU | AUAAAUAUAU |
| filter10 | CACCUACUUU | UCUCCAUUCA | AAAAGAAAAU | AUUCUCUCUA | ACCAAUAUAU | GCACUGCACU |
| filter11 | ACAUAAGAUC | CUCGUUCUCA | UCCAAUCUAU | CCUCGAGUCU | UACCACUCAA | GCUGCUAACC |
| filter12 | AAGUUCUGAC | UGCGACAGAC | UAAUGUCCGU | CACACUCAGG | AGUGGACGUC | CCAACUCCAA |
| filter13 | CAGUGGUUUA | CUUCGAAACC | CAGAGAAAAA | UGAGGAAUCC | GCCUAUCCCC | AGGUCCAUUC |
| filter14 | UGUUUAAACG | UCUUUACACC | GAUCCAUUCG | UAGUUGUAGC | GGCUAGCGAA | ACAGACCUCA |
| filter15 | AUCCGUCUUC | AGUACCUUAC | UAAGUUGGGU | GUUCGAUUUC | AUGUCCAUUC | GCGGGCUGCG |
| filter16 | AUUUAUUUUA | UUAGGACAGU | AAAUUUUAAA | UUCACCCACA | ACGUCGAGUU | CGCAGUCAAA |
| filter17 | GCAUUUUAAG | UUAAUUAAAU | CACGAAGUAU | CACGUCGAAG | AAAGGCCUAA | UGUAUCAACA |
| filter18 | CAAUAACCUG | CCAUUUUCUU | UAUCCUGACC | GAAUGGAACG | CACGCAGGGA | AAGCAGGAAC |
| filter19 | CGUCUUUCUU | GAAUACUUAG | CUCCUCAUAC | UAUACUUCGA | GGUCAUUCAU | UUACCACACG |
| filter20 | CAUGUGUUAC | AACUUCCUCC | GGUUUUACGG | ACAAAUACAC | CGCCGAGCCU | UAUCGGGUUG |
| filter21 | CAAUACUAGC | UGGAACUUUU | AAACCCCUGC | GACAAAAACA | CUACUCACAG | UGCGAUCCAG |
| filter22 | AAGUAUCGAU | UGUGUUAUAA | CACCCCCAGU | UACUUAGUCA | UUUAACUAAA | UGUUAAAUUA |
| filter23 | ACUAGUGUAA | GUACAGUCUU | UGUUACUACG | AGACAUGACA | GGGGUAAGUU | CAUACCUAGC |
| filter24 | GUUGAUUCAA | ACCAUAUUUG | ACAUUAGGGG | ACACGUUCUG | CAUCCGACGG | UUACGAUCUG |
| filter25 | CUUGACGUUA | AGAGUUCCAU | CAUGCGUCUA | ACCACAACAA | UCACAUACUC | CACCUCUACU |
| filter26 | UACUGGGUAA | GCGGCUAUCA | GAAGUAAUGG | CUUCACCCAG | AAGAAAGGAA | CGUCACCUCC |
| filter27 | AGAGGUCGAA | UUAAUACGAA | GUUUAGGACU | GGUCCGGGUC | AGAAUGACCA | GUAGUCAACU |
| filter28 | CUGAAUAGAA | UUUUUUCCGA | GAAAAAAAAC | CAGAUACACA | UUGUCUAUUC | UUAUCCAUGU |
| filter29 | CGUCUCGAAA | GGUCGUACUU | UUAAUACUAG | CCGGUGGUCC | GGUAACUAUA | AACCUCGAGC |
| filter30 | UUUCCCGUAC | ACUCAAUUAA | CGUCAUCUGA | AUGGUCACCA | GCUACACCCC | CCUGCUACAU |
| filter31 | AGUCUCGUAU | GACAACGCGG | CCAUAAUGGG | AACGAGAGUU | AGGCAGACGU | AUUCUCUCGA |
| filter32 | UGGGCCUUAC | GUUUAUCUAG | GAUCCAUUCA | CAAUGAGCAG | GUUGGGUUUA | AGCGAAAAAA |
| filter33 | AGACUUAAGA | UCAGAGUUCC | AAAGAGAGAC | ACAUUGAUCA | UGUGGCGUUU | CGCCUCCUUC |
| filter34 | UACAUCCAAA | AGACUUUUUC | CGUCCGAUGU | UCCUGACGAU | GUACGAAGAC | UCCAUUCAUA |
| filter35 | AAAAGAGAAA | UAUCAAAGAG | UCAGCAUAAC | GCUGGGGCCC | AGUGGUUUAC | AGAGCGUGAG |
| filter36 | UCUCUUUUUG | AAUUUCCAUU | AGUCUGCAAU | AACCUGUCCG | AUGUAAUCGU | UCCAAUGGUU |
| filter37 | CUUGACUUGA | AACGAAUGAC | GCCAAAGUUU | AAAAAAACAA | UUAACUAGUU | AUUCCGUUAA |
| filter38 | UCCACGUCCU | AUUAAUUGAC | AAGACACCCU | AUAAAAGUGA | UUAUUCCGCG | AGAAUCAUCA |
| filter39 | AAAAAUCGUU | GGAAGAAGAA | AUCACUCUUU | UAUACAUCCA | UGUAACCAUA | GUGCUUAUUU |
| filter40 | UCGGUCGGAC | UCCAUUCGCG | GUCAUGGUCC | UUAAUACACU | AUCAACGAGA | UGCCAACGAU |
| filter41 | UCACUUAGUC | CCCUGUCUGU | ACAAUUCCCG | AUAGAUACUA | UCCUACUUAG | CCUUUGAGCA |
| filter42 | AUGUCAUUUU | ACCUUCCACA | UAGUCAAUCC | AGAGGAGAAA | AUCGGCCUUA | AGAUAAUACC |
| filter43 | AAAAUUUUAC | CAUCCGAAGG | ACUACGCCAU | GUCGAAGUCU | GCAUGGUGUU | CUAAGCGAUA |
| filter44 | UAUCCCAUUU | CCGCCAUGUC | AUACAAUUCA | GUGAAGUAGA | AAGUUGUAGG | GGAUGAGUAA |
| filter45 | GCUUUUCUCA | GAAAAACUUA | CGAGGACAAA | UUCUCAUGCA | AAGGGAAAAA | ACGAACGUCC |
| filter46 | UACACAACAG | UUAUGAGGAA | GUUCCUGACU | GGGACAAAUC | UACACGUGUU | UAAUAAACUU |
| filter47 | AACUUUUUAU | CCGUACCAAC | AUCUAGUGGA | GUUAUUCUAU | AUGUGACACU | CAUUCAGGAA |
| filter48 | AAUCGUCAAG | UCUUACCCUU | GAGCACCUGC | ACUCGUUCUG | GUGCCAUCCA | ACCGGAGGAG |
| filter49 | UUUUCUCAGG | GGUGUUCUAU | CAUUUUCUGU | UUGAACUAGA | AUAGGAGAAU | GGCUAAGGAC |
| filter50 | CGUCCUAGAU | AAUGAAUGGU | CACUAACCCC | CGAAGACGUC | UCCGUCCAUU | CUAUCUCGCC |
| filter51 | GUUAACUACA | UGGAGUAAUU | CGUCUAUAGA | AAAGUGUCGC | GAUAAAACAU | UGCCUGGGCG |
| filter52 | GACACUAUGU | UAACCGUUAC | CUCCUACUCC | GAGGUGUCAG | AGUUGCCACU | ACAUAAAAAA |
| filter53 | AAAAUUUACA | AUAUAUUGAA | UACCACUUGA | AUCACUAGCU | AUCCUAUACC | UUUACCAAAA |
| filter54 | UAAUCUCUGC | AGAAGGUUUA | CUAUUAACAA | CAUUAAAGAA | GAGAGAGAAG | UCGGCUUCAA |
| filter55 | ACUACAGAUA | CUGUGAAGUC | GUCUUUCAUU | CCCUUGCAUA | GUUUGUCUGC | GUCAUUGAUA |
| filter56 | GAAGCCUCCU | UAUCAAAAAA | ACAAAACUGA | UUAAAAACGC | UGAUAUAUAU | UAAAGUAUUA |
| filter57 | UUAAUUUAGU | CAAUUACAUA | CACCAUUCAU | AUCAGUGAUG | CCACGUCCAU | AUUAUUACAA |
| filter58 | UCAUAUUACC | GCACUUAUCG | GCUCCAAACC | AGGGACAGUC | UGACAUUGUU | AAACUUCACA |
| filter59 | CUAAAGAGAG | AUCCACCCGU | AGACUCCUUC | GUCCAAUACC | UGAAUAUGUA | GGAGGGGCUC |
| filter60 | CUGAUAGUAA | ACAUUCACAC | UACAUGUCUA | AUCCAUAUAG | GCAUUACUUA | AUAAAACGUC |
| filter61 | GGGGUUUAAU | CCAUUCUCCG | CCCUUUUGGU | CUUACUUAUC | AUACUAUAAG | AAGUGAAAGA |
| filter62 | UUAUUGACUU | CCCUCAAAUA | AAUAAAAGAU | CAUUCAUUCA | AAAAUAGUCU | AGUAUAGGCG |
| filter63 | GAAAAAAAAA | ACGACUCCUG | GGUCCGUAAG | UUUUUACGAA | AAACAAAGGG | UCAAUAACCG |
| filter64 | UUAUAUACAA | UCCAUACUCA | UAAUUGUUCC | AAUACGAUUG | ACCUGCUAUA | UCAACGUCUC |
| filter65 | UCUAAAAACA | AUGAAGUGUG | UAUACUUCCC | UAUACUAAAG | UUCUAAGAUC | AGGAAAGGGU |
| filter66 | UGUAGUCGAU | AACACUCAAC | AUAACGGUGU | CCACAAUUUG | UCACUGCUAC | ACACGGACCG |
| filter67 | AACGAUAACG | CUAUGAAAAU | GUACCCCCUA | ACCUAGAUGA | GUAGUCUCGC | UGUGAAAUUA |
| filter68 | UCACAAGUAA | UCUCUAACGG | AAAAGAGGUC | GACAAUCAUU | UAAAAAUAAC | GACUCGGUGG |
| filter69 | GUCUCUCCGA | UAAUGAGAGA | CAGUGGCUAA | AUUCGGCAUC | UACGGGAACG | UCGCAUUUUG |
| filter70 | UUCCUUUUUU | UUUCUAUAUU | AAGGUCAUCC | AAUACCGACA | UCUAUUGCUA | AAGGCAUCCC |
| filter71 | ACGGUGUCCU | GAAGACCCUU | GACACAAGAG | CACUUUUAAU | UAAAAGGUUU | UCUAAAAGAU |
| filter72 | AAAAAAAUCU | AUAAGUAGAC | CUCCAUCCAC | GAGGUCCACU | CGUGAAAUUC | AGACGGUGUU |
| filter73 | UACAGACCGG | UCUUUCUUUA | AGAGACCCGA | AAUCUGCAAG | UGAAAUACCC | GUCCACUCAC |
| filter74 | AUAUAGACGU | CAGAGACUAG | UACAAAGGUC | AGAACAAAGU | CGCUGUCAUA | CAUUCACAGU |
| filter75 | CGUGCCCCCC | CAUCCAUUCU | UCACUCAUUA | CUAGUGCUAC | AAGUACCGUU | AGAAGAGUGA |
| filter76 | AAAAAAAGUC | ACAAUCAAAU | UCUCAGGCUA | GGAACUUCUG | UUAGCGGUUU | AACGUUUCGA |
| filter77 | GAGAAAACUU | UCCACACAUC | CAAUAAUUAA | GAAUUUUCUU | UUAGCGCAAU | CCUUGAAGGG |
| filter78 | UCAACGGUUG | UCUUAAACAC | UUGAACACAC | CCCUACCGUU | ACGGCGGAAU | CUCGUUACCG |
| filter79 | AAACGUAUCA | UGGUCGACCA | CUCUGUUCAG | GGAAUCCCUC | CAUCAAAGGU | CUUUACCUAA |
| filter80 | GGCAGUUAAA | UCACUUCUGA | UGUUGCGGAC | ACCCGCCCCC | GUAUUUUGAG | GCUAAAACUC |
| filter81 | AAGGAAACCA | UCACGUCACA | GUUAGCCCGG | ACAAAAGUCU | AAGAUGUAAA | AGAUAUGUCC |
| filter82 | GUUAUUCCGG | UCACAGUCAG | AGACAUCUCC | GGUCCCGAUG | UGUCUUUGGU | CGAAGCUCUG |
| filter83 | AGAUAAACUA | CAAAAUGACU | CCGGGCAAAG | ACACACACCU | UCGUCAUUCG | AGCGUCCAUU |
| filter84 | UUAGUUUUUU | UGUGUACCUA | AUUCAACCUG | UUCCACUGCA | GUCCUGGCCU | AAGUCGUGCG |
| filter85 | ACGUCUUACU | ACUGUAAUGU | AGAAGGUCCA | AUUACUCCAA | CUCGCAUGAA | CCAUGCAGGA |
| filter86 | GUUUGUGUGA | UUUUAAAAAA | AUAUCUUGGU | GGUAAUCUCG | UACGGGGGCA | GCGCGGGCUC |
| filter87 | GAGGUGUCGU | GGAUGCAGUU | UGACCUCGGA | CGAUUCCAGA | AUAACUAUAA | CCGAUGUCCG |
| filter88 | CGAACUGUCA | CAAUAUUGUC | GGCUUCUUUC | AGUUUUUUUC | AAAACCAAUA | UGCGUUAUUG |
| filter89 | CCGAGUAAAG | CAAUGACAUU | GGGACUUCCG | AGAGAAACAA | UGAGGAACAA | CCAGUCAGGU |
| filter90 | UCCAGGGACC | AGAACAGUCA | GGAAUUUCUC | CGAGUCUGUC | GGUGCGUGAC | GUCCAUUCUA |
| filter91 | GACAAGGGGG | CCAUGUACUA | AAACGUAAGU | GAGAUACCGG | AUCCCUAGCG | UAGAAGACAA |
| filter92 | GAGGUCUCUC | AAGUGACCAC | CAGACUCGAG | CUUACAGGAG | AUUUAGAAUU | GUCGUUUUUA |
| filter93 | GUUAAGGUUG | GAGUACCAUU | AGAACAAAAU | AAAGACCAGA | UUCAAACGCG | ACUUGCUUAG |
| filter94 | AGAUCGUAUA | GGACAAAGAU | CUCUAGACCC | UUACCAAAUU | UCCAUUCAUU | CCCCACGAAG |
| filter95 | AUUUGUAACA | CCCCGGGGGA | UGUCGCCACA | GUCCACUUCU | GGUCCACUCA | CUAACUGGUU |
| filter96 | AAAUGUCUCG | CAAACUCAAU | UACUUUUUAA | ACCCCCGUGA | UACCAGUAAG | UAAGGAUAAG |
| filter97 | AGUUAAGAUA | UGAACCUCUC | UGAGUAUGUA | GGAGGUACGU | UGAUUUCUGG | ACCCAACAGA |
| filter98 | AACCGCACCC | UCGAGAUUCC | GGAAUGUCCA | AGGUCUUAUA | GGAACGUUUC | ACGUCGCCUC |
| filter99 | ACUUCACUUC | AGAUCCCCAU | AACAUCCAGG | UCCAUUCAAU | UGCUAAAUUA | AUAUAAGCUG |
| filter100 | GGUGGGACGA | AAGGUGAGAA | CCGUGAUAAA | CCACAACCGA | UACUCAUUUU | CUUGCGGUGA |
| filter101 | GUGAGGGGAA | UUCCCCUCUC | ACUUUUGGGA | CUGAACUGAC | CCGGGGUCUU | GUCAUGCAGU |
| filter102 | UGUUUCUUCA | AGAGACGACU | CUGUAAAGAU | AGAAUCCGAG | CGCCUCCAUU | ACAAAGAACG |
| filter103 | AUAUGAGGAA | UACGGACAGG | AGUGCGGUGA | UUUUGAUCAC | AUAUAAAAUC | GACUGACGCA |
| filter104 | AUUAGGGGUU | CUCAUCUUGU | UGUUUGACAG | AGAUCAGAGA | AGACCGUCUU | AGAAAAGUCU |
| filter105 | UAAUUAAGAA | GAGUCCAUCC | AAGAUAAGUC | UAUGAUAUUA | UAAGAGAGAU | CUCUACUACU |
| filter106 | UUACGUUACA | UGUUGUAGGG | CAUUCGUUAC | CAUUCAUCGC | UUGUUGGCUU | UUGCAGUAAA |
| filter107 | ACAAAAACCC | AAGUUUUCUA | AUCCACAAGU | AUCUCCAUGA | CGUCUGUGAA | ACACCUCGCG |
| filter108 | AGUACCUGGU | CACUCAGCGU | GGCCUCUCUC | UCGUAGUAGG | UGGACUCGAG | GUUAGCAGCG |
| filter109 | GGUGGGGGGG | GCCUUUACAG | ACGUCCAUGA | UUAACUCGUC | AUUGUGACUG | AAUGUGUCUC |
| filter110 | CUGGAAAAGU | AACGUACCCG | AAAAUAACCU | AGUUACCCCU | GCAUAUUUAA | AUUUUUAGUG |
| filter111 | UAGUAAAUAC | CCUCCGUCCA | AAAGCCACAC | UCUCAACUUC | CGACGGGUAG | UCUUUCCAAG |
| filter112 | GAGGGAUGUC | CAUCCAAUCA | ACUUUAACAA | UAUUUAUUUU | AAAGAAUUAG | UUUAAUUUUA |
| filter113 | ACAUCAAAAG | UGGUCCAUUC | GACUAAAACG | UCCAGUAUCG | GUCGUCACAG | AGAAAGGCCU |
| filter114 | CCUCUACCAA | ACGGUUCAUG | CUCCAAUAGA | AGUGUUGACA | UACCACAGUG | AGUAUAUGUG |
| filter115 | GAAUUAGAGG | UCCAGUCCAA | UUACCAUACA | CUAUUCGGUC | GUGGAGCGGC | AUGAUAAUGU |
| filter116 | AAAGUUAAUC | UCCAUAUCUC | GACCACAAUA | AGAAAAAGUU | UCAACGUGUC | UGUCAUAUAU |
| filter117 | GAAUCAAUUA | GGGAGCGUAU | UAGACUGUCU | CAUAAAACGA | UUUUUCCAAU | AGUCCAUUAU |
| filter118 | ACUUUCUCAC | CUCGAUCGAU | UGUUCCCUAC | AACUCAAAGU | UCUGUCUCAU | AACGUCAACG |
| filter119 | AUUUAAAUUU | UCGUAAUGUU | ACACUUGAUA | GAAAAGAAAG | AAAGGGAAAU | GCGUUUAUAC |
| filter120 | GGUCCAUAGG | AAAAAUUCCA | AAAGAUAACG | GAUGUCUCCA | GCGAUGGCCA | AUAAACGUCA |
| filter121 | AAUUCAGAUC | UCCACUAUUU | AAACGCUCGA | UCCGAAGACA | AAGUAAUCAC | CGCCUGCAUU |
| filter122 | AGAGUUACUC | UCAAUCAUUA | GACGCUCUCG | UACCUAAAAG | UUGGGCGAUA | UUUAUGCGCA |
| filter123 | UACGAUCCUG | UGCUGUGUCG | AGUGGUUGCU | AGGUCGUCUA | UCAAUGGAAG | AAAGAGAGAG |
| filter124 | UCCGACAAAU | ACUCAAAAAG | UAGAAGCGUC | GAGAAGUAAA | GAAAUGUCCC | AGCUAAGAGG |
| filter125 | AUGAUAAGAA | CAGUCCGUAA | AUCCUUCGUC | UUCCCUGUGA | UCAUUCAUGU | UCCUUCCAGG |
| filter126 | AGUUUAUCAC | AGUGUUAAAG | UUGUACCGUU | AAAAAAAAAA | AAUAGUUUGA | CGGGGUCACA |
| filter127 | CCCGAGAGAU | UCAAUCAAAU | UUACAAAUUU | AAGGUGAAUA | UCGCUCUGCC | UUCAUAUUCA |
| filter128 | CUAGGGGCCG | ACGUUAAACA | UCGGUCUUCC | CAAGAACCUA | ACUCAAGUUA | AAACAUUGUU |
| filter129 | UUUCCUCUCA | GUAAAGAAGU | GUACUAUCAC | GUAUUCUCCA | CCAGAGGUUU | UCCACACACA |
| filter130 | ACGAAGUCCA | CUACCCCUCC | GGAACCACGU | CGGGACCCGG | AGCUGUGUCU | CACUUACAUG |
| filter131 | GGAGUUCUCC | AUGGUAUCGG | UCAUUGCUGU | CACCAGCUUA | ACUAAUAAUA | GGUUAGUACG |
| filter132 | AAUGUCAAUC | AAAUAGCGAG | CCACCGAAUC | UCCUUCUAAG | UGCUUUGAUU | UUGUUAUUGU |
| filter133 | CCGAUCUCAC | GAGUAAAGAA | AGUCCAAAUU | AGGACAGAAA | UCACCAGACG | CAGAAUGACG |
| filter134 | ACGACAUUGG | CCUAGGUGUU | GAUGUCGAGG | CACUCACAGA | UUCAUCGAGG | UCUGUUGCCC |
| filter135 | UUGUCUUAGA | UGUAUGGAGU | ACUUGAAUUG | AGUUAAAACA | UAAAAAAAAU | GGUGAAGAUG |
| filter136 | UCUAGAAUUC | UGGUCGAUGU | ACACAAUUGG | GCGGACGCCC | AUCGGUCAAU | AAUAAGGUAU |
| filter137 | AAUAAGGAAA | ACCUCUCAAC | UGUCCACUCA | CUCACGCAUU | UGGCGUGGGU | CUCCGAGCUU |
| filter138 | UACACAAAUA | GGAUGUCGGG | AGUCUCACAU | UAAAACAUGA | CGAGUAGAGC | CUGGCGUAGC |
| filter139 | ACGAACGAAA | GUCCCUUUUC | CUUCAUACGA | GUCCCUGGGG | GGGGCGCACG | UGGGCCAUUC |
| filter140 | CAAAUUCUCC | UUAGUUCGCA | AUUCCAUUCC | UCGCUGGACC | GGUAGAUCGU | CUACUCAACU |
| filter141 | AAAUGUCCGG | AUUAAACCGG | CGUUUUGUCA | GGGGUCGUCA | CAGGCACCUU | UAUUCAGUGU |
| filter142 | UGACGAAAAU | CCCUCACACG | ACACACGAGA | UCGUCAAUAG | AGAGGAAGCG | UUCCAUCCAA |
| filter143 | GUUGUAAUAC | ACAUUCUACU | CACGCGAAGG | UGAGACCCCU | GAGUUCCGGU | UAGGUUGGUA |
| filter144 | GGCUGUUAUC | UAAUUGUCCA | CCAUUCACAA | GACACCUGCA | GUCCGUGCCU | UUAAUUAGCU |
| filter145 | UGUUAAUUUA | AUUGACACAA | ACUAUAAACA | UCGUCGGUCG | ACAACCGAUC | AAGAUGUAAC |
| filter146 | AAAUUACCGG | CUGGUCCACA | UUUAGUUUGG | AGUGUCACUU | UCCGUUGUUA | GGAUAUGCAU |
| filter147 | GUCUCGUAGU | UCAAAUGGAA | CUUUUCGAUU | AAGAAGAACU | UAAGUAAGUU | AGUACCAUUC |
| filter148 | GUAGGUACUU | GGUGUUCCGA | UUGUGAGAGA | GUUUGUCUAU | AGACUAAAGA | UUUAGCGAGG |
| filter149 | AAACAUAUCU | AACCUGUCGG | ACGACCACAG | UCGACAGCCC | GAGCGGGAGG | UUGCUCUUCC |
| filter150 | GACCCUCACU | UACCAGAAUA | UUUAAAUACG | AGGUCAUUCG | AUGUCUGUAG | GUCUAUGUUC |
| filter151 | UUUGAUAACU | UGUUCCAUAC | ACUUAUUUCG | UGUAAUCUUC | AUAGUCUAGA | AAUCAGAGAU |
| filter152 | AAAUAAAAAA | UCGAGUUUUA | GCAAAAUGGA | ACGAUCCAUG | CAGUUGUUAG | UCCCUUGCAA |
| filter153 | UCUAGUAGAG | AUGUUACUGU | CUCACAAGGG | ACUAAUGUAA | AAAAUAAUAA | AUUAACUAAA |
| filter154 | AAUUUUGUGA | UACGCCCAUU | UAUUUCUUCU | AGUAUCCAAU | AUUACAGUAC | UCGAGGCCGG |
| filter155 | GUAUCGAACC | GUUCUGGACG | GACCUGUAAA | AAAAAUUUCU | GUUUCUAUUU | AACGUCGUCC |
| filter156 | GGGACAGAGA | CCACCCAUGU | UGGGUAUGCC | UAUUAUAACC | CACACCGCCA | AGUAUCAUCC |
| filter157 | GUAACUUUCC | GAUCCUUCCU | AAGAAAAUCU | ACGGUACGGU | CUUAGUAAAC | CAGCUCAGGC |
| filter158 | UAAAGAAAAU | UGUCACCCCU | GCCCAAACGU | UAUCUUUCAC | GGGAGGUGAU | GCACAGUCGC |
| filter159 | AUGACAAGUG | AAAUACGCGG | UGGUCUUCGU | CCCAUUCGUA | GUAUUUUUUA | AACAAGGGAG |
| filter160 | GUGUCAACGA | GAUAGAAGUC | CUGGUAUGUU | UGUAUGAGGA | AUUCAAUCAU | GGUAAUACCG |
| filter161 | UGUCCAUAUU | CGUUAUAGAC | CCAAUCGUGA | AUACGUCCAA | UAACCGGGAA | ACUACGCUAC |
| filter162 | UUCCUCAGGG | GACCCAUCCA | UUGUCCAUUC | UACCAUCCAA | UAUUUUUUAC | UAAUGUUAGU |
| filter163 | GUUUCUCUAG | UCCAUUCCAU | AUCAGAACGU | CCGAAAAUCU | CGUCAUUGAA | UCAACCACGA |
| filter164 | UCAAAAAGUA | UACUGUAGUG | GAAAAAAGAA | AAGAGAAAUC | CCUGCAUUUC | GAUUGGUAAA |
| filter165 | UUAUAGUAGU | GUCCAUCCAC | ACCCGGAGUC | AGGAAAAAGC | CACGUGUCUU | CGGACCAGGC |
| filter166 | CACCUCAAAG | UCGGCGACCA | CGAGGACAAG | AAUGACUUGG | AUUUACGGCC | AAAUGUGCCU |
| filter167 | CCCUGGAUUA | CUUAGGACAA | CCAUUCAUCU | AAUGUCACGG | GUCAGUUUUA | UGUCUUGUGC |
| filter168 | CUUGUCUCAG | AAAAAACAAA | CGAAAACGUC | GAGAGAGACG | UAUUCGUUAC | CAGCCAUUGA |
| filter169 | UAGACACACA | UGUUCCAUGU | AAGCGCCAGU | UUAUUUUGGG | AUUACAGCAU | CCAUGUCUGA |
| filter170 | UUAGACGUCG | AUUCCAUUCA | AGAGUAUCUU | GACUGAUUGU | UGAUAAUUAU | AUAUGAAGAC |
| filter171 | ACAGCUAAAC | UCAUUGUCCA | UCCCAAUCUC | GAGGUCUCAA | AGUUCCUGAC | AGAAGUUGGA |
| filter172 | CGUCUUAAAU | GCGGUUAAAG | CACUGCGACA | AUUGAAUUUA | GUUGCGUUCA | AAUACGGAUG |
| filter173 | AUAAAGGACA | GAGAAAGGCG | UAAUGGUUUU | CGGAGUCUCG | GCAUUCAUUA | CUAAUCAAUA |
| filter174 | GUCUAGAAUU | AUGUUGGUGU | UCCAUCCAUU | AGAUAUUUAA | AAAUAAAUUG | ACCCUAUUAA |
| filter175 | CAUCACCACG | AAUAGUUCUA | CAGGUGUUAU | GCCCUUCCGU | UUCUUGUUUA | CGGUUAUGGA |
| filter176 | CACUAACUAA | CUACGGUGAC | UAUACAACAC | CUUUAACCAA | GAAGGUGAGA | ACGAUUCGGG |
| filter177 | CUUUCGACAG | UAGUUUCUCG | AUCGGACCGU | GUUCAGUCAA | CGUUACCUGA | UGAUUACGUA |
| filter178 | AGUAGAAGUC | UUGAUUCCAC | ACAGUGUAGU | AGAUUCUCGA | UAAAUAGUCU | AGGCACGACA |
| filter179 | AAGUUGUCCA | CACCUGCUGA | AGCGGUAUUA | AUGAUUACUA | AAAGCGGUAG | UCCAGAAAUA |
| filter180 | GAAAUUGUAG | ACCCCAUGGG | ACAUAAUAUU | AUUAGAUCAG | UCAUCAUCCC | GCAUUAAUUC |
| filter181 | GAAGGUCGUC | AGAGAACGAG | GGUGUGCGUC | AGGUCACAGG | AGGUUCAUGC | GCCAUGACUG |
| filter182 | CUGGCACUAA | UUCCAUCCGG | ACAGGUACCU | AAGUGGACGG | UCAUAGCCUC | UAUUUAAUUU |
| filter183 | AUCUGAAGAA | ACAGUAACUG | GUCAGCCACU | AGUAUACUGA | GUCCGAUCAG | GGUGGUGUAA |
| filter184 | GAGAAAGAGU | UACCGACGGA | GUCCUCAGUG | GGGUCUACGA | GUCCCUAAGG | GUCACUCAGA |
| filter185 | GAUGGAAACG | GACUAAAGUC | GAAAAAUCGA | CUGUGUCCAA | GGCGUGGGGU | AGCGAAAGGA |
| filter186 | GUAUGCAUAC | UUCGUGACGU | AAAGAAUAAA | UUCUCUACUC | CGUAAGUUUA | UCCAACGUAG |
| filter187 | CAAAGAAUCA | UCAAAAUAGA | GAUUAGUCGA | GUAAUUAUCC | CAUCCAUCCA | UGUUUUUAUU |
| filter188 | AAGACCUUCC | UAUCUUUUUA | UGUAGUGACU | AAUGAAGGAA | UCAACUCAUU | AAGUCUUGUA |
| filter189 | AAAAAAAAAA | CAAUUUAAAA | AUCUAAGGGU | GAGUUAACCG | UCCCAUGAGA | AUAUUAUUCA |
| filter190 | GGACAUCAUG | UAAAGAAGAA | AUCCAAGGAA | GUCGAAGGAC | UAAAUUGCGU | UUAGUUAUGU |
| filter191 | AGUCCGUCGA | CAAGUCUCUG | UAGACAACGA | GAAACGUAUU | AUAUAAAGUC | AUUACACGAU |
| filter192 | AGAUGAAUAA | ACCCAGGUAG | UAUCAGGAUC | AAACAGCAAG | UUAAAGGCUC | GUCACCCACA |
| filter193 | CGGAGUCUUU | UAUCUCCCGA | UAAAACAAAA | UCCAUUCAUC | GGUCCGCGUU | CGGCUAAAAU |
| filter194 | AAAACAAAAG | UUAUACUACG | AUGUAGUAGG | CAAUUGUAAC | CAAGUCGAUU | UCUCCGGAUA |
| filter195 | UUCGGAUUCC | UCGACCAUGU | AGAUCAUUGU | CAGUCGCCUA | GCCUUACGCU | GCCACUCAGA |
| filter196 | AAACUCACAU | AAUCGAGACG | UGACGACAAG | CGACGUUAGG | UCCGGUGGCG | GGUGCUAUUC |
| filter197 | AACCGAACAA | GGGGUACUAG | CUCUCACGAA | UCCGCGGAGU | GUGUGCAUCC | AUAGAAAAGA |
| filter198 | UCCAGAGCCU | GAGGCUAACC | AUAAAAGAAA | CAAGACAACG | UUGGGGGAGC | UCUCGGGGGA |
| filter199 | CGAGGAAACU | CGUACGGUCC | GGAUCGAAAG | UACGAUGUGA | GGGAUUUAGC | GCCGACGUUA |
| filter200 | ACAGGAUUAA | GGAUCUUCUC | CAAAGGCUCU | CGGCCUGUUG | ACCGCCAAUA | AACUUGACCU |
| filter201 | AAAUACAAUA | CAAUCCGUCU | CGUAAGAGGU | GAUGACAUUC | ACGACUAAUU | AGCCCUCGAC |
| filter202 | CGUUUCGACG | UCCAUAACUG | ACUGAAAAAG | CGGACCCUAA | ACGGUUGGGG | UAAAUGGCUU |
| filter203 | AUACAAAUUA | GGACAAGCCA | CAGAAUCCAC | UCACGUACUU | AGUUCCGGUA | AACGAAACGU |
| filter204 | GAGAAAGGAC | CCCAUAAGGC | ACAGUAAGAA | CUCUCCAUCC | GGUGGUAAUU | GAUGUCAGUU |
| filter205 | UUUGUGGACA | UCCGGUCUUA | GAAGUCUACG | UAGUUCCGAA | UUUACGACAU | ACACCCGAGA |
| filter206 | AGUCUAUCUC | CGUAAGUCCA | UGUUAAUGAC | UUCCAGUCAU | UGUGACUGUC | GUCCUUCAGA |
| filter207 | AGUUUAACUU | CCUUUUGUCG | GAGAGCCAAC | GGGUACUUCC | CGGAAAACGC | GUAUGUUAGC |
| filter208 | AAAUCCUACG | GUAGACUCAG | GUCCAAGAAA | CUAAAUUGAA | CGGGGCACUU | CAUUAAAAAU |
| filter209 | CAAAAAAUCC | AACAAACAGG | ACUUCUGGCC | GAUUCAAAGG | GUCCUCUGGU | CUACUACCAC |
| filter210 | ACAAAGAGUA | AUGUGAACAU | CCAUUUCCAU | UUUGGCAAUC | AUGGUUGAUG | CUUCCGCAGC |
| filter211 | CGGUAGUCAC | AAAAACCAUC | ACUAUGGACG | UUGAAGGUUC | UUGCAACAUU | AAAAGACAAC |
| filter212 | GAUUACAGAA | UAACUAGGGA | AACAGGACGA | GUCAUUCUGU | UCAUUCAGUC | GGUGGGCGAG |
| filter213 | AUGACUCCGA | GGUAAGCCCG | CGACGUCCUC | CUUUCAUGCC | UUUGAAGUCC | UGGUAAAGCC |
| filter214 | GUAAUACAUC | UCACCAUAUA | CACGUUCCAA | CCAGUACACC | CCGUCCUUUA | UCGUAGAGGU |
| filter215 | CUUUUAUUUU | AACCACGUUC | CACUCUUGUU | AAAUCACAAA | GGUACCUUCU | GCCUUGCGGA |
| filter216 | CACAUACAGA | GACGUUGUAA | AAAGACAAAA | CAAGGGCUCU | UAGGCCGCGU | GUCAACGCAG |
| filter217 | ACAUCUCACU | ACCUGUCUAA | AUGAUUUAAA | UGAAUGCAAA | CCACUAAGUU | GUUUCCAAUC |
| filter218 | AAGAAAAUAA | CUUUGCGUCC | UCCAGGUAUU | CAGUUCCACU | UUUCGUCGGG | AUGAUUAGUU |
| filter219 | AUGACCUUGC | GUGGCCCAAU | GAAGUACACU | UAGCAGCCAA | GUGCCAUGCA | AAGGGAGUUG |
| filter220 | AACAAGCGAU | UAGUGCUGUA | GGCCGUCAGA | UCAGUAGAGA | AUCGGUAAUU | UUUAUAUGUC |
| filter221 | GUAUUUUCGA | AGUUCCAUUC | UCCACAAUUC | CGUCCACACU | GGCUGGCGCU | UCCUCUACUC |
| filter222 | UGGGGAAUAC | CCUACCGAAG | UAAGUUUUGU | UCAGAGGAUU | AUUAGAUUAG | UUCGACUCAU |
| filter223 | GACAAUACAA | UCUGUACAGU | UAAUACAAUC | UCUCUGACUG | CUUGCUACGG | ACACACGGAG |
| filter224 | UAUACUUUUG | AAAACCUCUA | UAAGGUGUUA | ACCGCGGUGU | AUGGAAAGAA | CAUACAUUUA |
| filter225 | UCGUUCACCC | GACGAGUACG | AUUUCUCGAA | ACGUUACUGA | GCCACUCUUG | AUGCAAUCAA |
| filter226 | AGGAGGGGGA | ACUAGAAAAU | CUUGUGAGGA | UCAAUCAUUG | UAAAAUUGUC | GAGAAUGUCG |
| filter227 | UUAGCCCAGC | AUUAACUUCG | AUCUAAAUGA | UAUACAUACA | AGUCAAAAAC | CACCUAUCGC |
| filter228 | UCUCGUUCGU | UCACCCACGC | CUUCUCGGAA | ACUUAUCCAU | AGUCCCCUUC | ACUUAACCUG |
| filter229 | UUUUAGUUAU | UUGGACCAUU | CACUGUUCAC | GGCUGUCAAG | UAUUAACAAA | AGCCCAUAGC |
| filter230 | ACAUAUAUAA | GACUGAUAAA | UCACAUCCAC | AACACCACUC | GGUAGCUGCU | CCUGUUAUAA |
| filter231 | GUACAUAGAG | AAAAAGAGUC | CGUCUAAGGC | GACGAGUCAU | AAGUAGGUCA | CCAUCCAAAA |
| filter232 | AUAAAGUAUA | GGUCGGCCUG | ACGAUGACCU | UUACUUUUGG | UACUAGUCGU | CUAGUACAGC |
| filter233 | UACUUUACUC | UUCGACGCAA | AACGGACGCU | GAUCGACGUU | AUGCGCAUUU | UUGUUUCGUA |
| filter234 | GUUCAAGAUC | GCCUUAUGGA | AAAAAUUGUC | CAAGAUUUCA | UUACUUAUAA | AAAUCCAACU |
| filter235 | UGUGUCUAGG | UAUUUUCCAU | UGUAAUAAUU | UCAUGUGUAC | CUAGUGAAUU | CGAAGGUCAA |
| filter236 | UUUGUAAAAA | GGUGCGAGAA | AUUGUCGUGA | UUCCCAGGAA | CCCUAGGCCA | AAAUCCAUGC |
| filter237 | UCAAUAGGUG | AAUAUAUAAU | GGACAAAGAA | GAAGUACCCU | CUCCUUCGGA | GUACUUUUUA |
| filter238 | AGAACGUCCG | ACUCAUAAGA | GAGGCAGAAG | CAAUAUCACU | UGAGCAGUCG | GGACUAAAGA |
| filter239 | GGGAGAAGAC | AGAGUUUUAA | CUUUGUCUAG | CAGUCCGGUU | ACCGCGUACA | CGAGUCCAAU |
| filter240 | UUUGAUCCUC | UUUGGGUUGA | AAAAAAAGUC | GAUGGAGGGG | UCCAUUAGCC | UUUAAAUUAU |
| filter241 | CUGACUAUUA | GAGACUGGGA | UGGACAGAGU | AAGUCCACAU | AAGGAGGGCC | AGCGGUAGUU |
| filter242 | UUGGUAGAAU | AAAUCUUCUC | AUACGGAAAG | ACACUGUCCG | GACCUCGAAA | AAAGAAAAGU |
| filter243 | CAUAAAAAAA | UCCAUUCAUU | UUCAGAGUCU | CAUCCAAACC | AAAGAGACUA | CGGCGUCCUG |
| filter244 | UCAGUCUAAU | AAUUUUCCAU | ACUACAACCU | UUCCCUAGUA | AAGGUCCAUG | GAGUGUGUAA |
| filter245 | AGAACUGGAU | AAGCGACCAC | AUUGUAUGAA | AAAAAAAAUC | AUAGCAACGC | AUAGCACUAU |
| filter246 | UCCUACAGAC | UUUGUAGUCC | UUUAUCCUCC | CGAAUGAUCA | CAGCCUUAAG | AGGAAUUAAU |
| filter247 | UGAAUCUCUA | UACCAUUCAU | UCCGGUUGUC | UGUCCCAUCC | CGUUCCACCU | GACCACUCAU |
| filter248 | UCUGAAAAUU | UGGUCCCCUA | AAGAAACAAA | CGUUCCGCCC | AUUGUGCGAC | GUCAUGAGAG |
| filter249 | AAAUUCAUCC | UCAUAUUACC | CAGUUCGGCU | UAAAGAAAGU | CGCGAAAUGG | UCUCUCCACG |
| filter250 | UAAGUUCAAC | CCUCAACCUA | GUACCGAAAU | UCCAAAAGUA | UGUUGAUACA | UGGAUGUUUC |
| filter251 | GUUUGACCUA | GUACAGAACC | GGACAUGUGU | UAGAUUUUUU | AAGAACGAAC | UGACUUGAAA |
| filter252 | CUUCUCGAGA | CAUACAUUAC | CAUAACUAAA | GUAAUUUUAC | GAGAGAGCGG | GUAUGUCGAC |
| filter253 | UGAACUUUUA | GUUUCCAUUC | GGGAUAUUAC | CCGAAAUUGA | GUUGCAUCCA | GCGACUCAAG |
| filter254 | GAAUCAAGUA | UUGACGAUUC | AGCUCACCUU | CAUAAAAGUC | CAAAGCGAAA | CUGGUGUAAG |
| filter255 | UAUAAGGUGU | GUGUGUCCAU | AGGACCUCGA | UGAGGUCGAA | AAACAUCAGC | GCUUCUGCUG |

**Table S10.** CircCNN input1(SA input) motifs shared between: human, mouse, and fruit fly. Motifs shared by all three species are highlighted in red.

| Filter ID | Human motif | Mouse motif | Filter ID | Human motif | Fruit fly motif |
| --- | --- | --- | --- | --- | --- |
| filter2 | CCUACAAAAA | UAUACAGACC | filter15 | AUCCGUCUUC | AUGUCCAUUC |
| filter3 | UUCAGACCUU | UACCGUCUCU | filter19 | CGUCUUUCUU | GGUCAUUCAU |
| filter13 | CAGUGGUUUA | CAGAGAAAAA | filter29 | CGUCUCGAAA | GGUAACUAUA |
| filter16 | AUUUAUUUUA | AAAUUUUAAA | filter46 | UACACAACAG | UACACGUGUU |
| filter21 | CAAUACUAGC | AAACCCCUGC | filter53 | AAAAUUUACA | AUCCUAUACC |
| filter25 | CUUGACGUUA | CAUGCGUCUA | filter58 | UCAUAUUACC | UGACAUUGUU |
| filter38 | UCCACGUCCU | AAGACACCCU | filter63 | GAAAAAAAAA | AAACAAAGGG |
| filter39 | AAAAAUCGUU | AUCACUCUUU | filter68 | UCACAAGUAA | UAAAAAUAAC |
| filter42 | AUGUCAUUUU | UAGUCAAUCC | filter103 | AUAUGAGGAA | AUAUAAAAUC |
| filter49 | UUUUCUCAGG | CAUUUUCUGU | filter105 | UAAUUAAGAA | UAAGAGAGAU |
| filter95 | AUUUGUAACA | UGUCGCCACA | filter118 | ACUUUCUCAC | UCUGUCUCAU |
| filter105 | UAAUUAAGAA | AAGAUAAGUC | filter121 | AAUUCAGAUC | AAGUAAUCAC |
| filter113 | ACAUCAAAAG | GACUAAAACG | filter142 | UGACGAAAAU | AGAGGAAGCG |
| filter118 | ACUUUCUCAC | UGUUCCCUAC | filter155 | GUAUCGAACC | GUUUCUAUUU |
| filter154 | AAUUUUGUGA | UAUUUCUUCU | filter167 | CCCUGGAUUA | GUCAGUUUUA |
| filter164 | UCAAAAAGUA | GAAAAAAGAA | filter183 | AUCUGAAGAA | GUCCGAUCAG |
| filter167 | CCCUGGAUUA | CCAUUCAUCU | filter187 | CAAAGAAUCA | CAUCCAUCCA |
| filter181 | GAAGGUCGUC | GGUGUGCGUC | filter206 | AGUCUAUCUC | UGUGACUGUC |
| filter206 | AGUCUAUCUC | UGUUAAUGAC | filter229 | UUUUAGUUAU | UAUUAACAAA |
| filter215 | CUUUUAUUUU | CACUCUUGUU | filter238 | AGAACGUCCG | UGAGCAGUCG |
| filter224 | UAUACUUUUG | UAAGGUGUUA |  |  |  |
| filter230 | ACAUAUAUAA | UCACAUCCAC |  |  |  |
| filter236 | UUUGUAAAAA | AUUGUCGUGA |  |  |  |
| filter249 | AAAUUCAUCC | CAGUUCGGCU |  |  |  |
| filter252 | CUUCUCGAGA | CAUAACUAAA |  |  |  |

**Table S11.** CircCNN input2(SD input) motifs shared between: human, mouse, and fruit fly. Motifs shared by all three species are highlighted in red.

| Filter ID | Human motif | Mouse motif | Filter ID | Human motif | Fruit fly motif |
| --- | --- | --- | --- | --- | --- |
| filter13 | CUUCGAAACC | UGAGGAAUCC | filter17 | UUAAUUAAAU | UGUAUCAACA |
| filter14 | UCUUUACACC | UAGUUGUAGC | filter22 | UGUGUUAUAA | UGUUAAAUUA |
| filter24 | ACCAUAUUUG | ACACGUUCUG | filter58 | GCACUUAUCG | AAACUUCACA |
| filter33 | UCAGAGUUCC | ACAUUGAUCA | filter65 | AUGAAGUGUG | AGGAAAGGGU |
| filter37 | AACGAAUGAC | AAAAAAACAA | filter102 | AGAGACGACU | ACAAAGAACG |
| filter40 | UCCAUUCGCG | UUAAUACACU | filter103 | UACGGACAGG | GACUGACGCA |
| filter62 | CCCUCAAAUA | CAUUCAUUCA | filter120 | AAAAAUUCCA | AUAAACGUCA |
| filter88 | CAAUAUUGUC | AGUUUUUUUC | filter124 | ACUCAAAAAG | AGCUAAGAGG |
| filter93 | GAGUACCAUU | AAAGACCAGA | filter159 | AAAUACGCGG | AACAAGGGAG |
| filter94 | GGACAAAGAU | UUACCAAAUU | filter185 | GACUAAAGUC | AGCGAAAGGA |
| filter114 | ACGGUUCAUG | AGUGUUGACA | filter188 | UAUCUUUUUA | AAGUCUUGUA |
| filter120 | AAAAAUUCCA | GAUGUCUCCA | filter202 | UCCAUAACUG | UAAAUGGCUU |
| filter139 | GUCCCUUUUC | GUCCCUGGGG | filter211 | AAAAACCAUC | AAAAGACAAC |
| filter142 | CCCUCACACG | UCGUCAAUAG | filter221 | AGUUCCAUUC | UCCUCUACUC |
| filter151 | UGUUCCAUAC | UGUAAUCUUC | filter224 | AAAACCUCUA | CAUACAUUUA |
| filter162 | GACCCAUCCA | UACCAUCCAA | filter238 | ACUCAUAAGA | GGACUAAAGA |
| filter181 | AGAGAACGAG | AGGUCACAGG | filter239 | AGAGUUUUAA | CGAGUCCAAU |
| filter186 | UUCGUGACGU | UUCUCUACUC | filter240 | UUUGGGUUGA | UUUAAAUUAU |
| filter192 | ACCCAGGUAG | AAACAGCAAG | filter247 | UACCAUUCAU | GACCACUCAU |
| filter198 | GAGGCUAACC | CAAGACAACG |  |  |  |
| filter208 | GUAGACUCAG | CUAAAUUGAA |  |  |  |
| filter209 | AACAAACAGG | GAUUCAAAGG |  |  |  |
| filter248 | UGGUCCCCUA | CGUUCCGCCC |  |  |  |
| filter250 | CCUCAACCUA | UCCAAAAGUA |  |  |  |
| filter252 | CAUACAUUAC | GUAAUUUUAC |  |  |  |


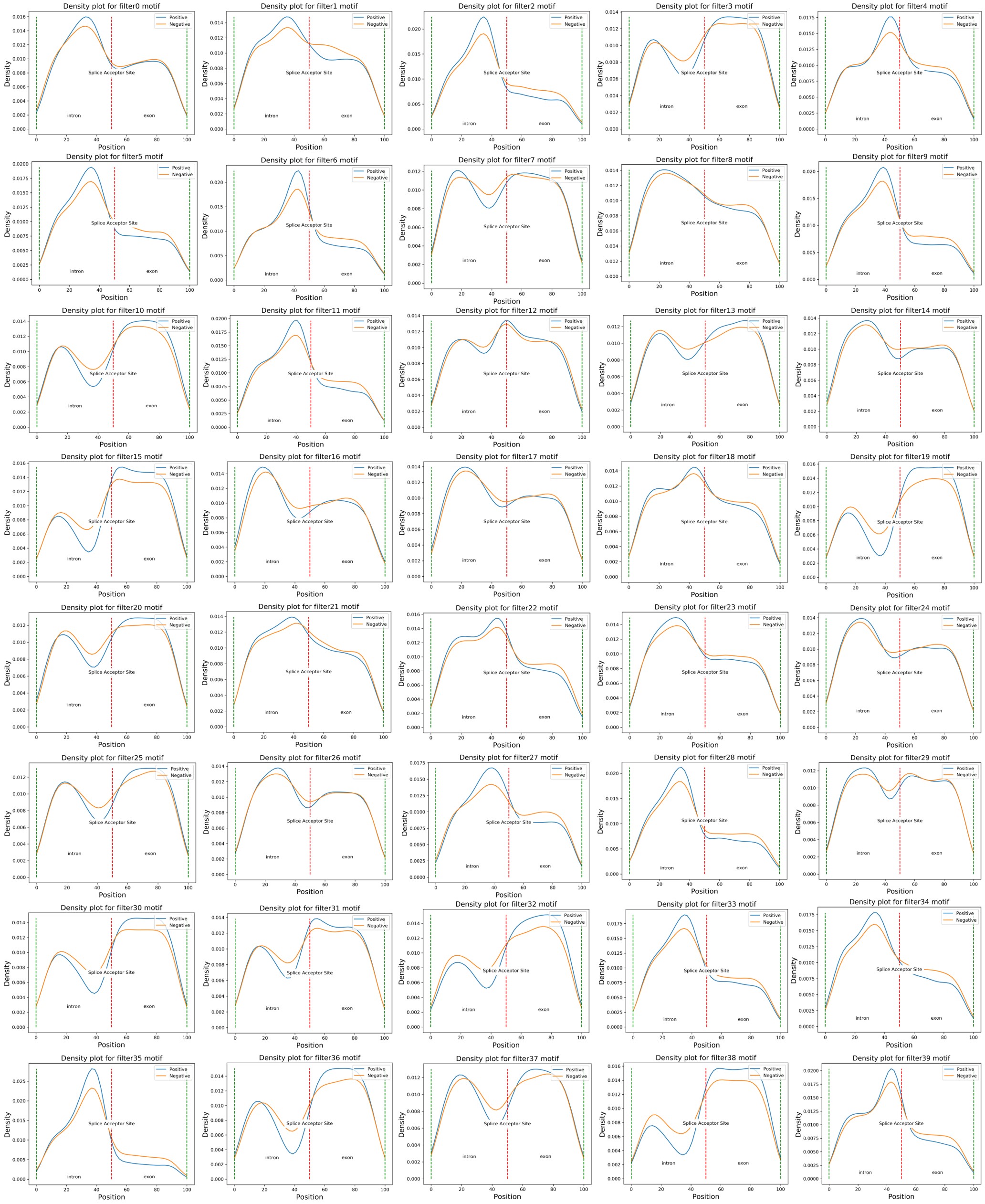


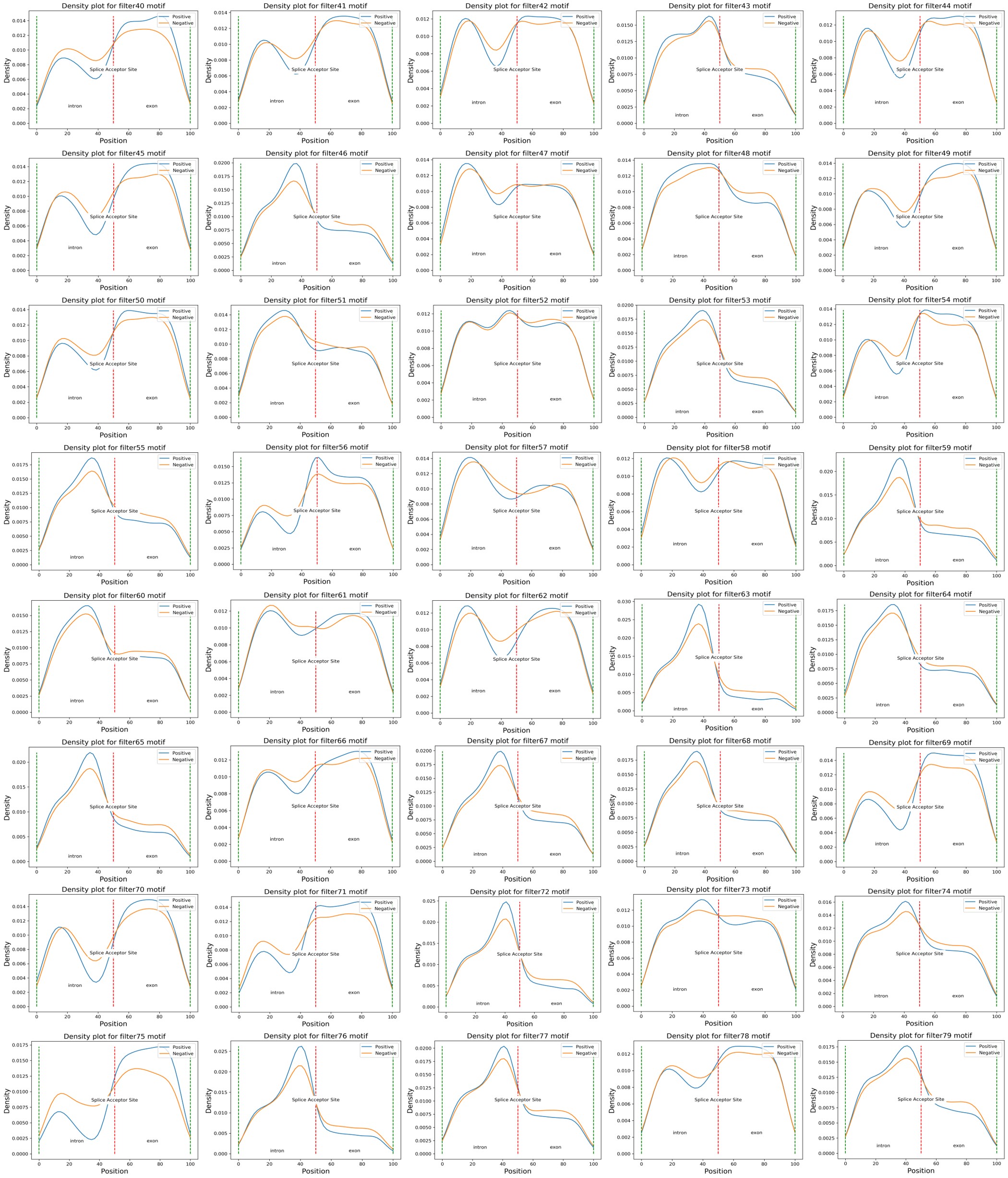


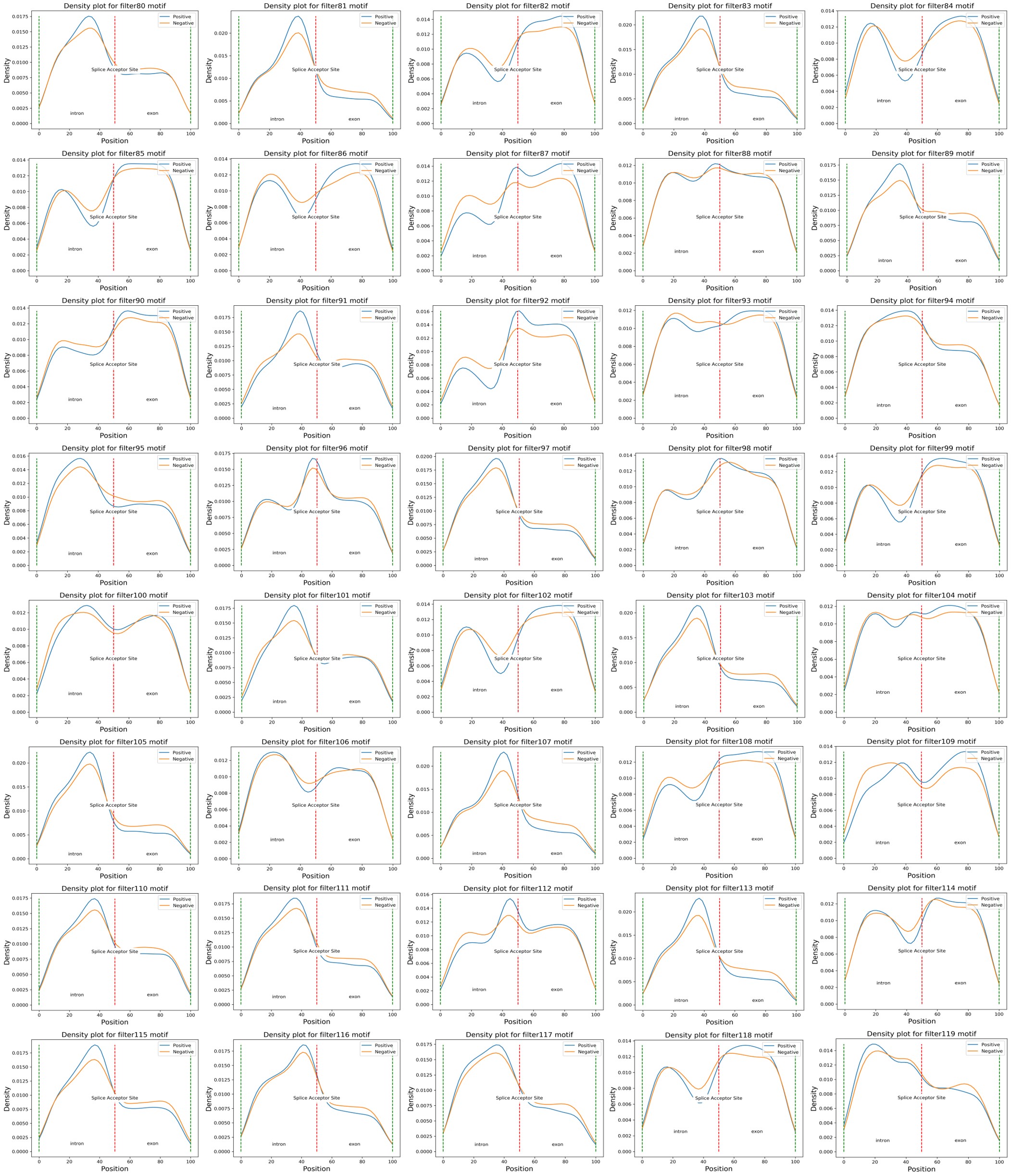


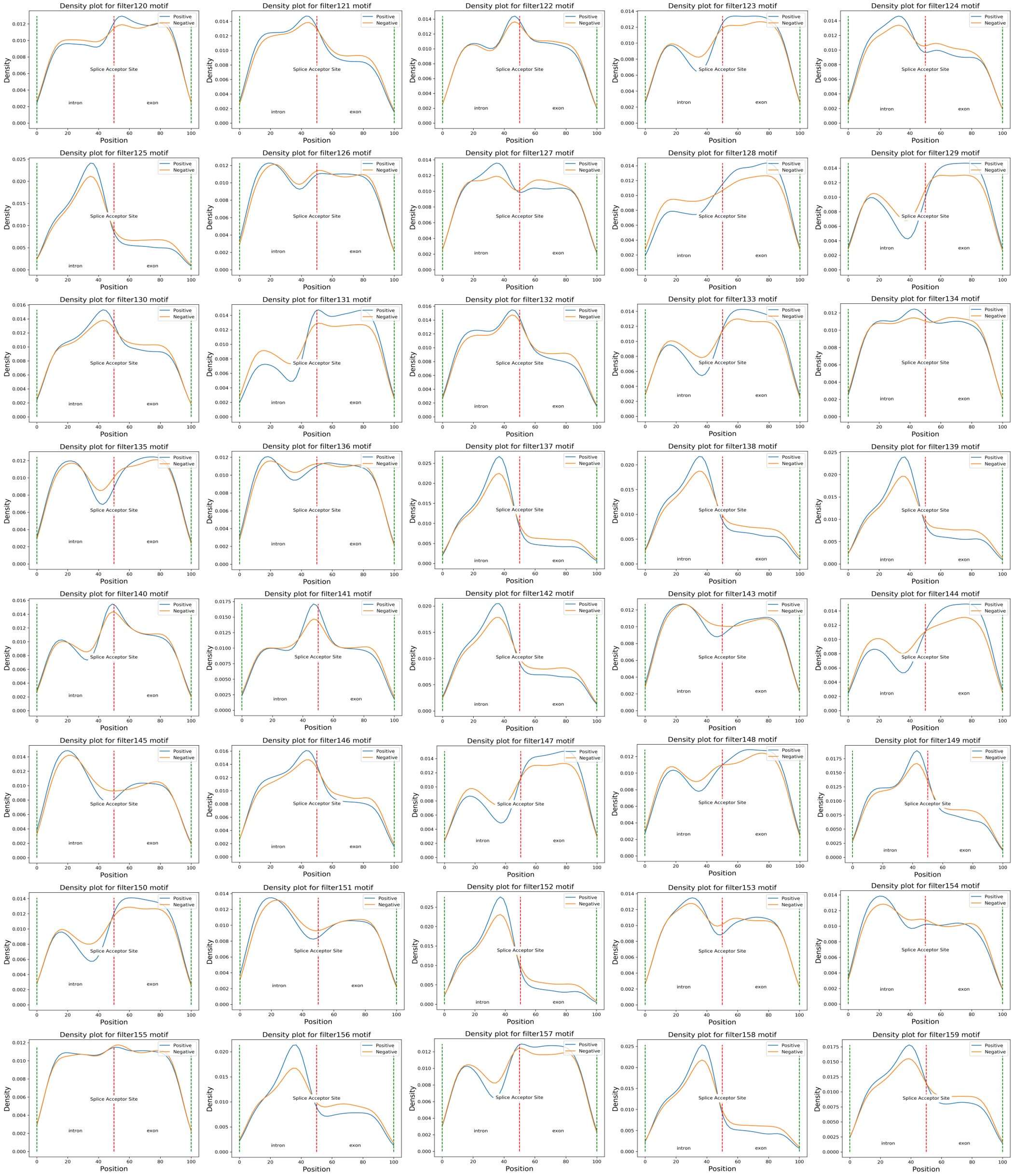


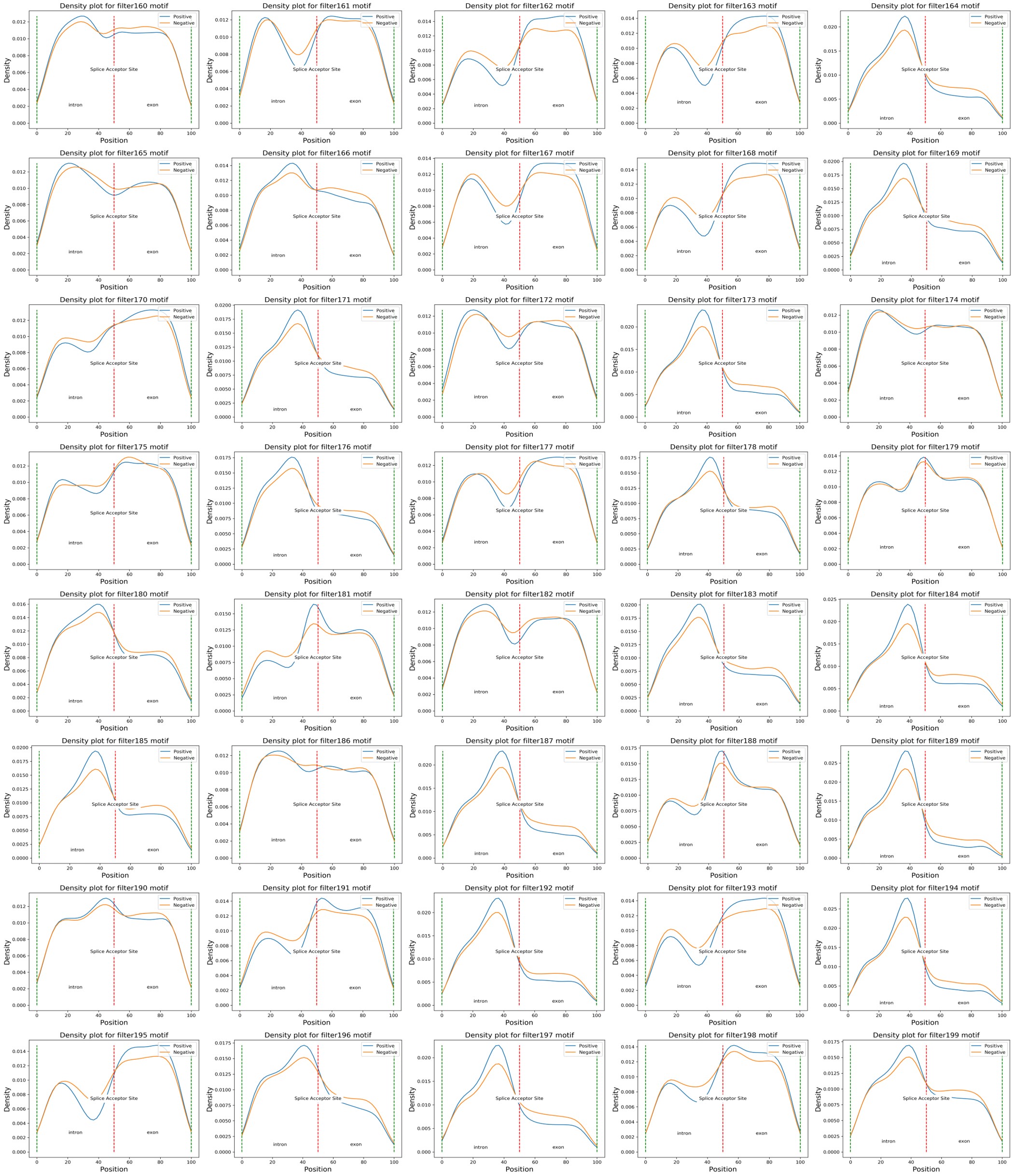


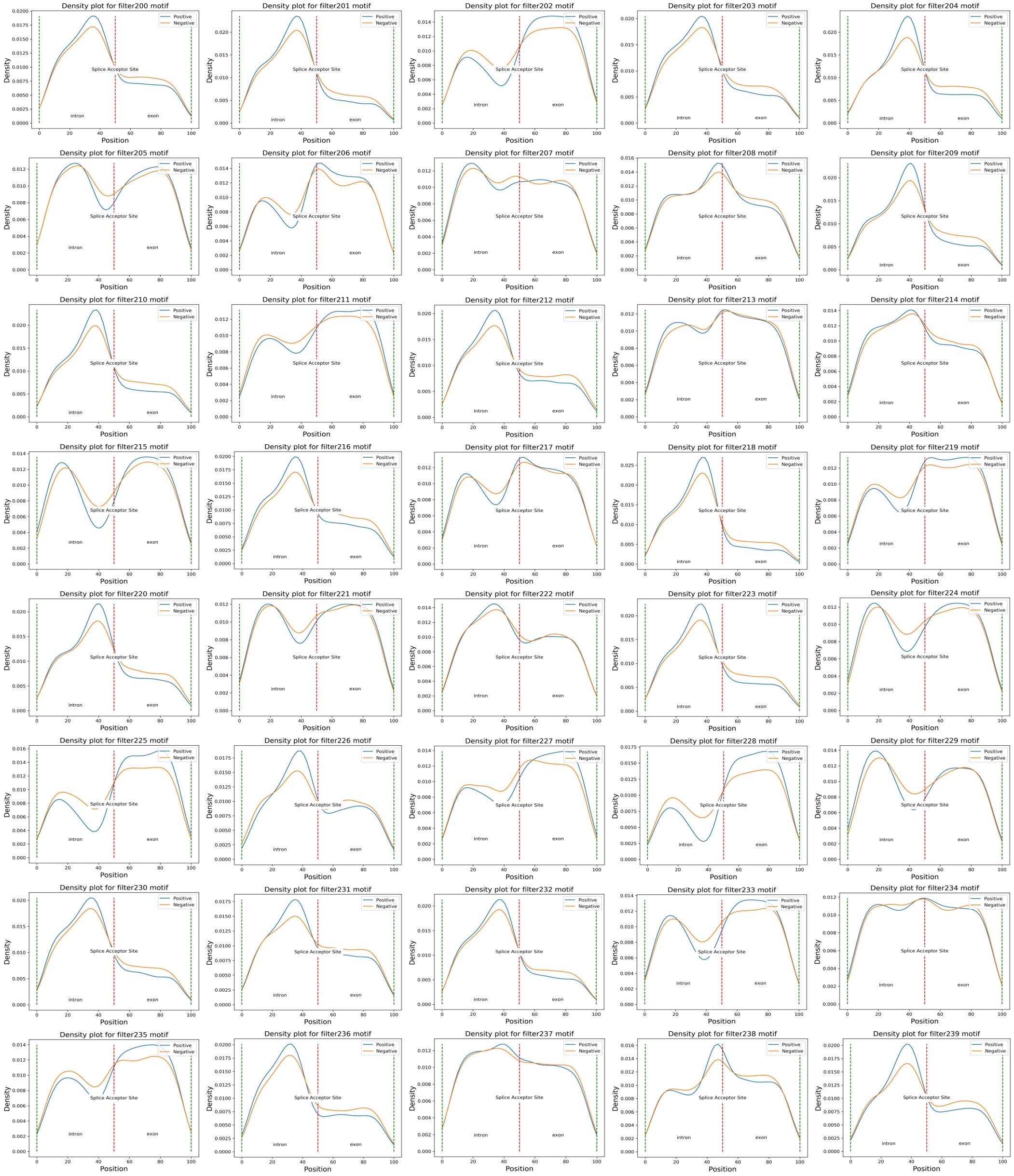


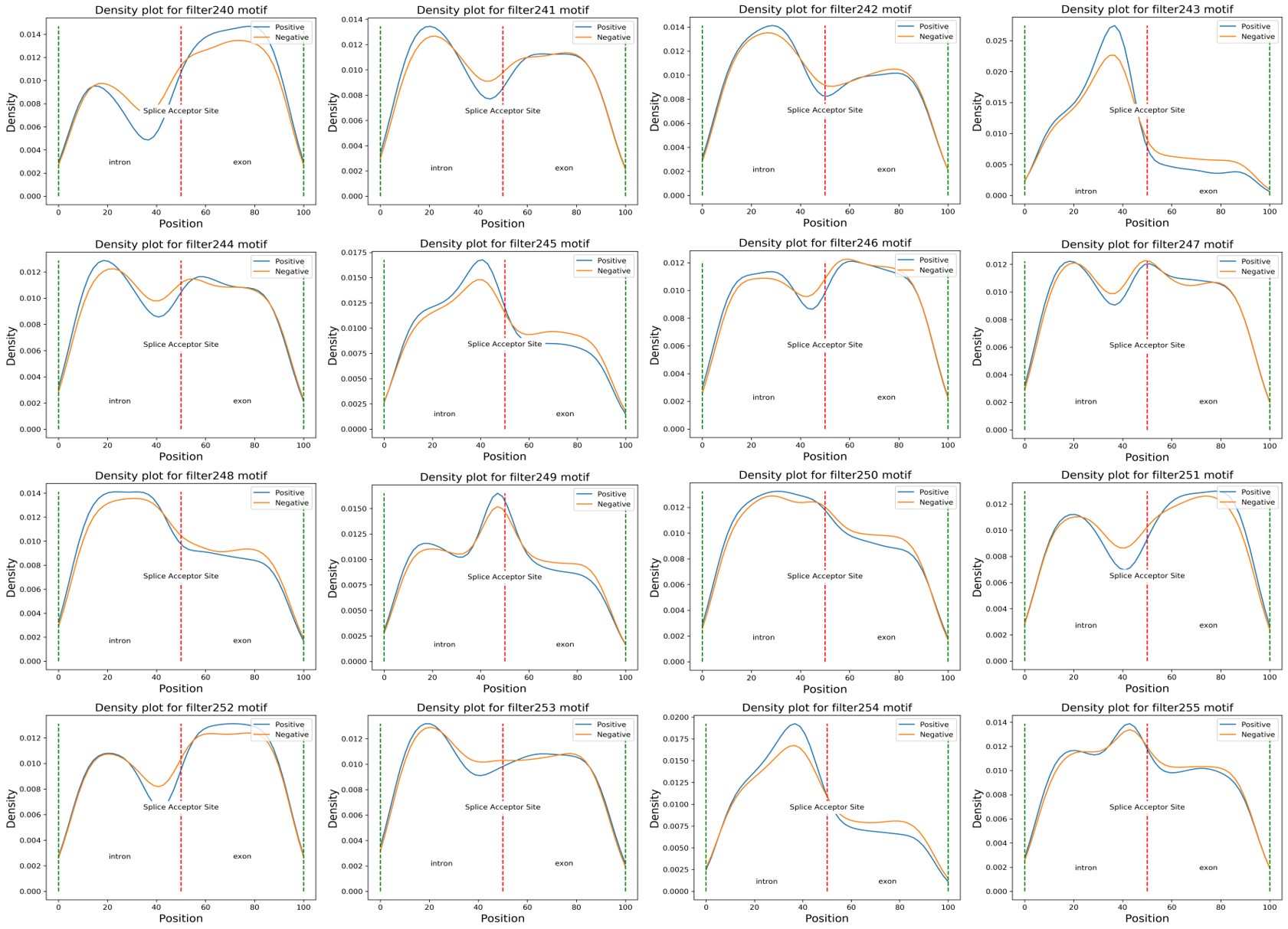


**Figure S2.** Distributions of human motifs found by circCNN in the positive and negative human circRNAs input1 (SA input). Here, blue line represents positive samples, orange line represents negative samples, red line represents splice acceptor site, and its left and right are intron and exon, respectively.


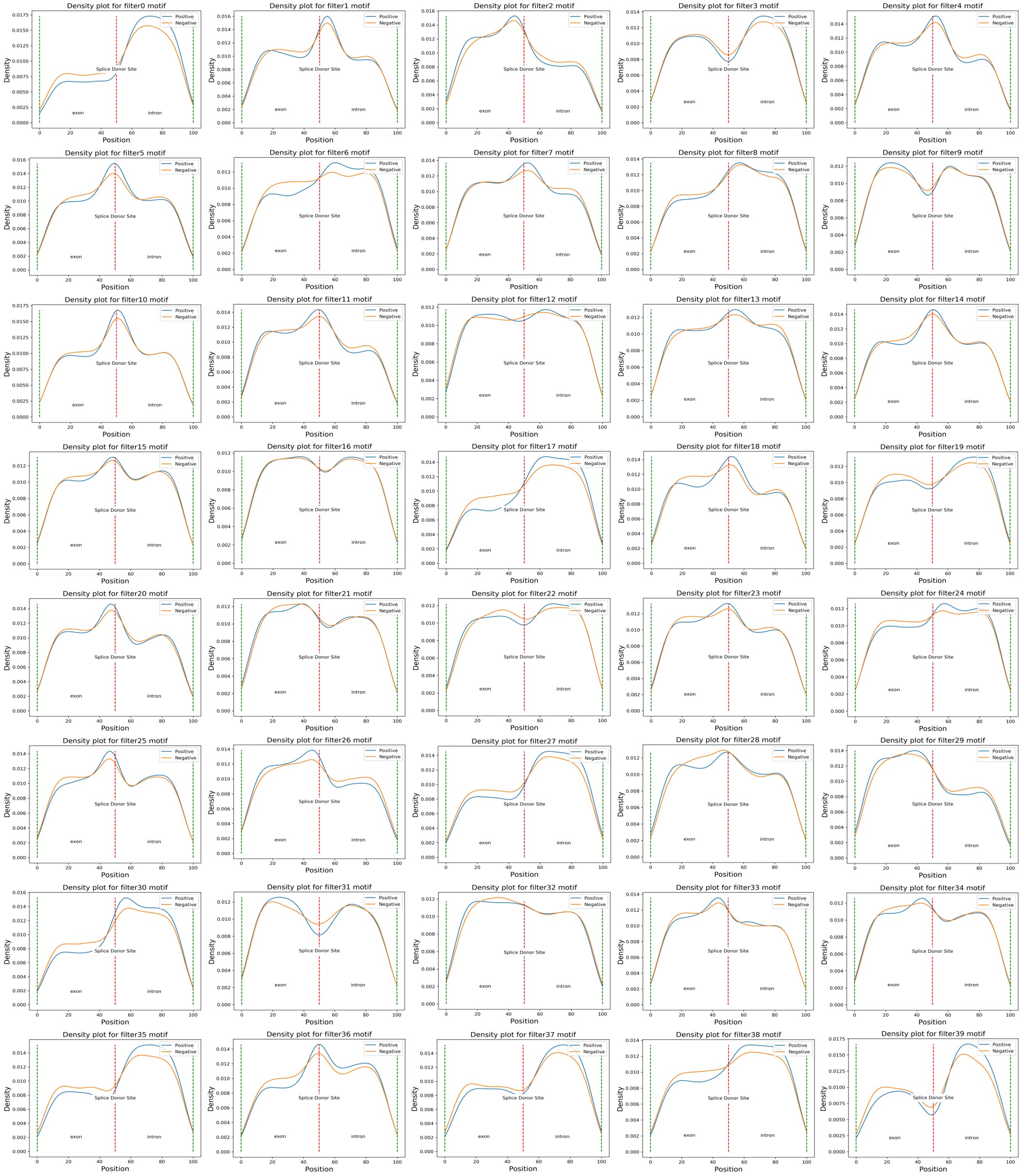


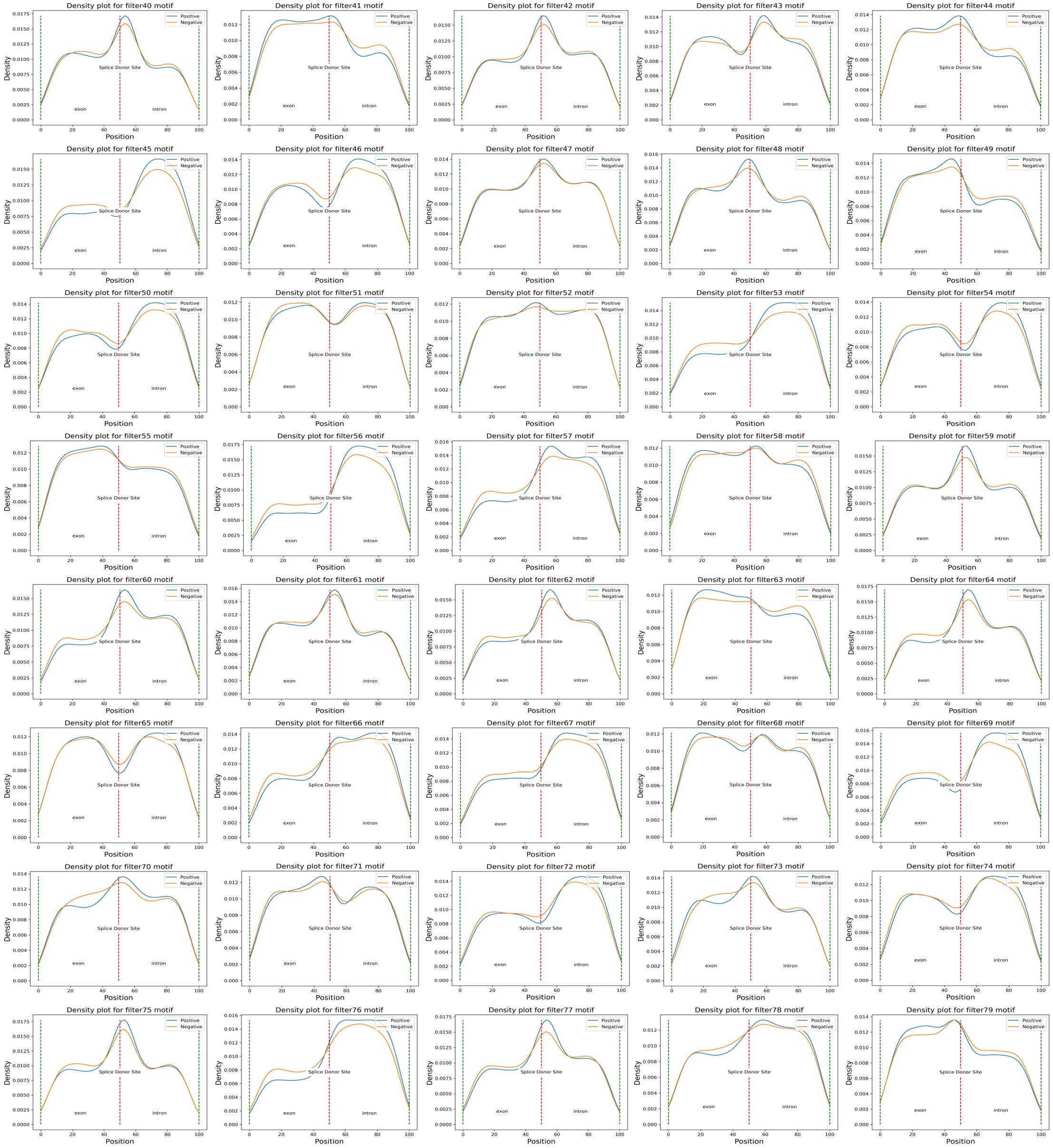


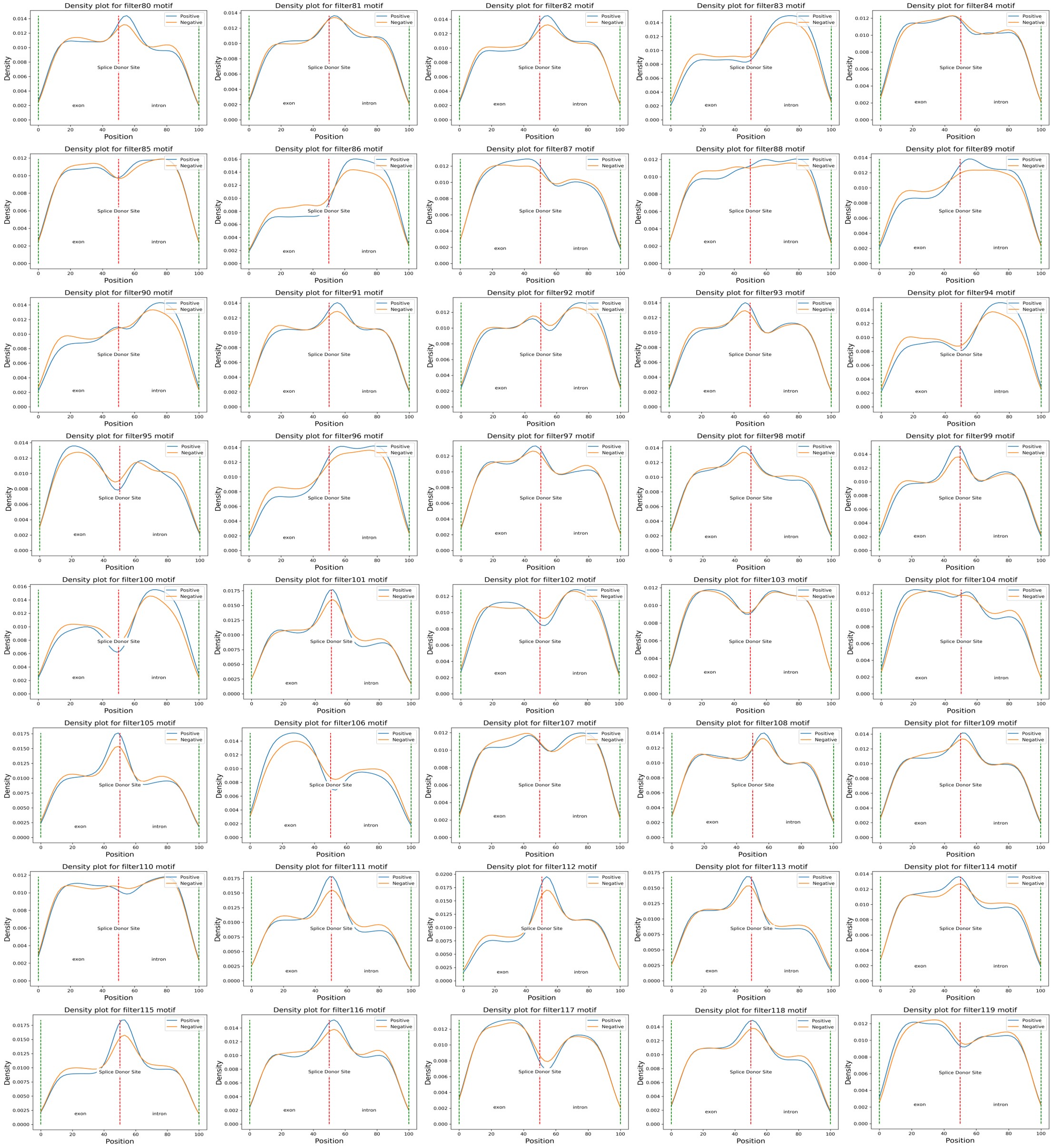


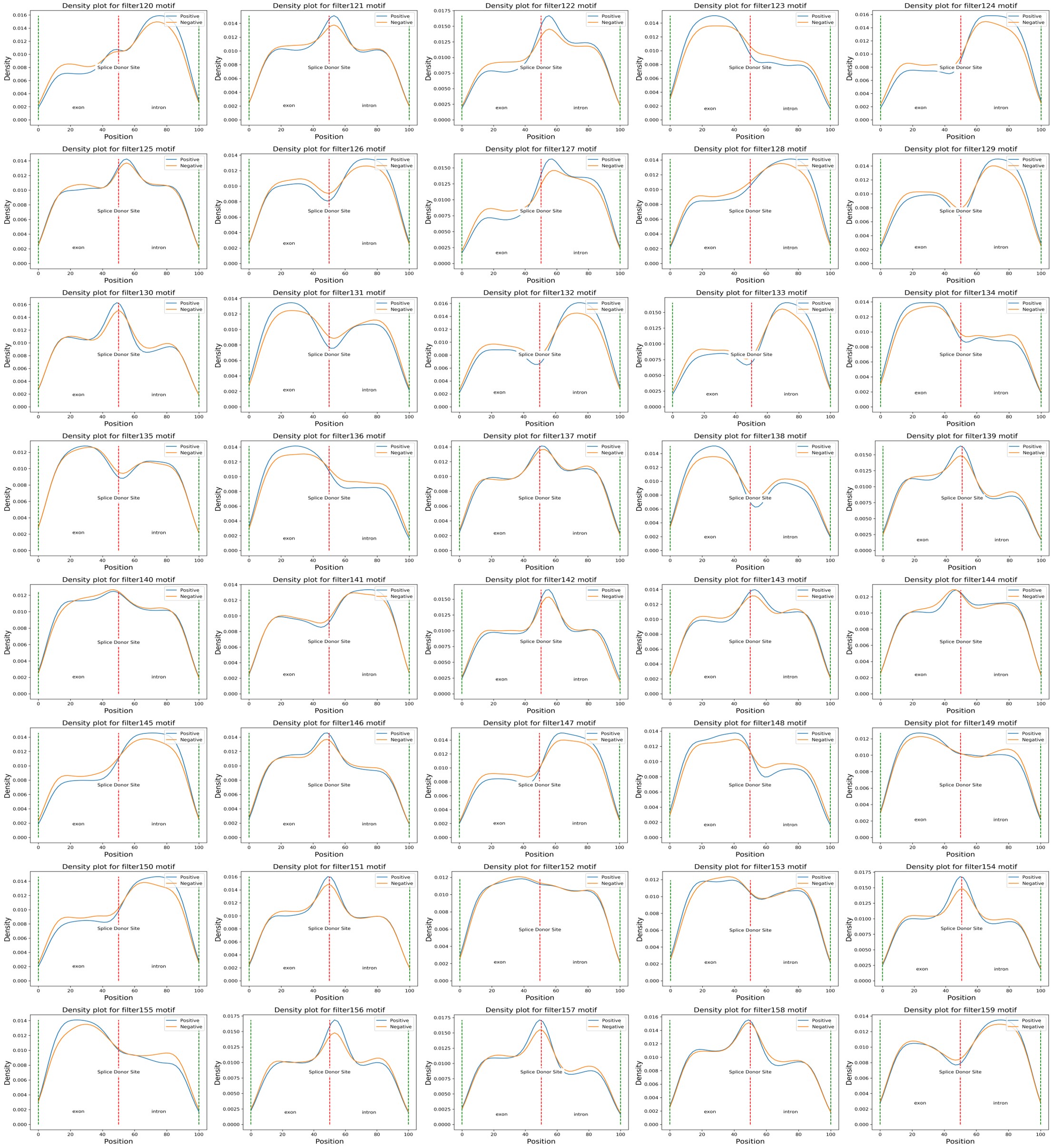


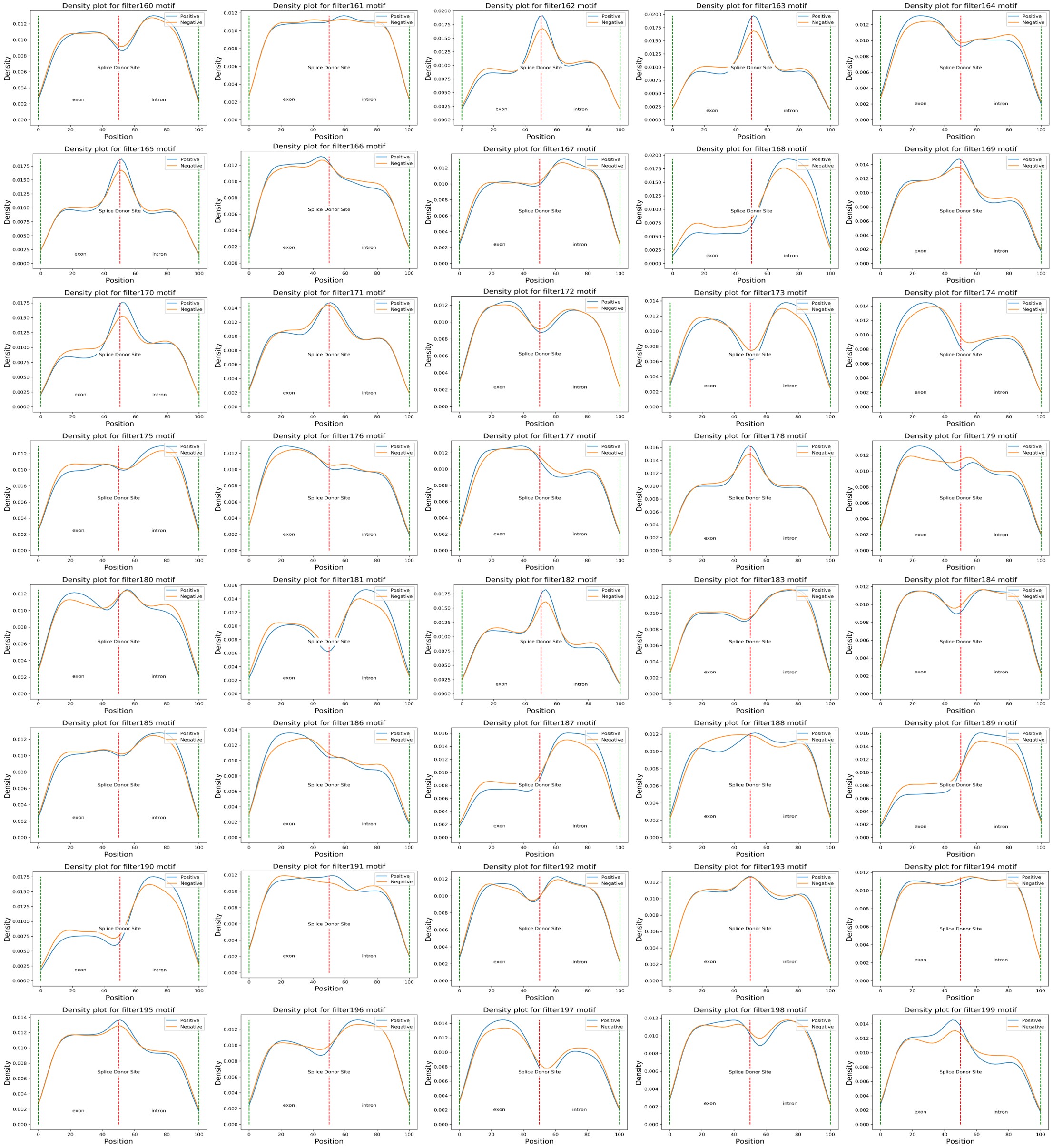


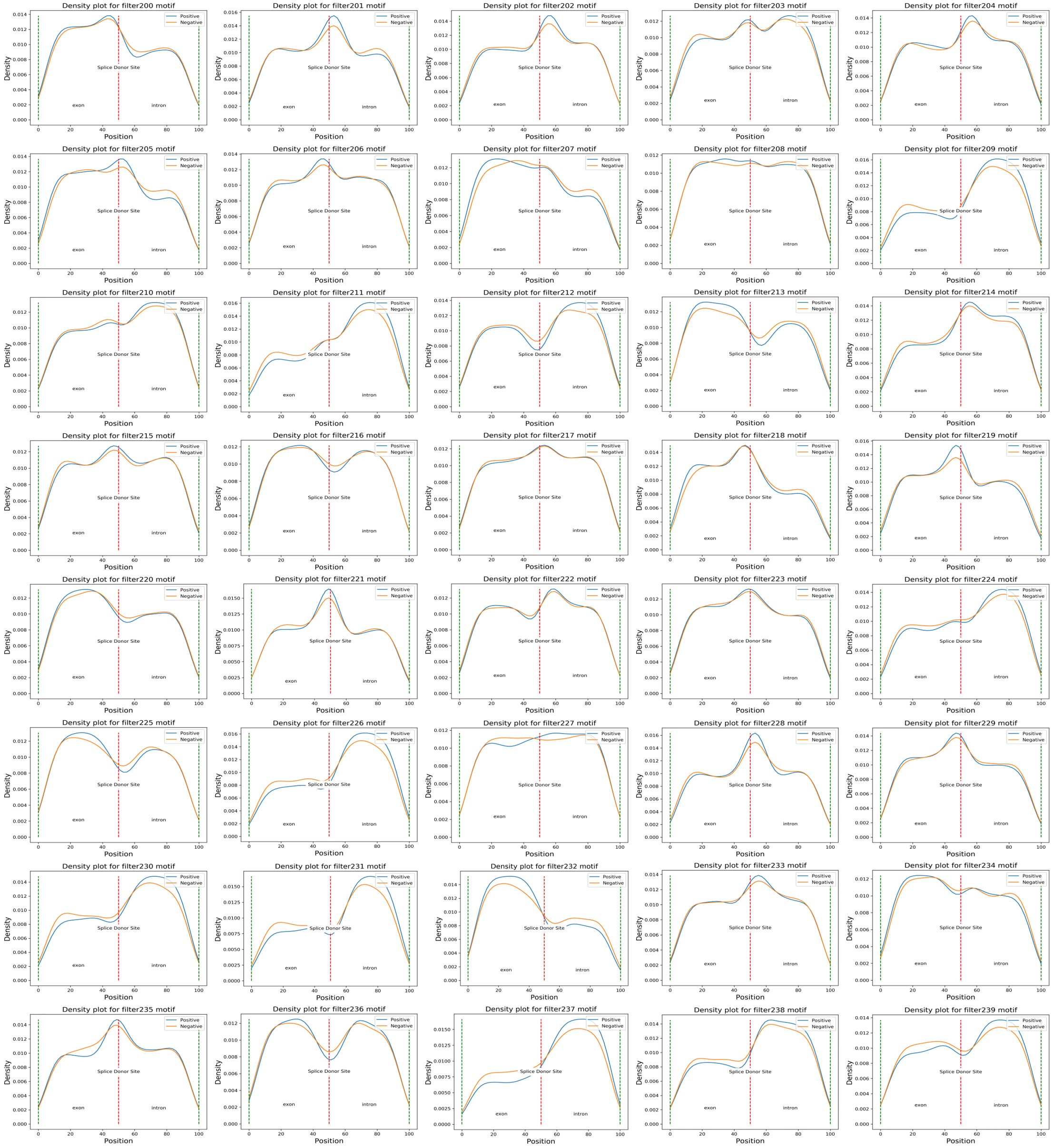


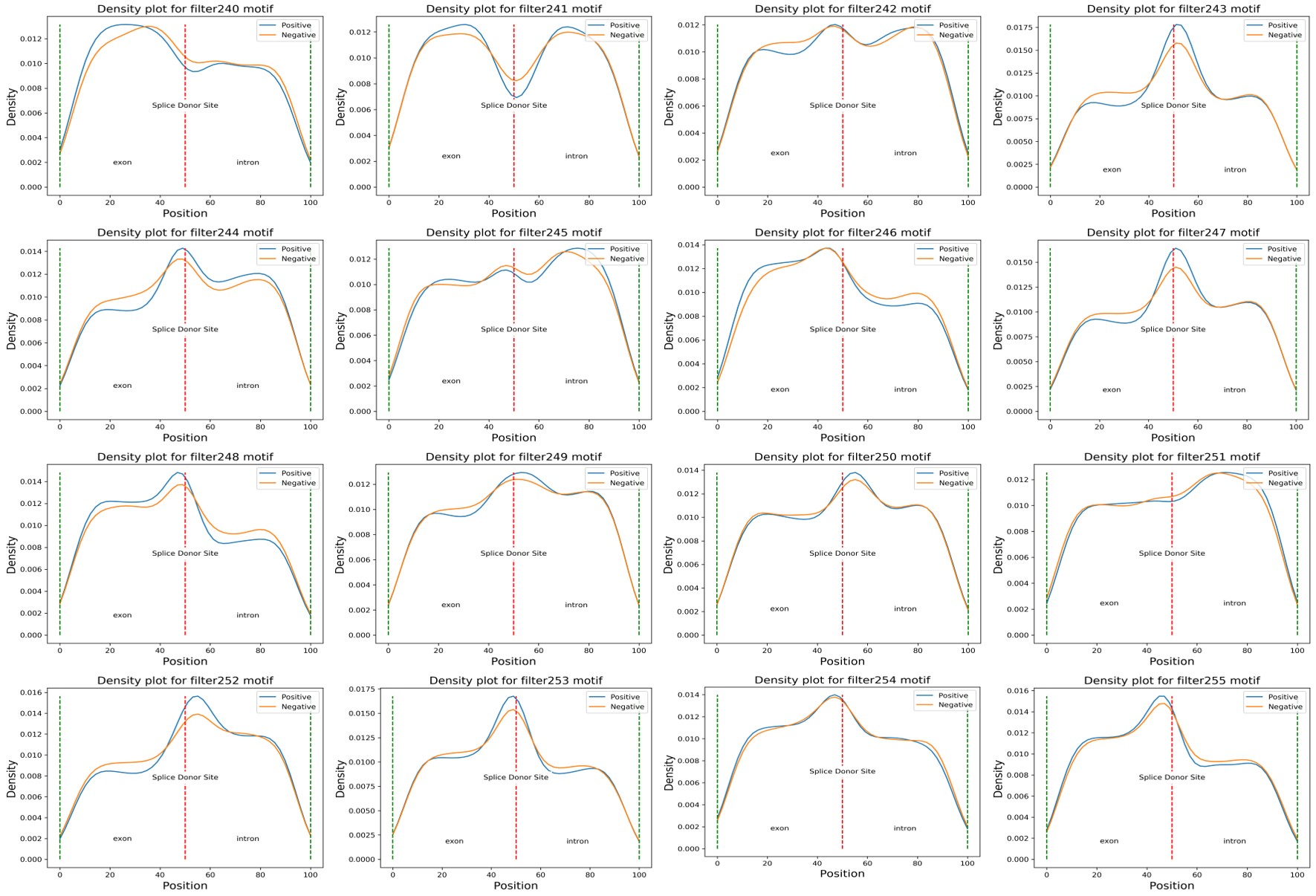


**Figure S3.** Distributions of human motifs found by circCNN in the positive and negative human circRNAs input2 (SD input). Here, blue line represents positive samples, orange line represents negative samples, red line represents splice donor site, and its left and right are exon and intron, respectively.


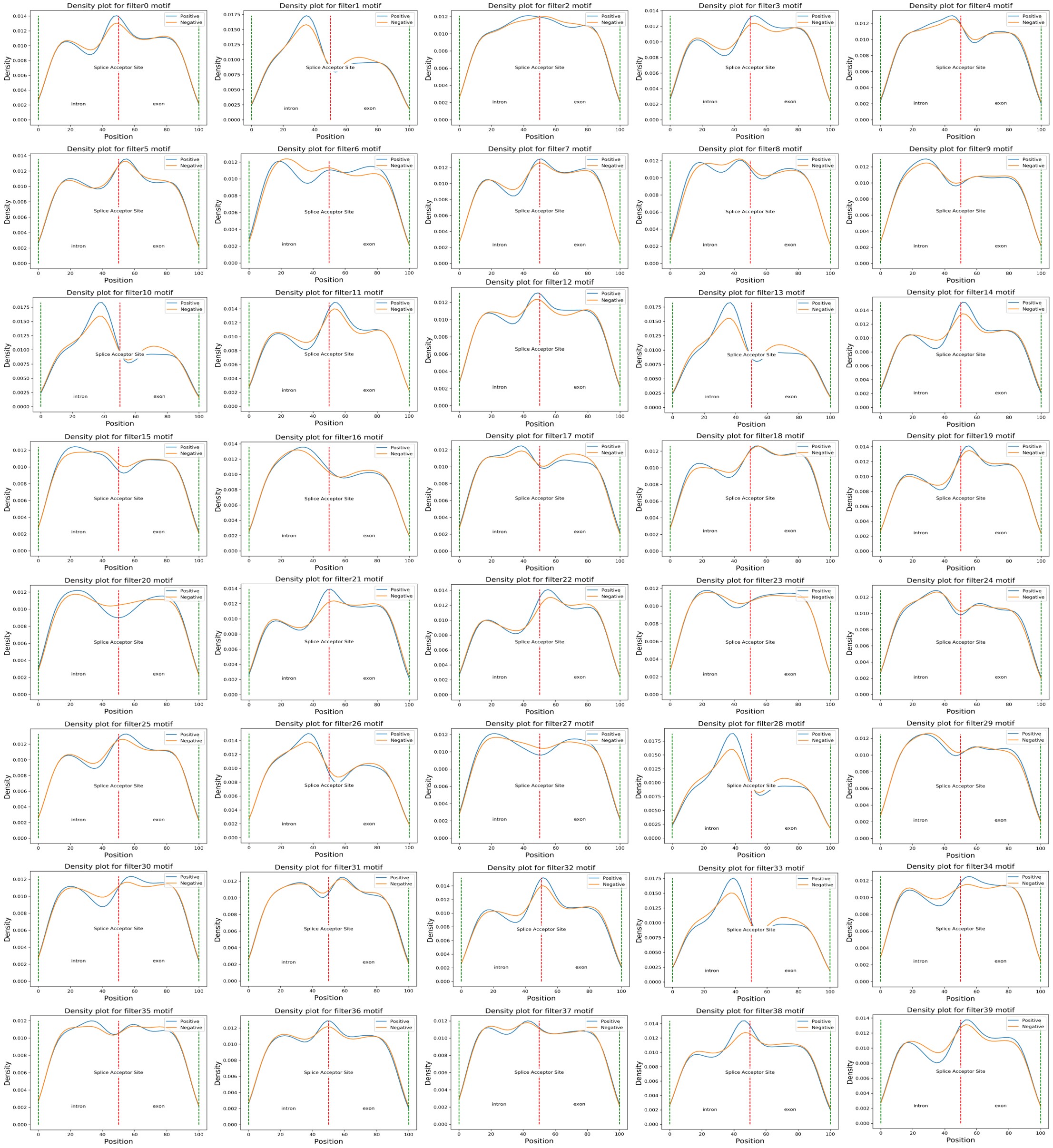


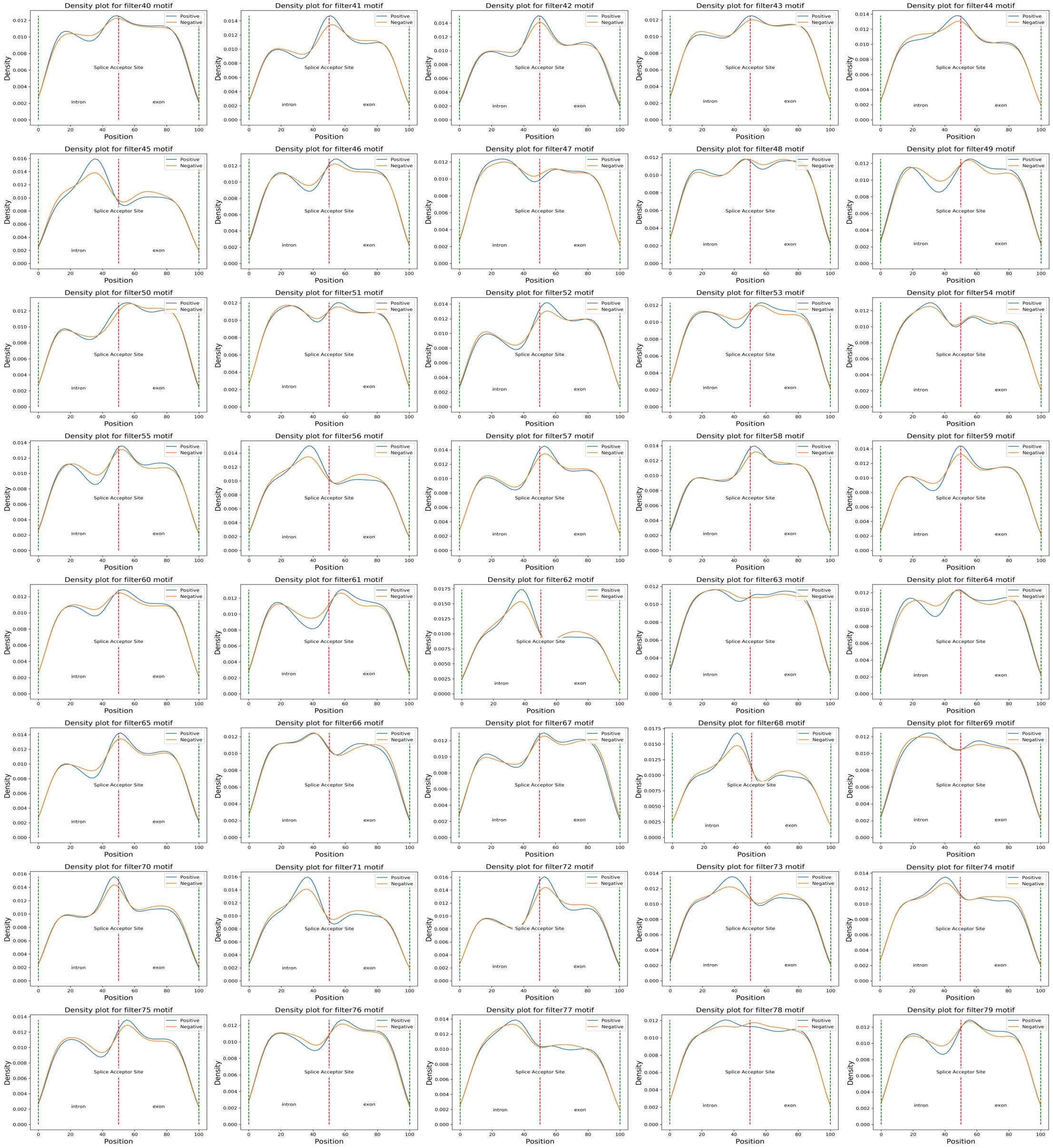


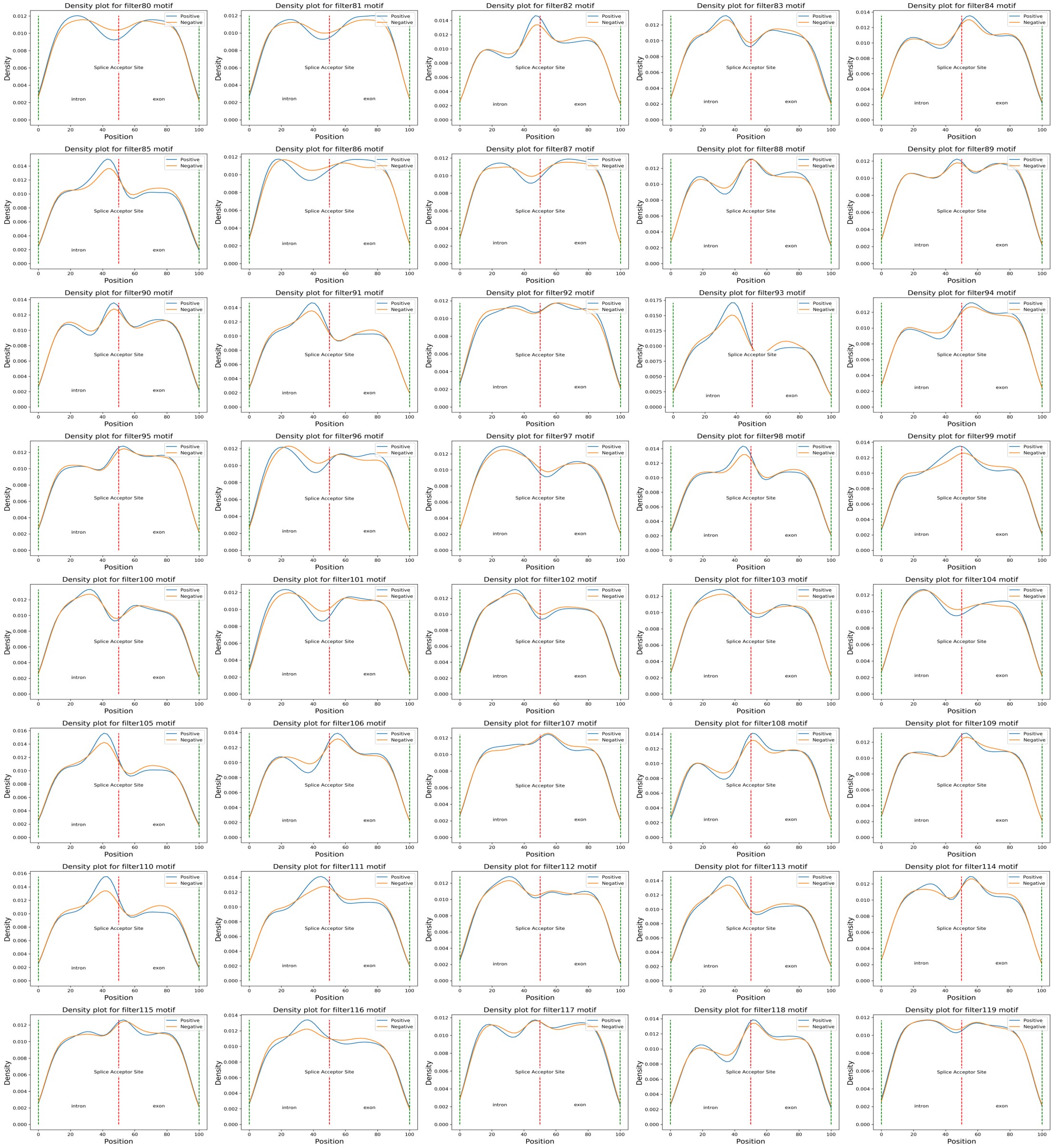


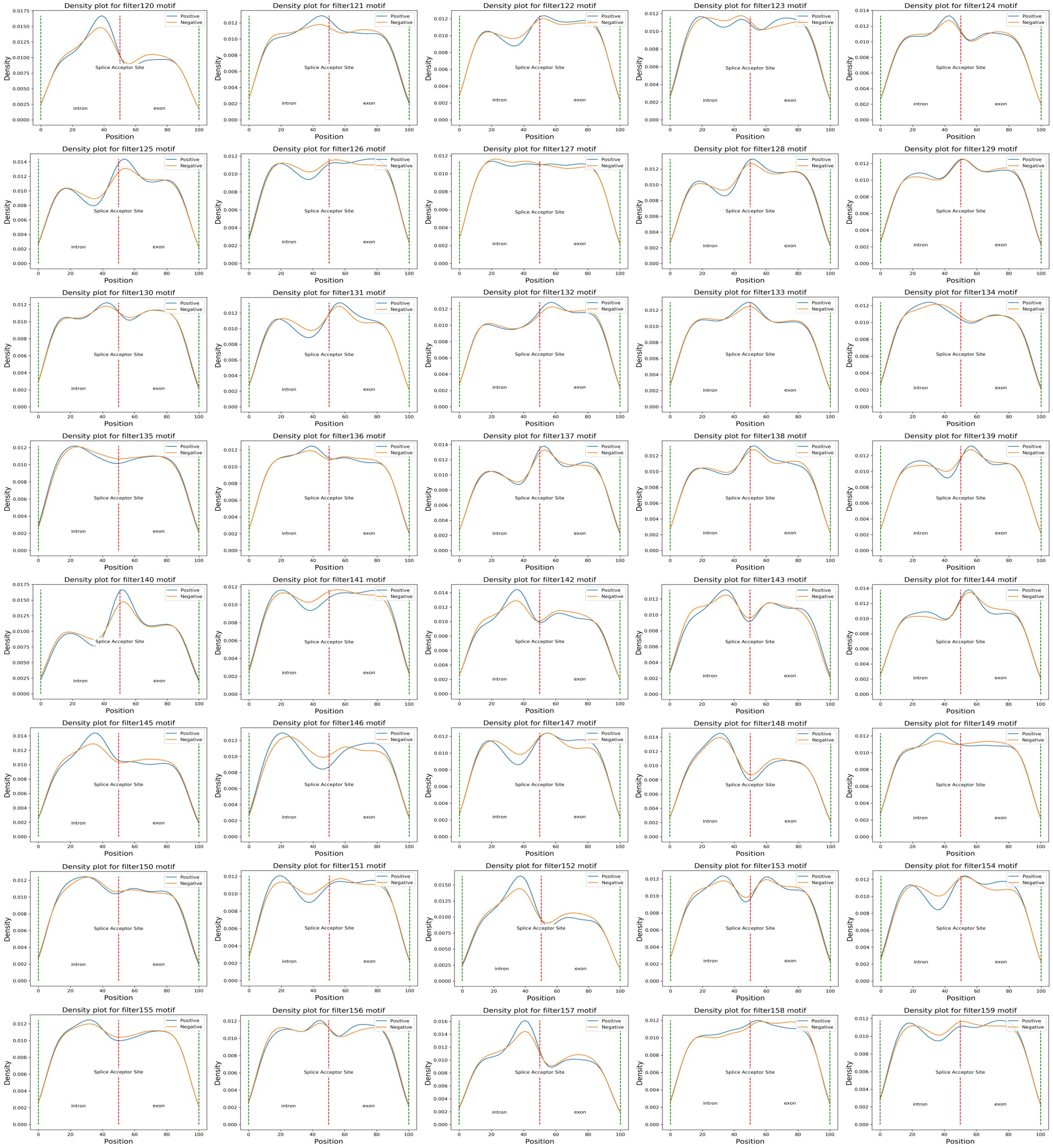


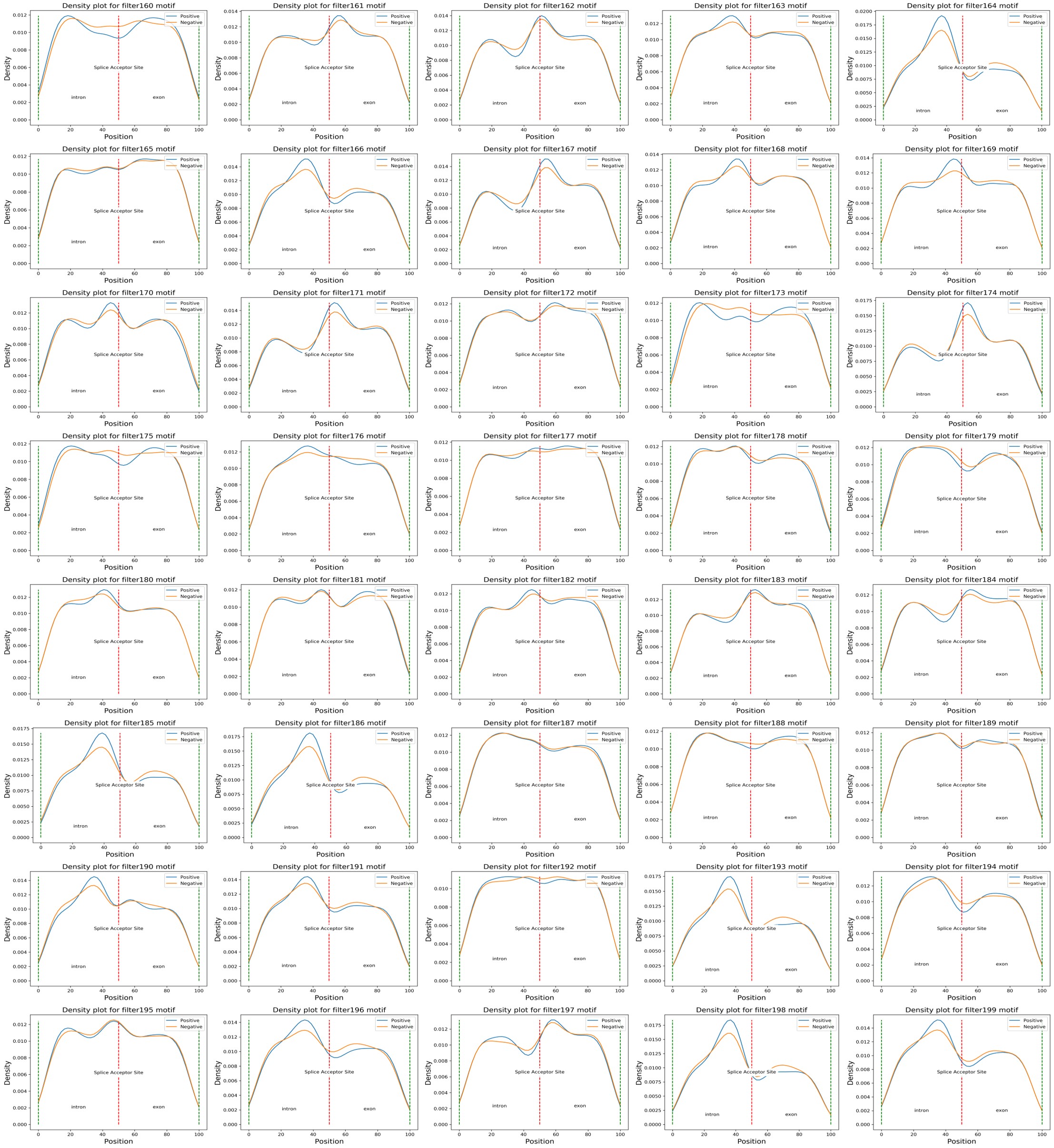


**
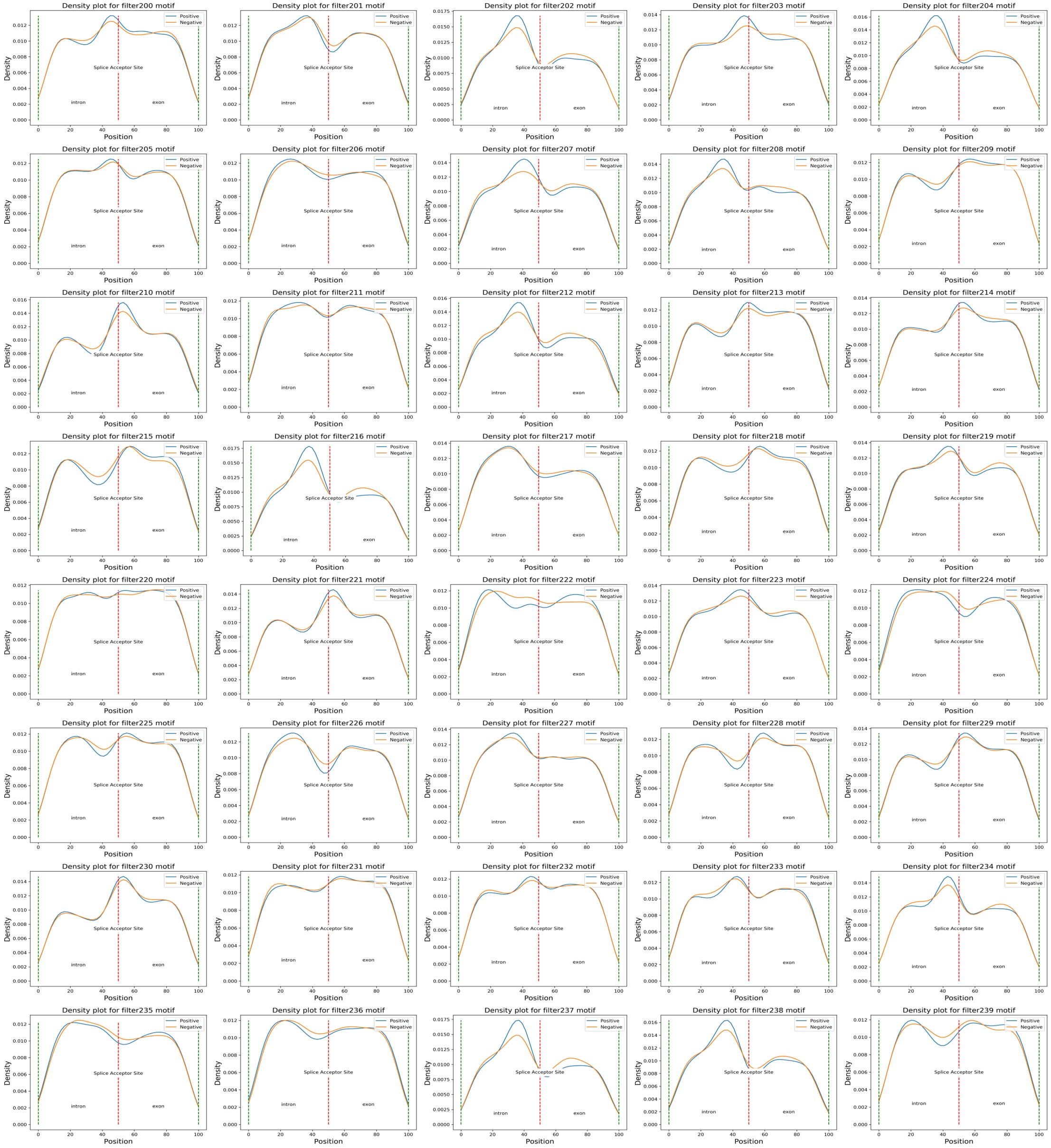
**

**
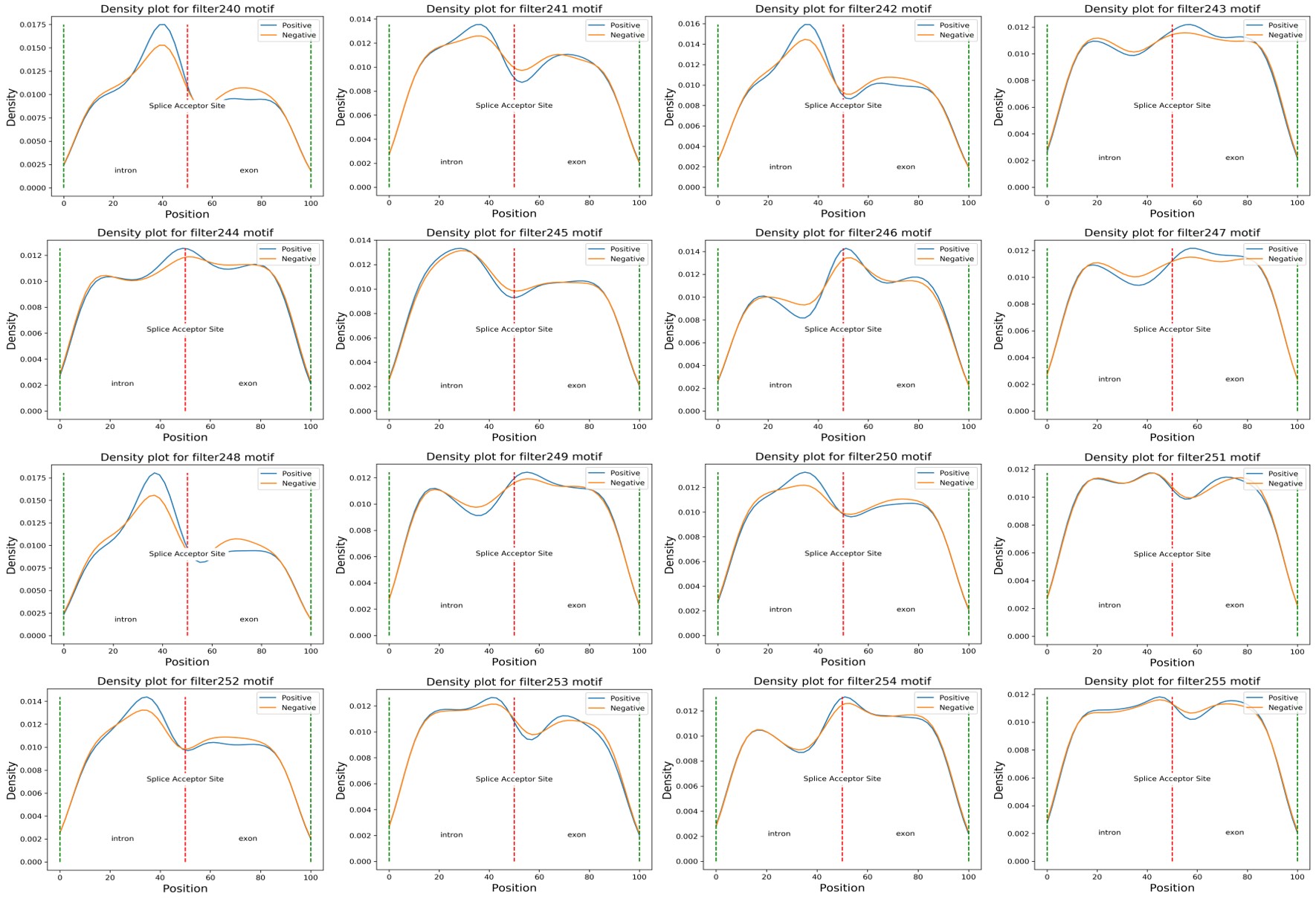
**

**Figure S4.** Distributions of mouse motifs found by circCNN in the positive and negative mouse circRNAs input1 (SA input). Here, blue line represents positive samples, orange line represents negative samples, red line represents splice acceptor site, and its left and right are intron and exon, respectively.


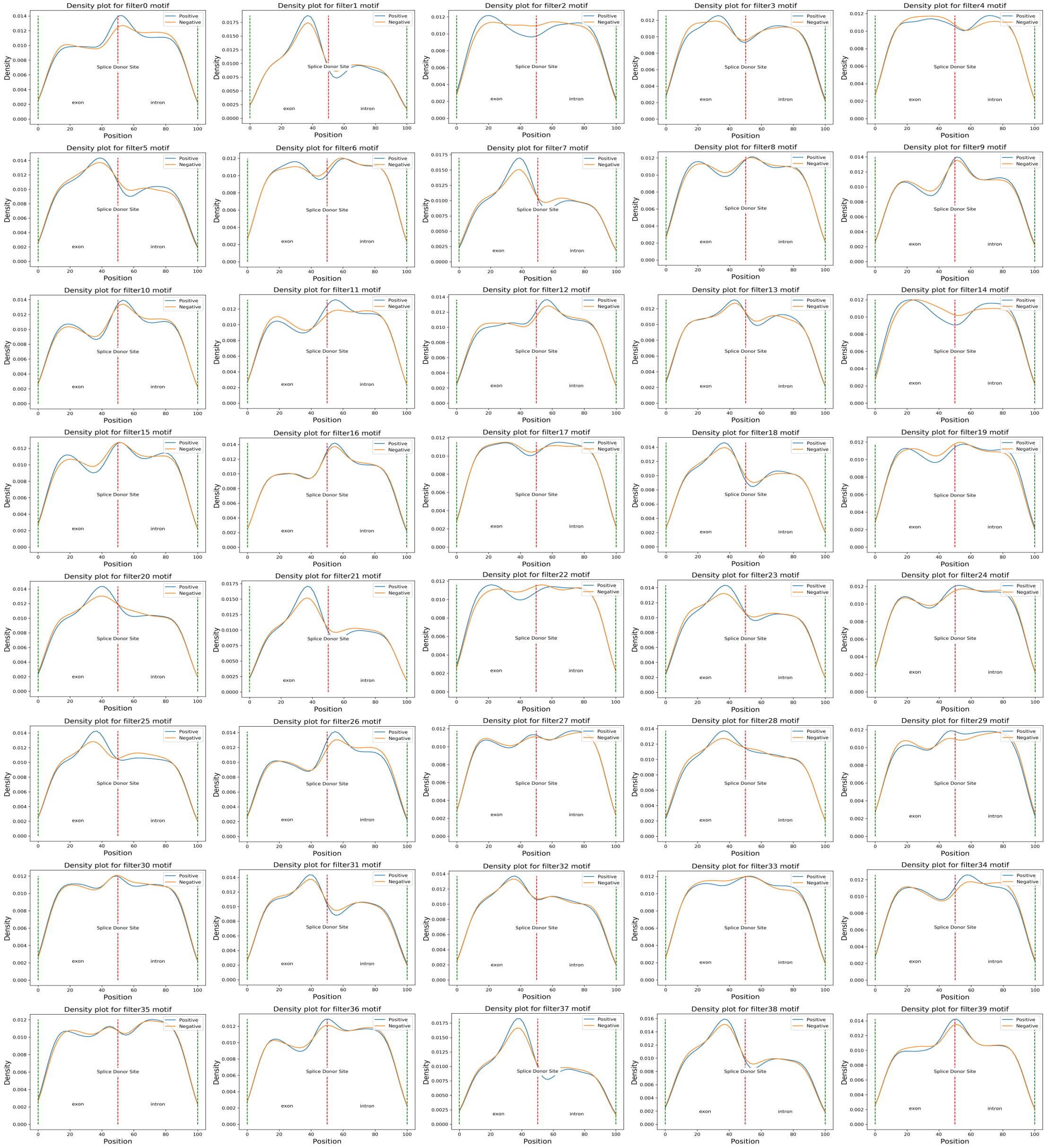


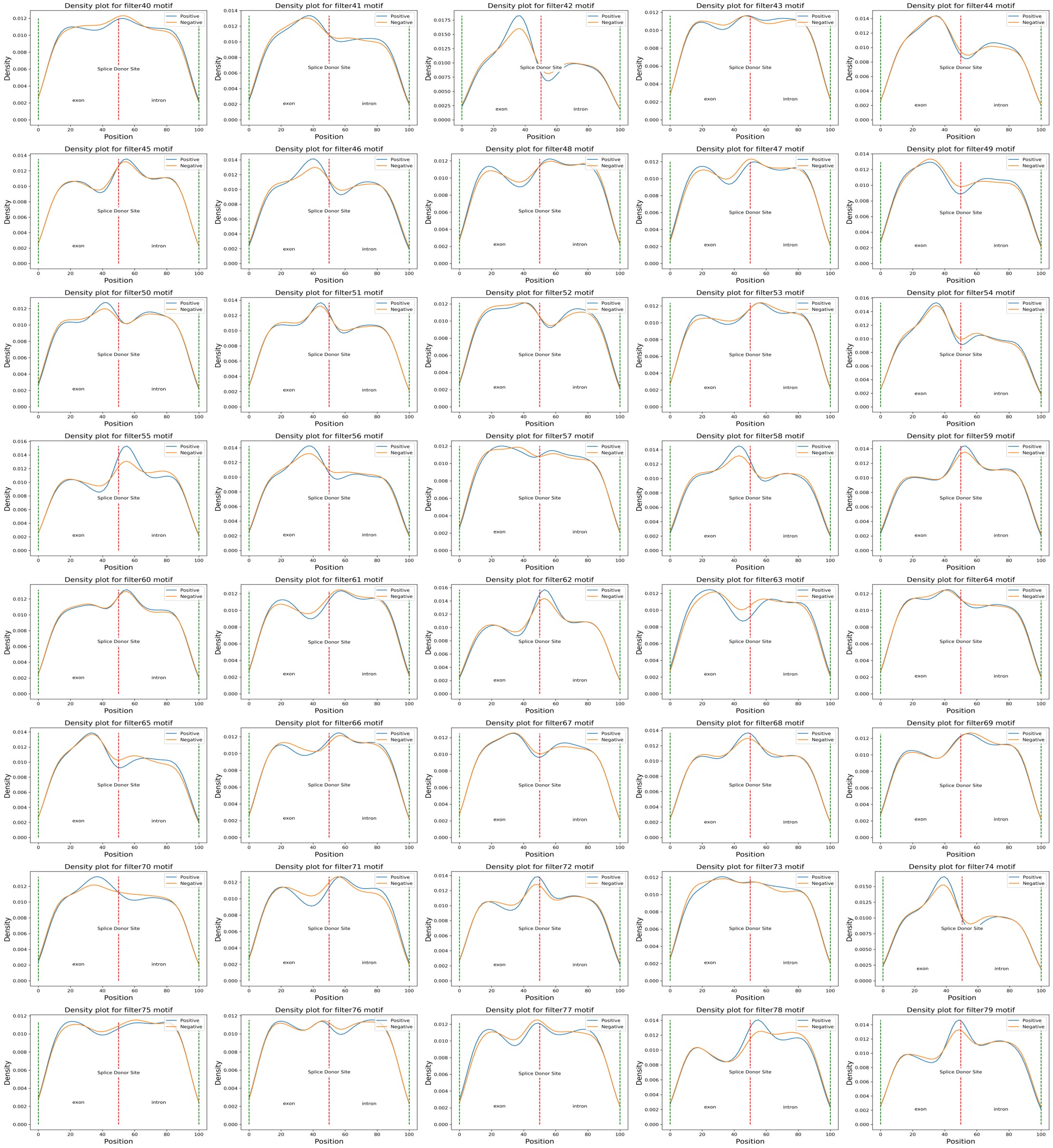


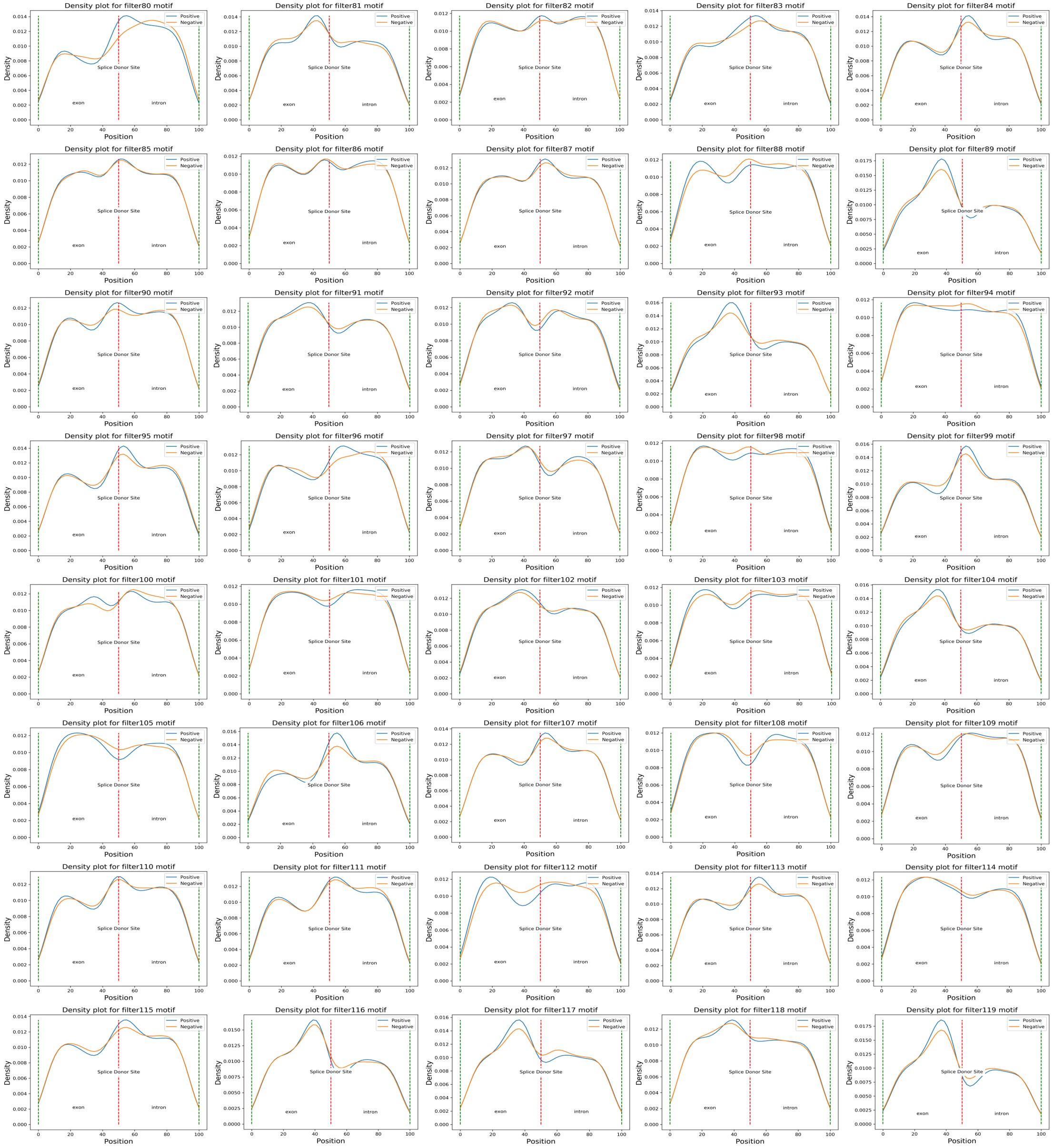


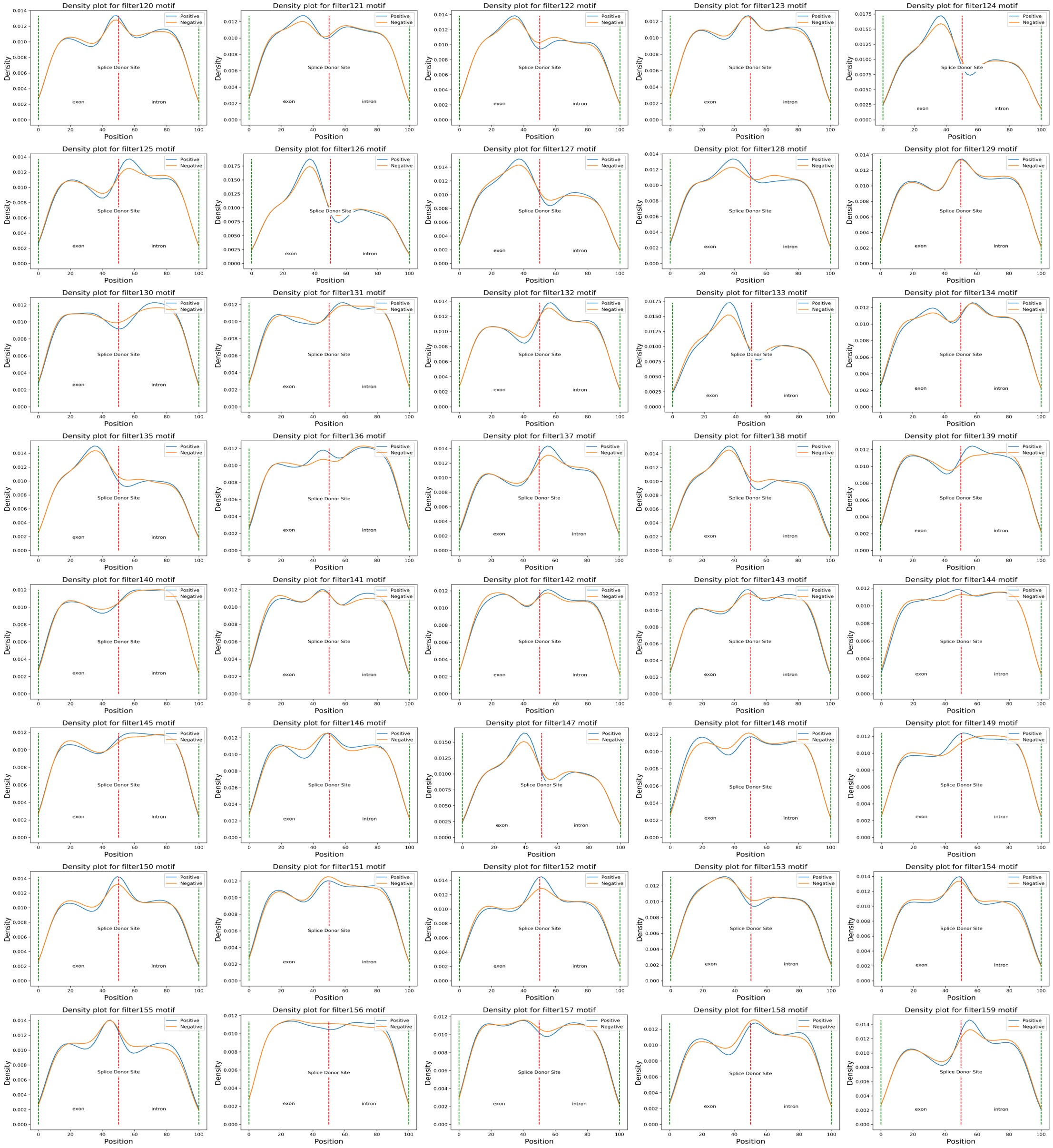


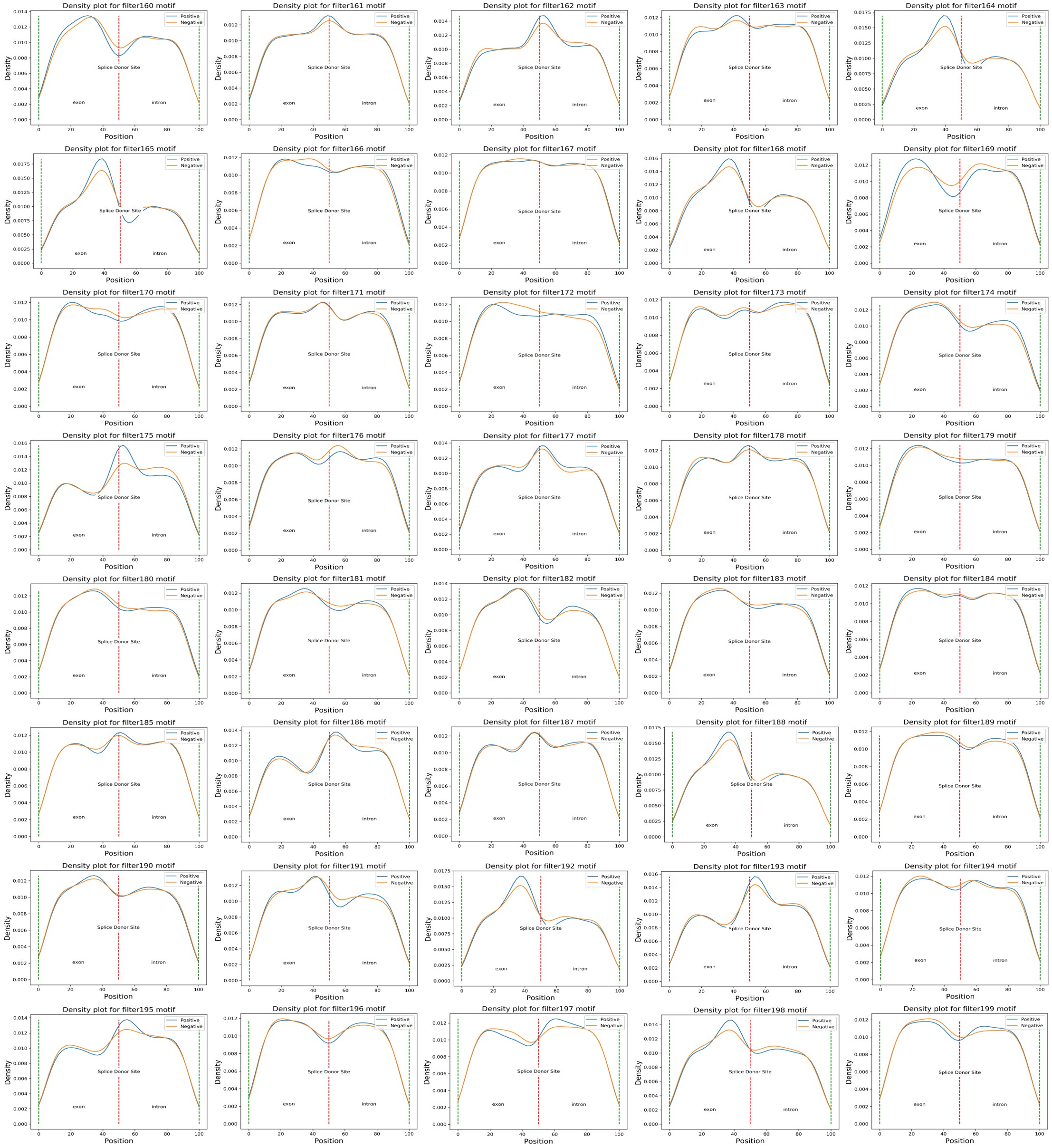


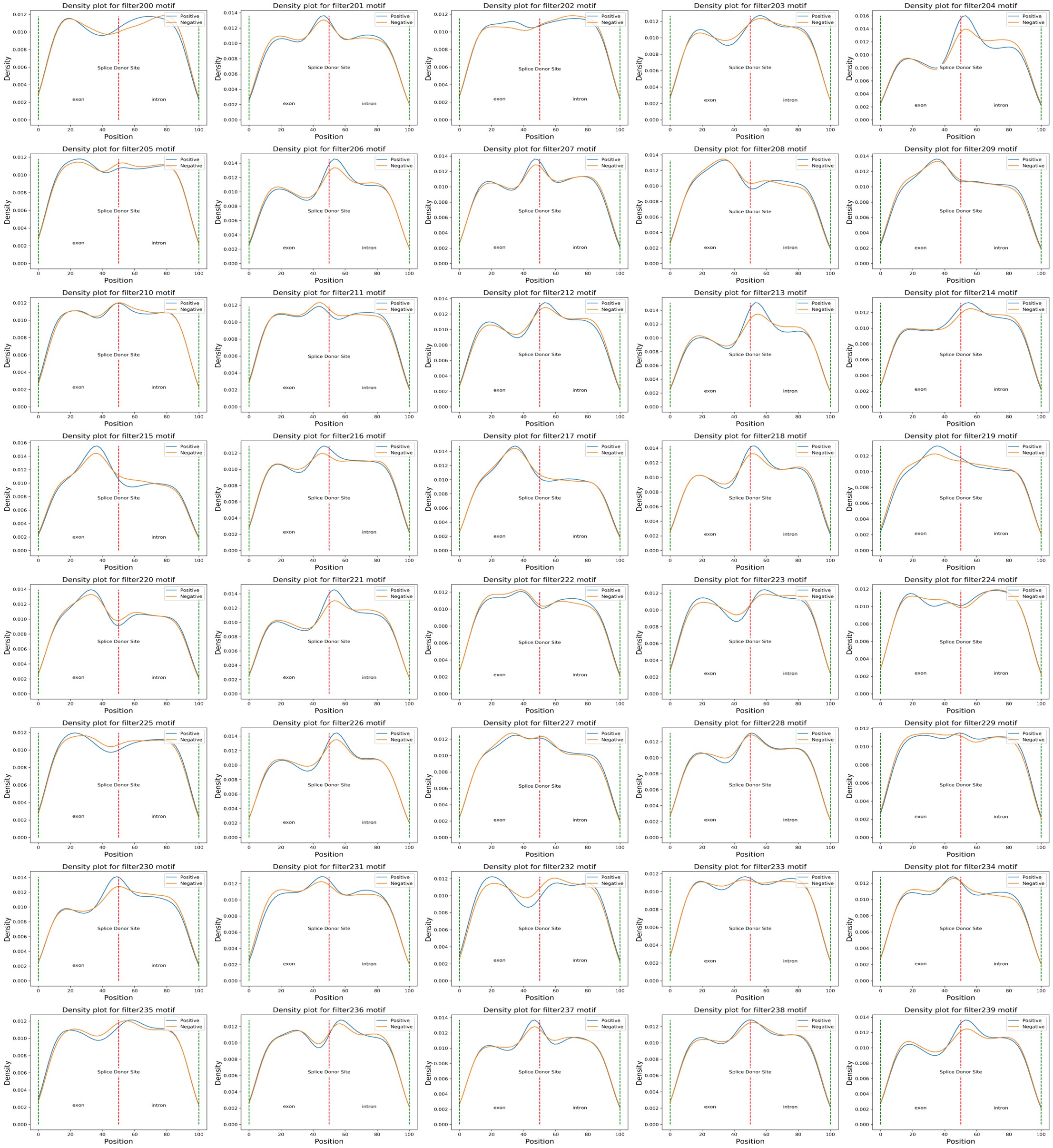


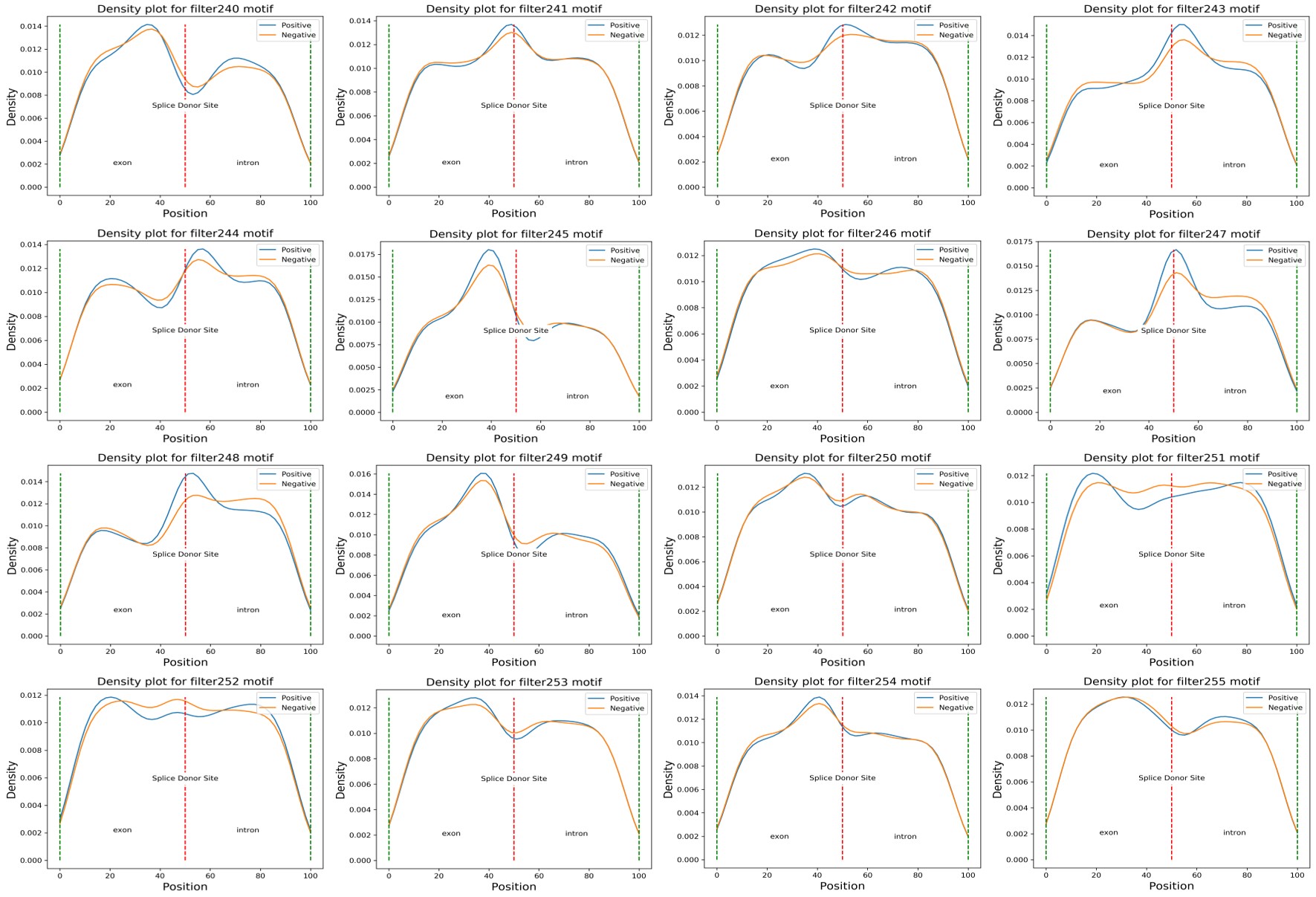


**Figure S5.** Distributions of mouse motifs found by circCNN in the positive and negative mouse circRNAs input2 (SD input). Here, blue line represents positive samples, orange line represents negative samples, red line represents splice donor site, and its left and right are exon and intron, respectively.


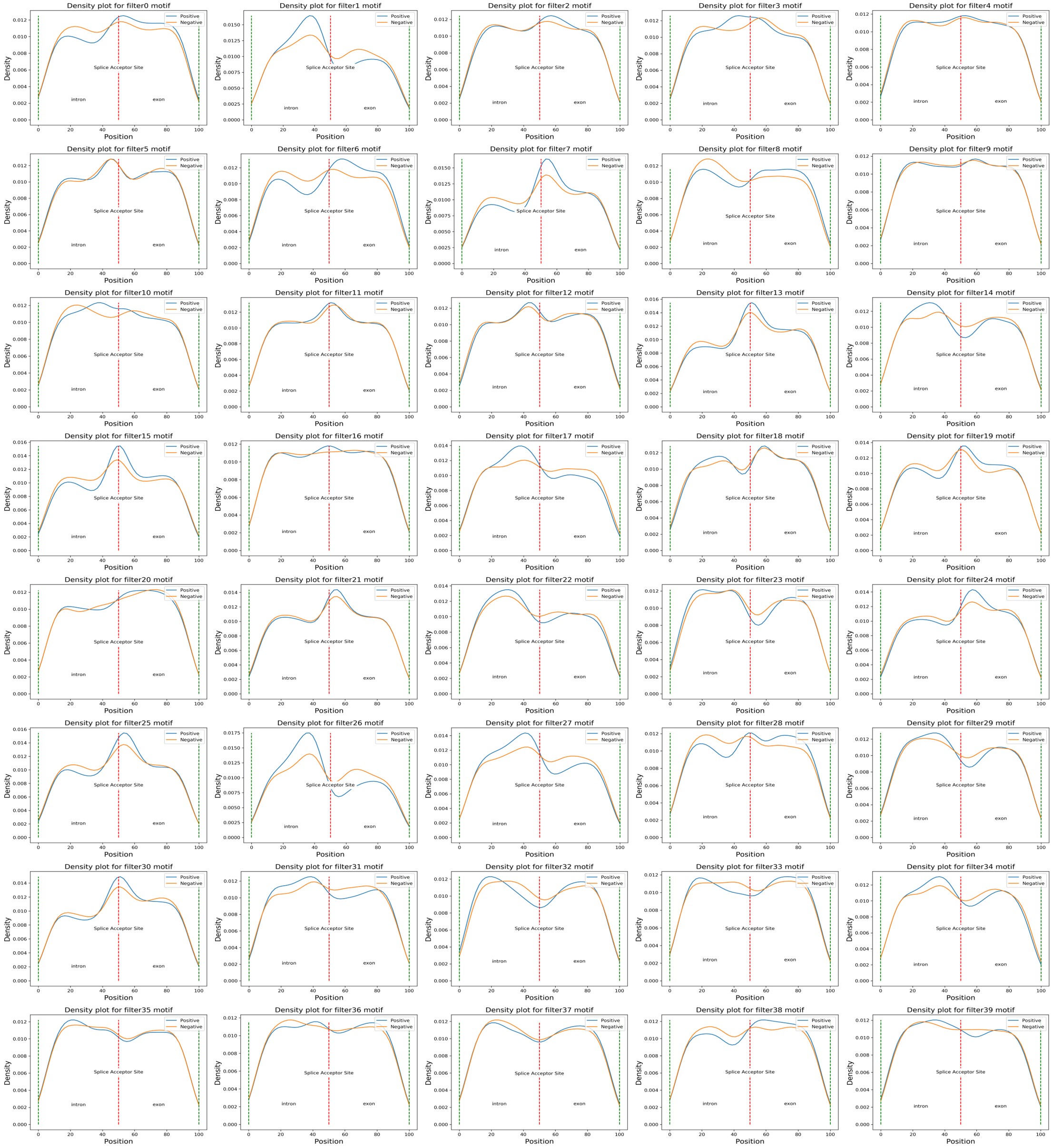


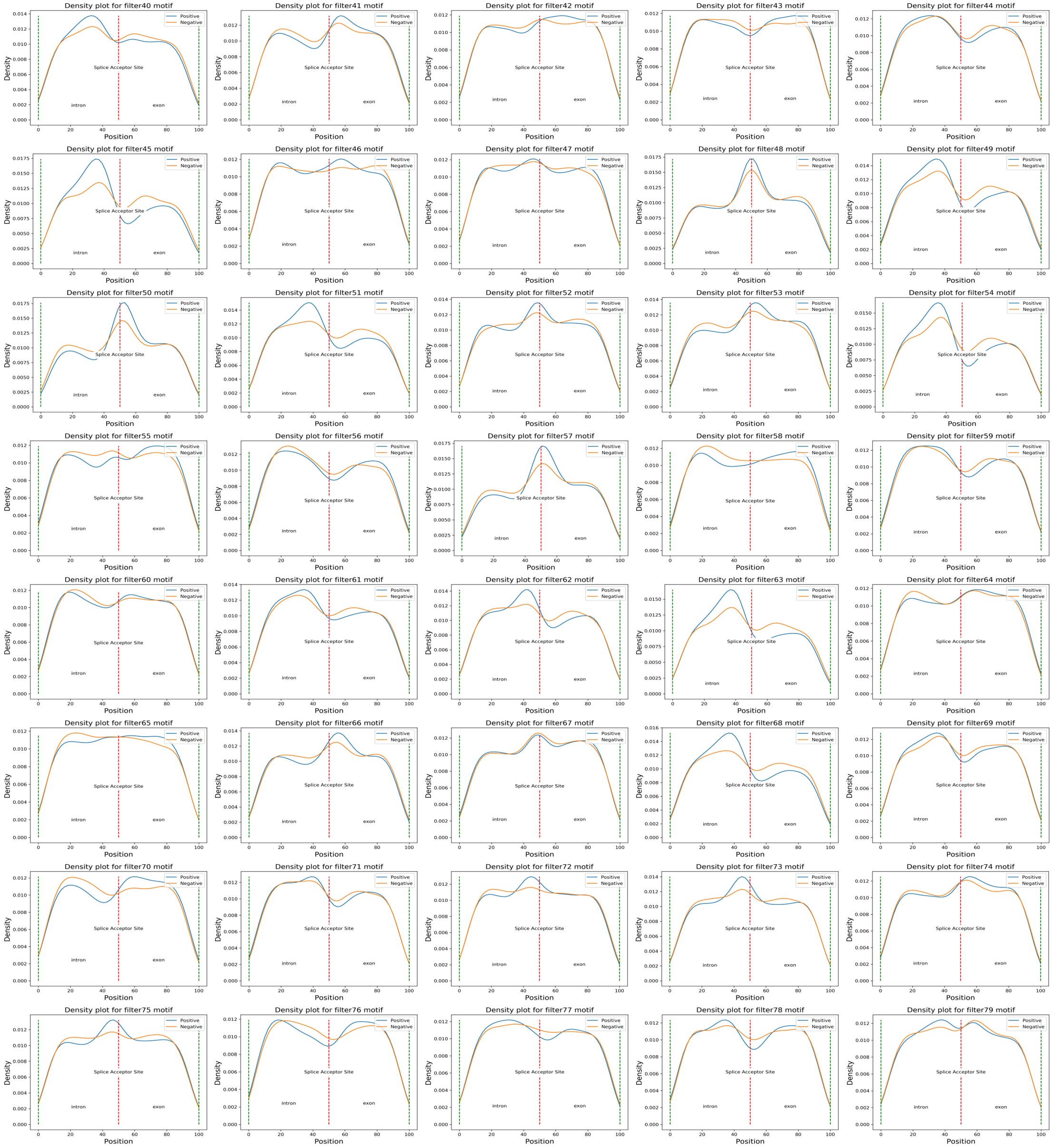


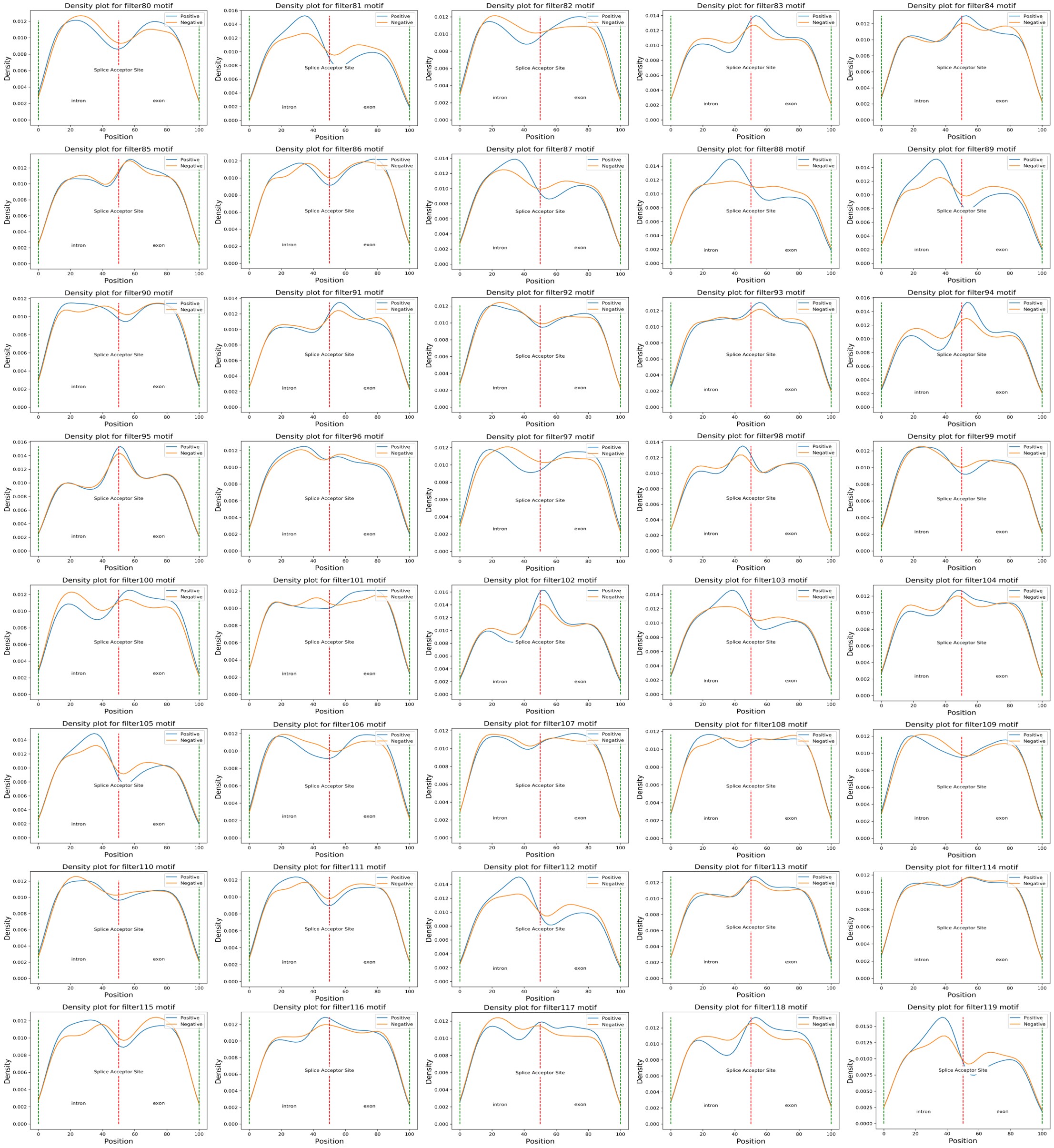


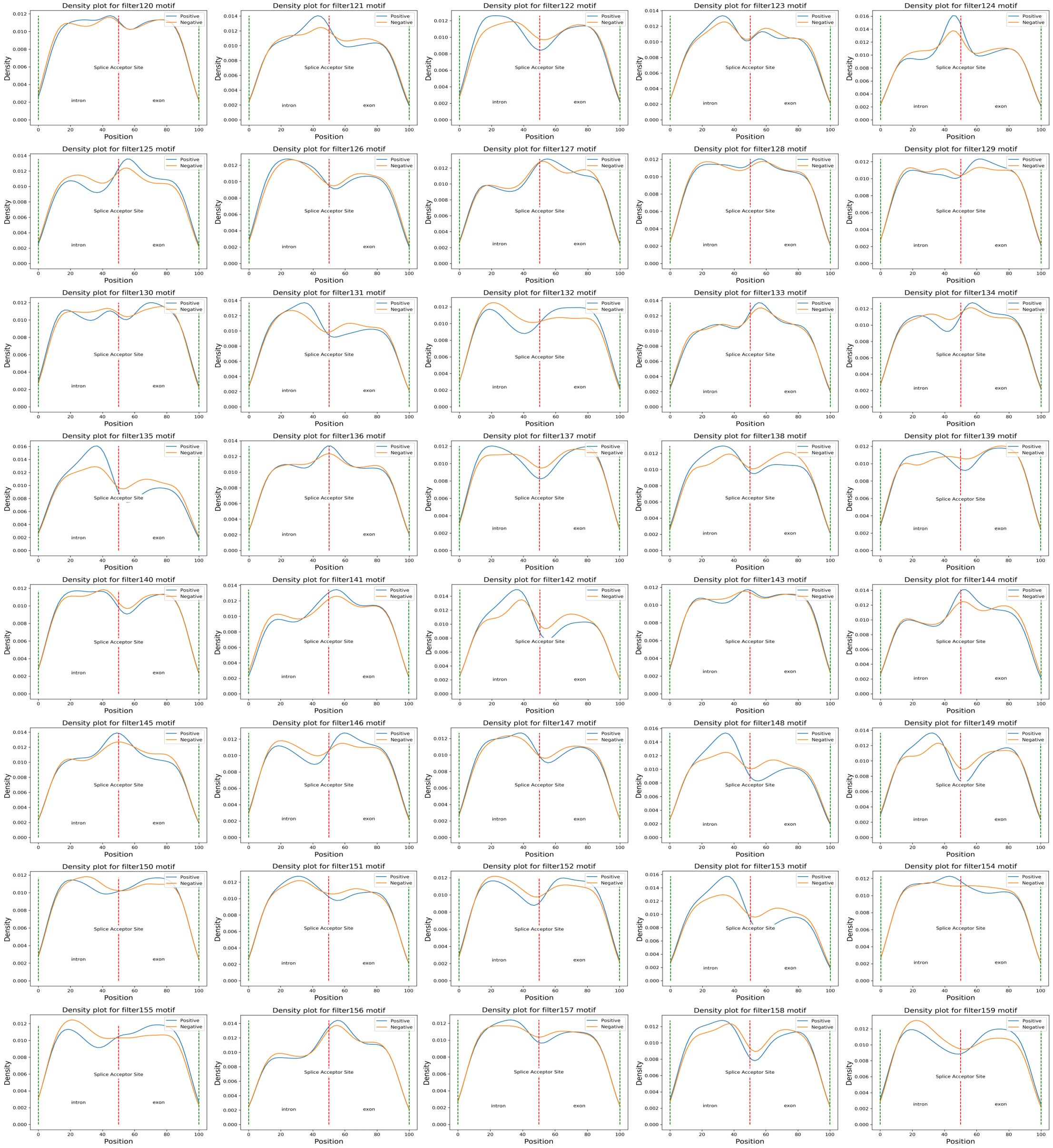


**
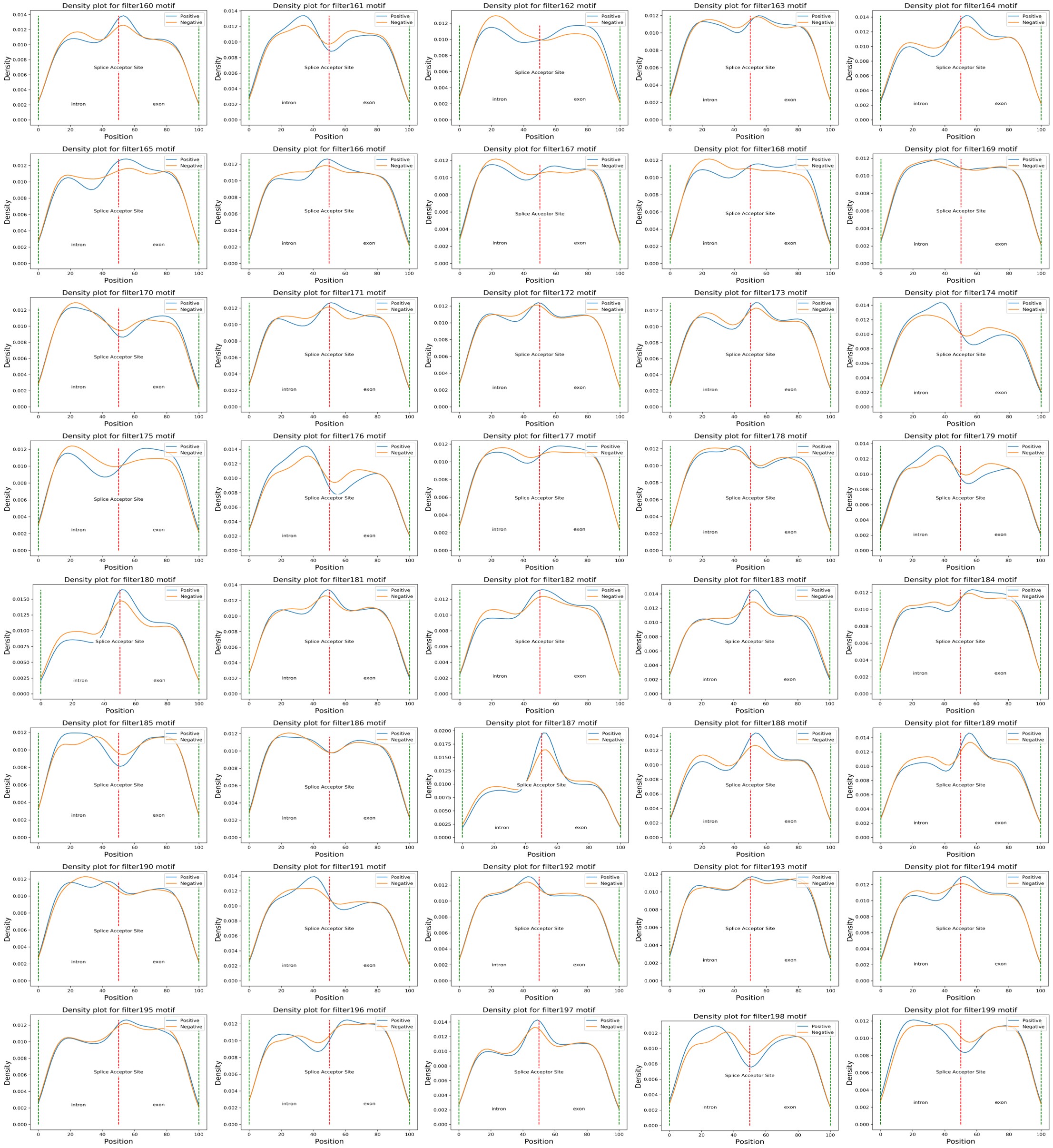
**

**
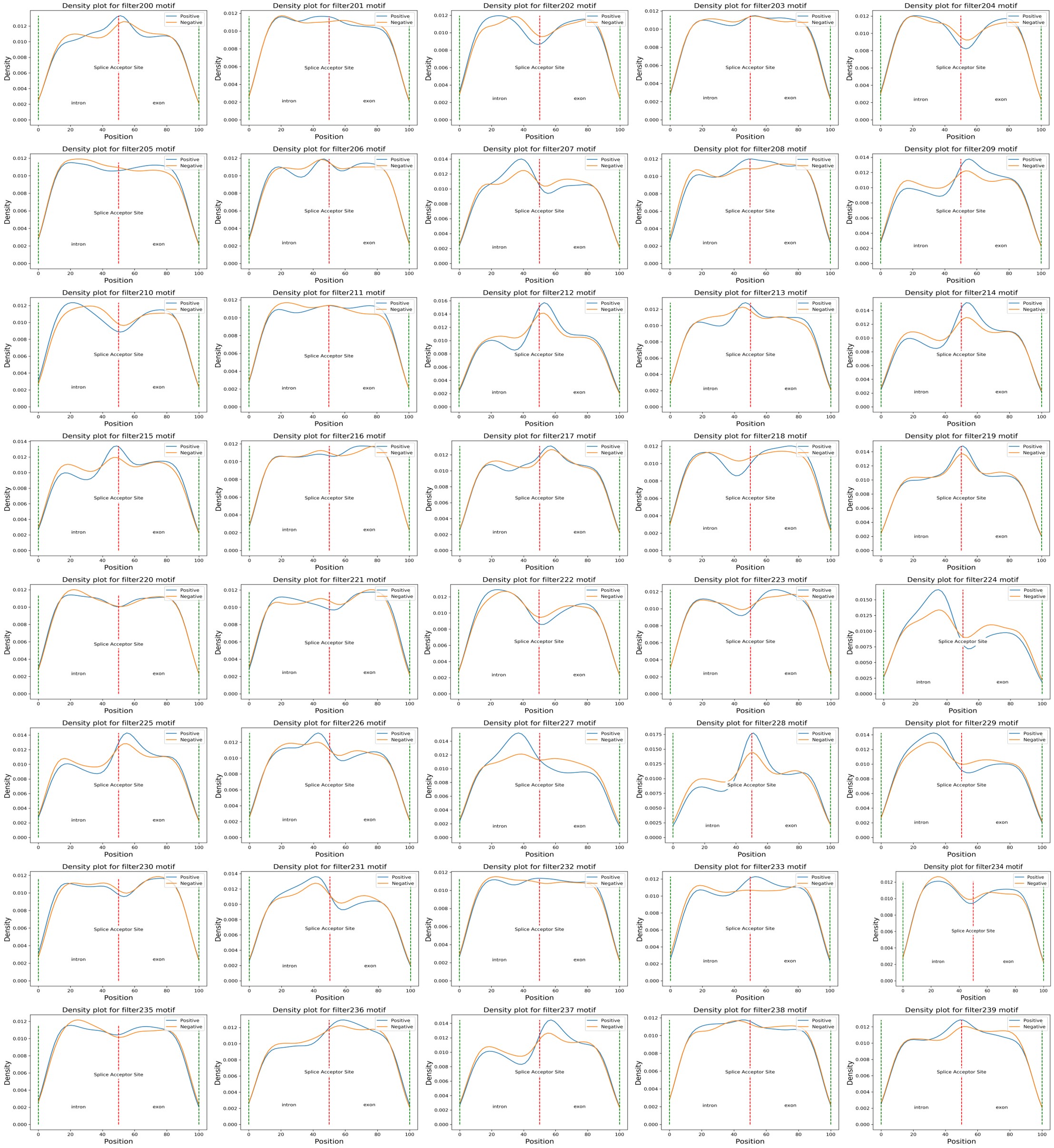
**

**
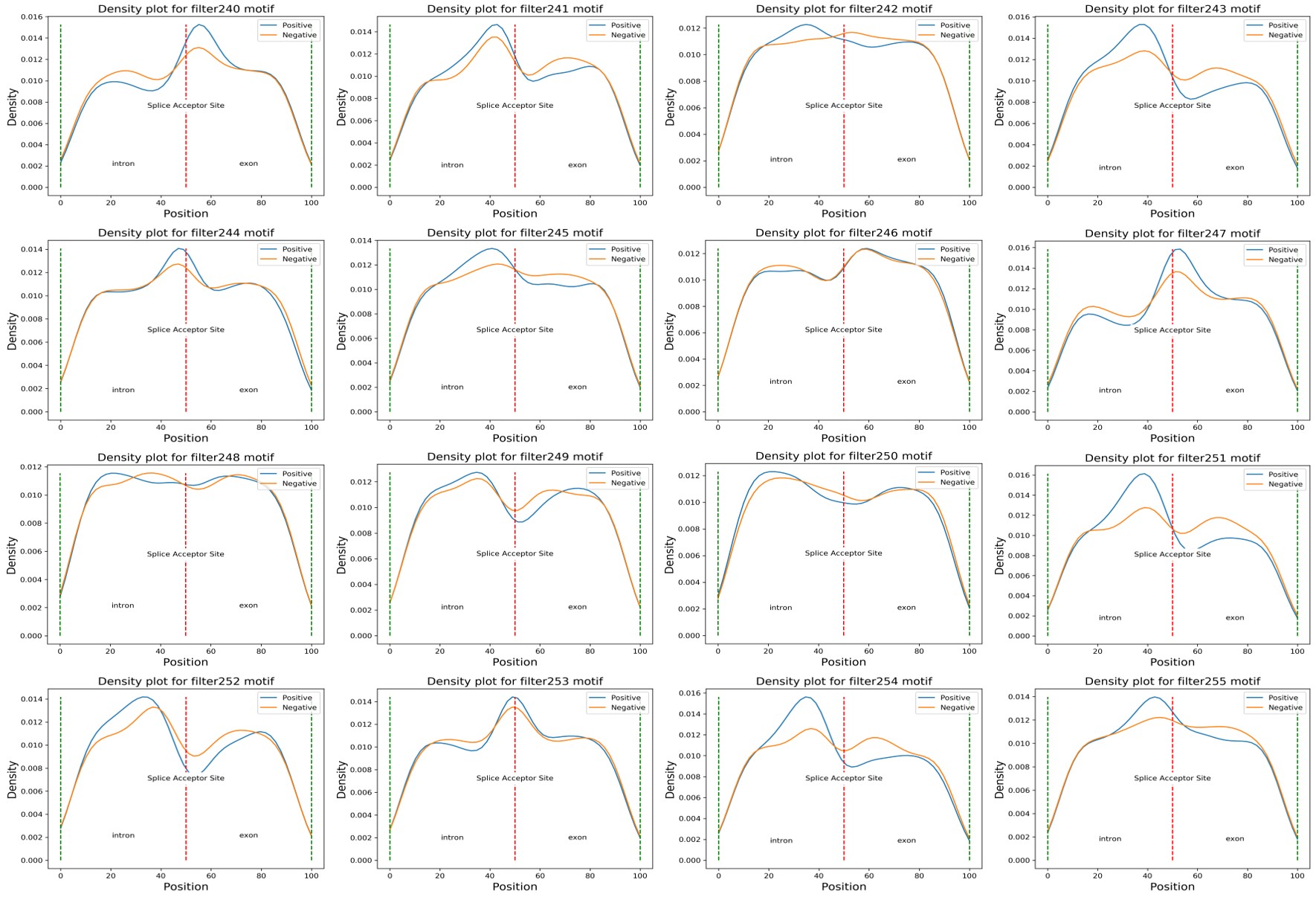
**

**Figure S6.** Distributions of fruit fly motifs found by circCNN in the positive and negative fruit fly circRNAs input1 (SA input). Here, blue line represents positive samples, orange line represents negative samples, red line represents splice acceptor site, and its left and right are intron and exon, respectively.


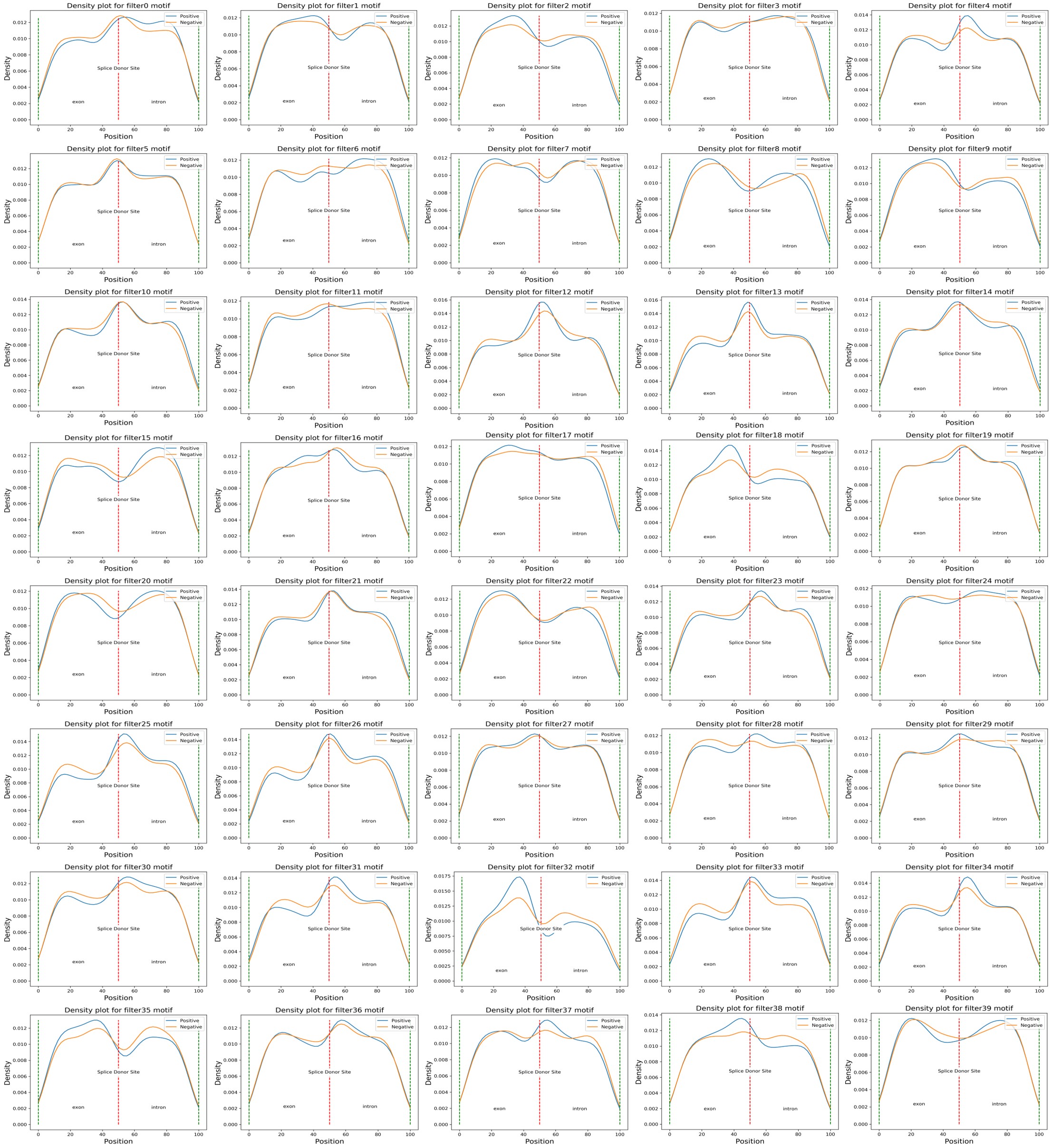


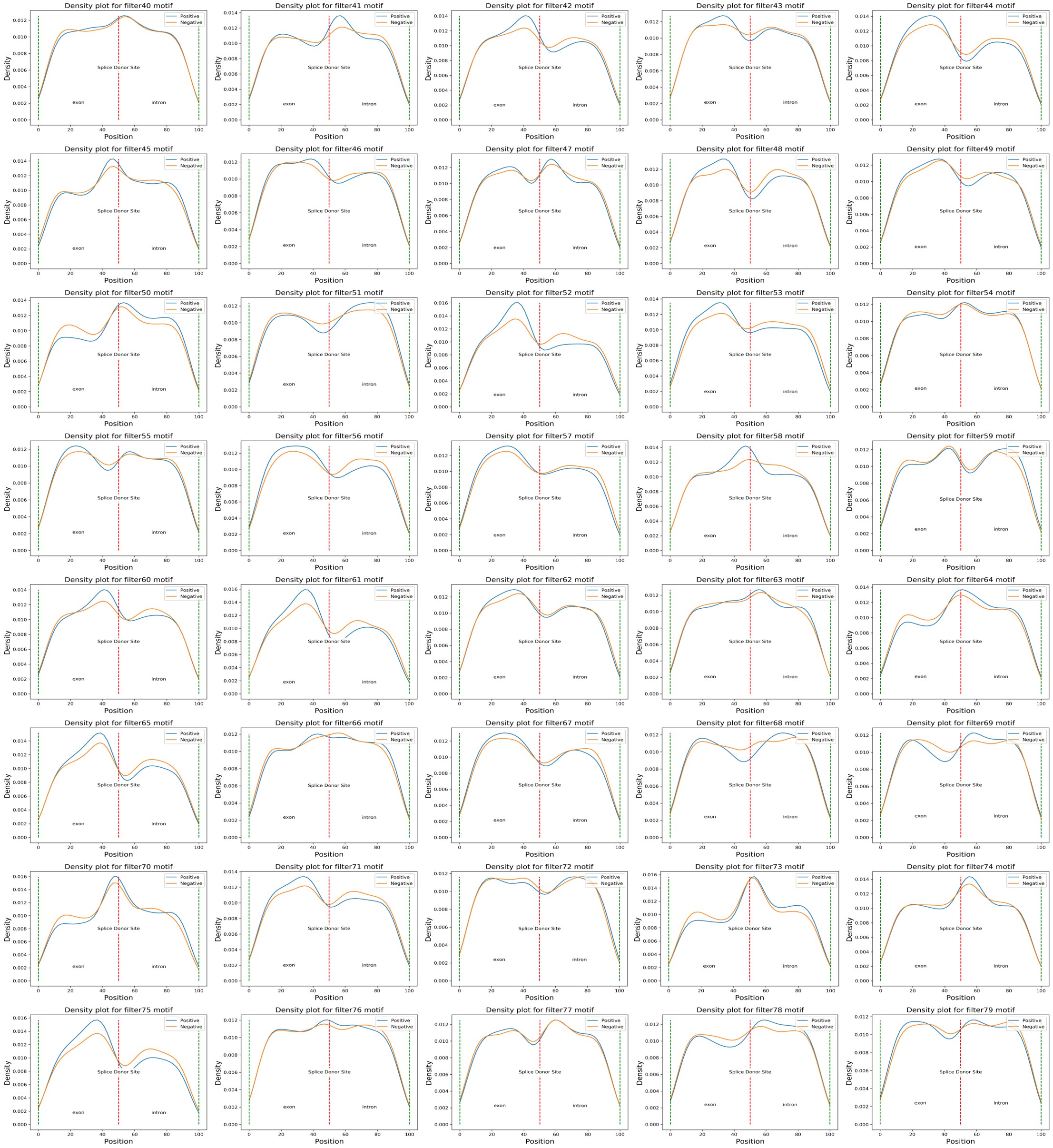


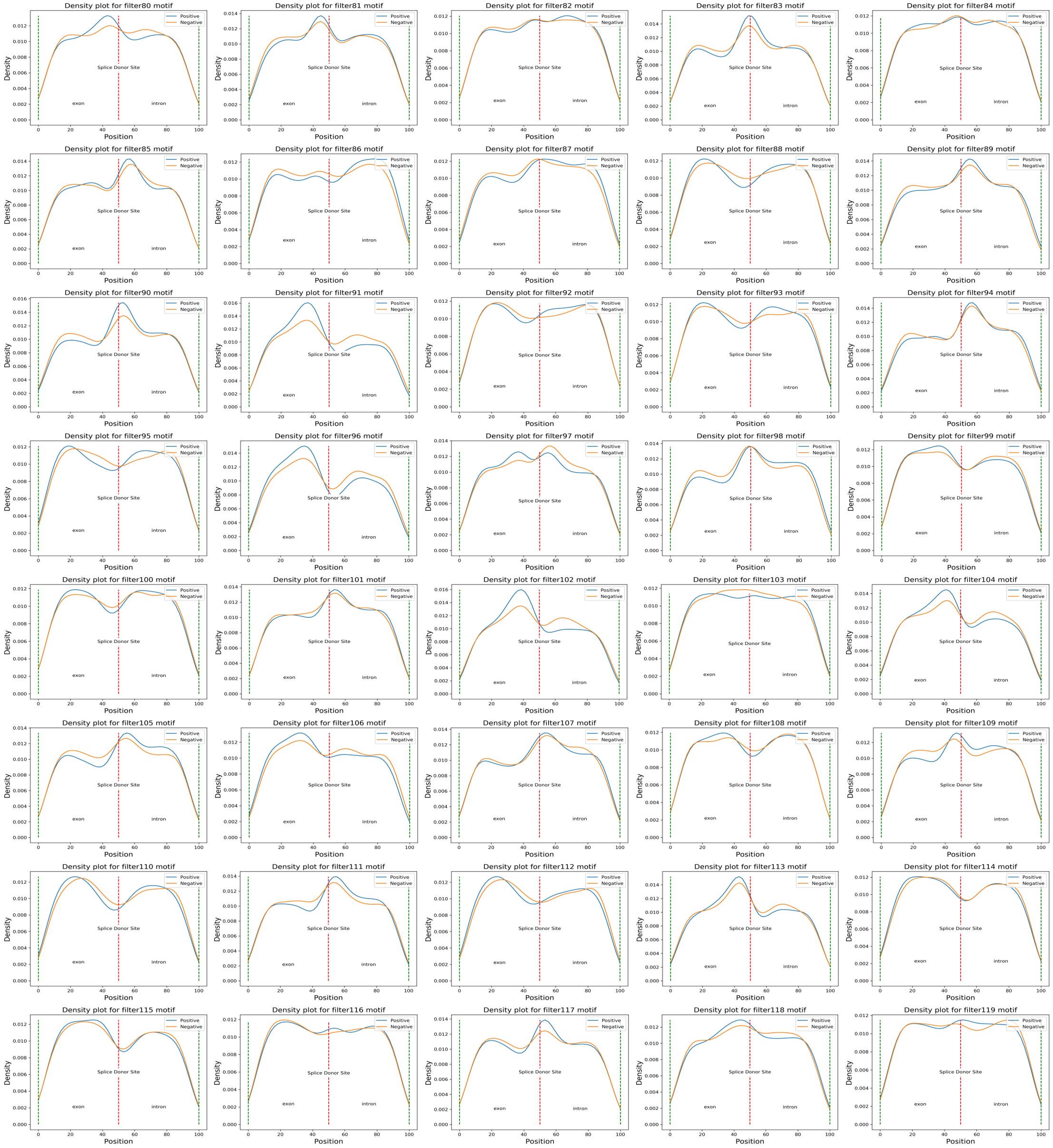


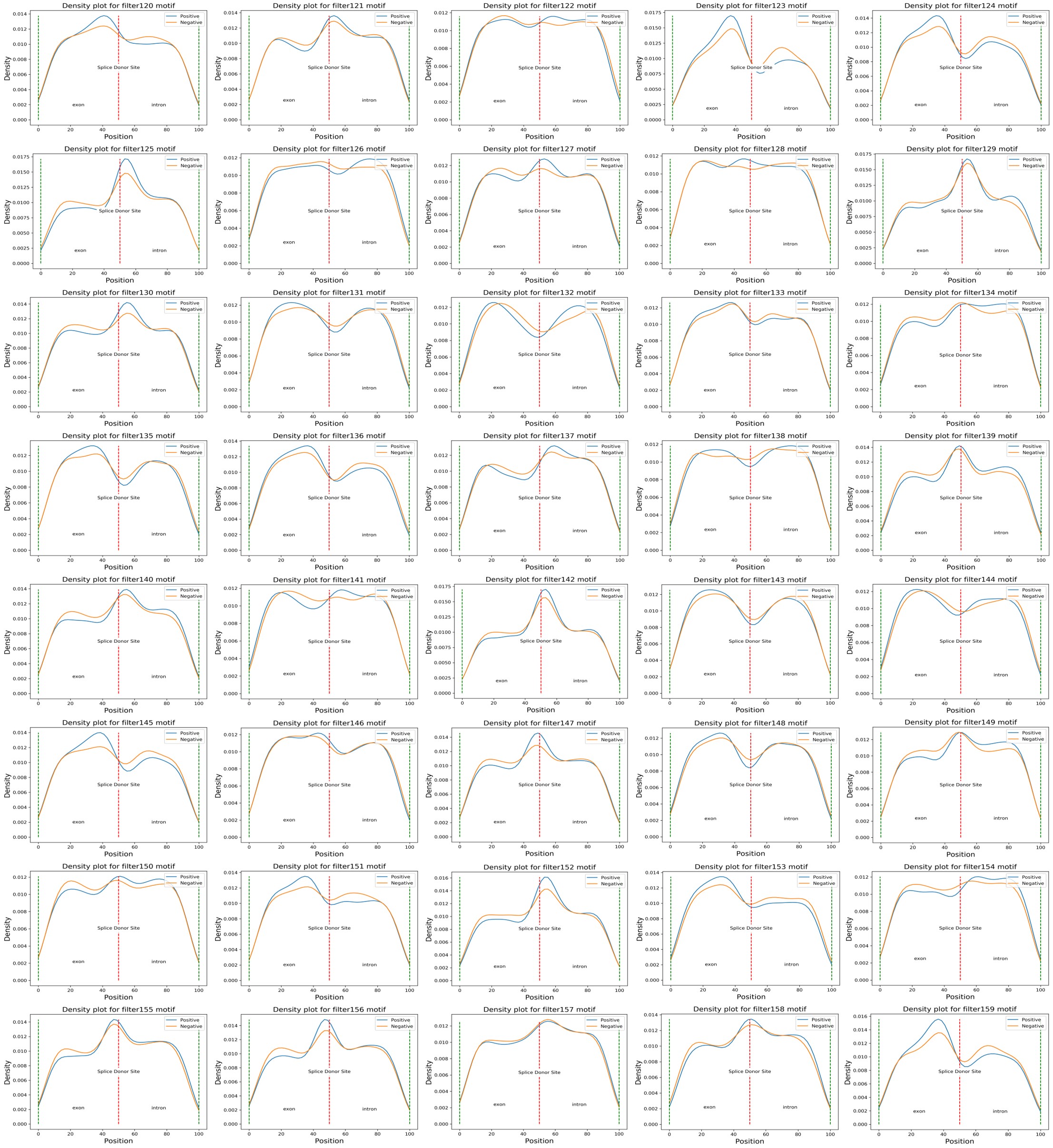


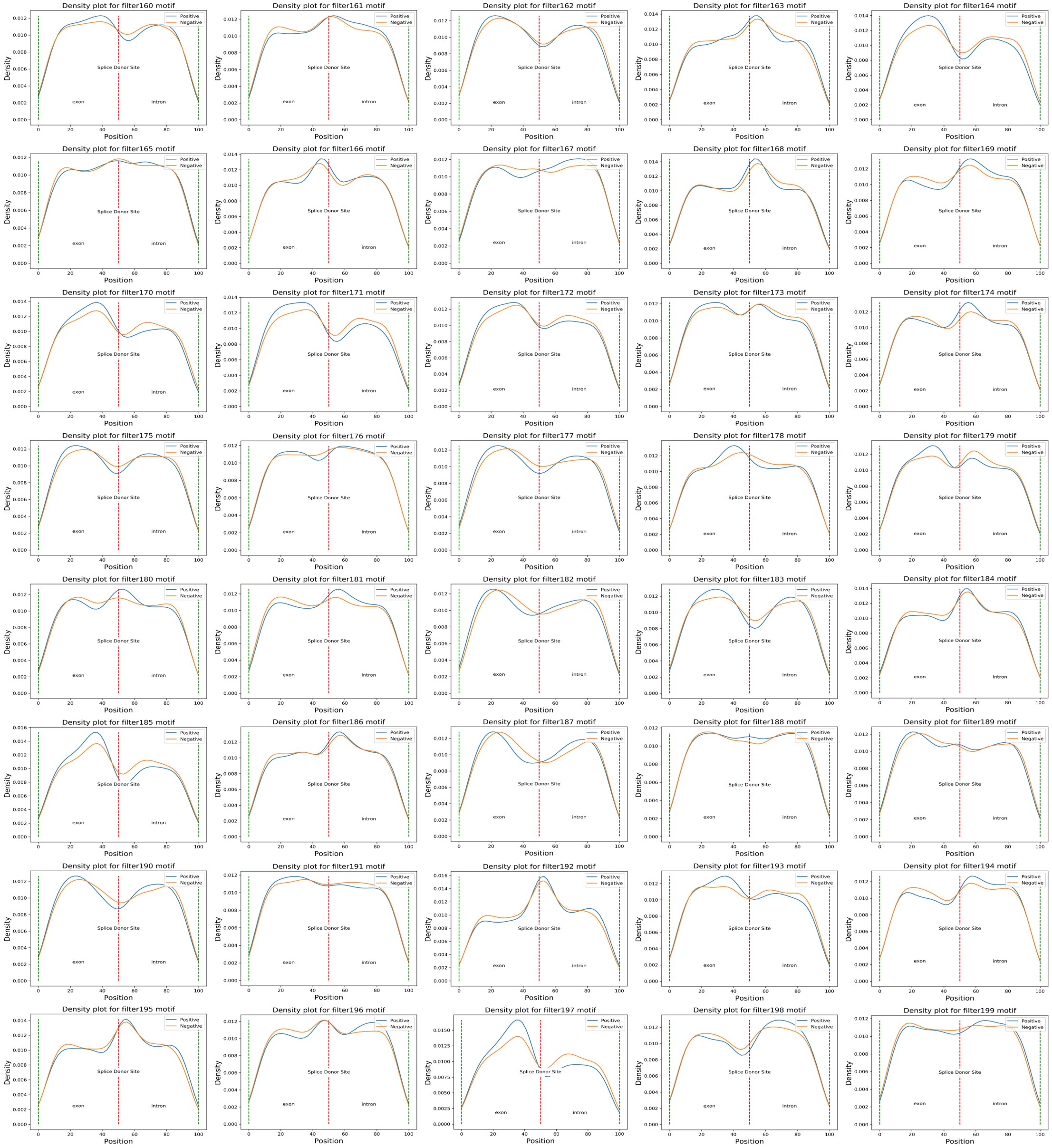


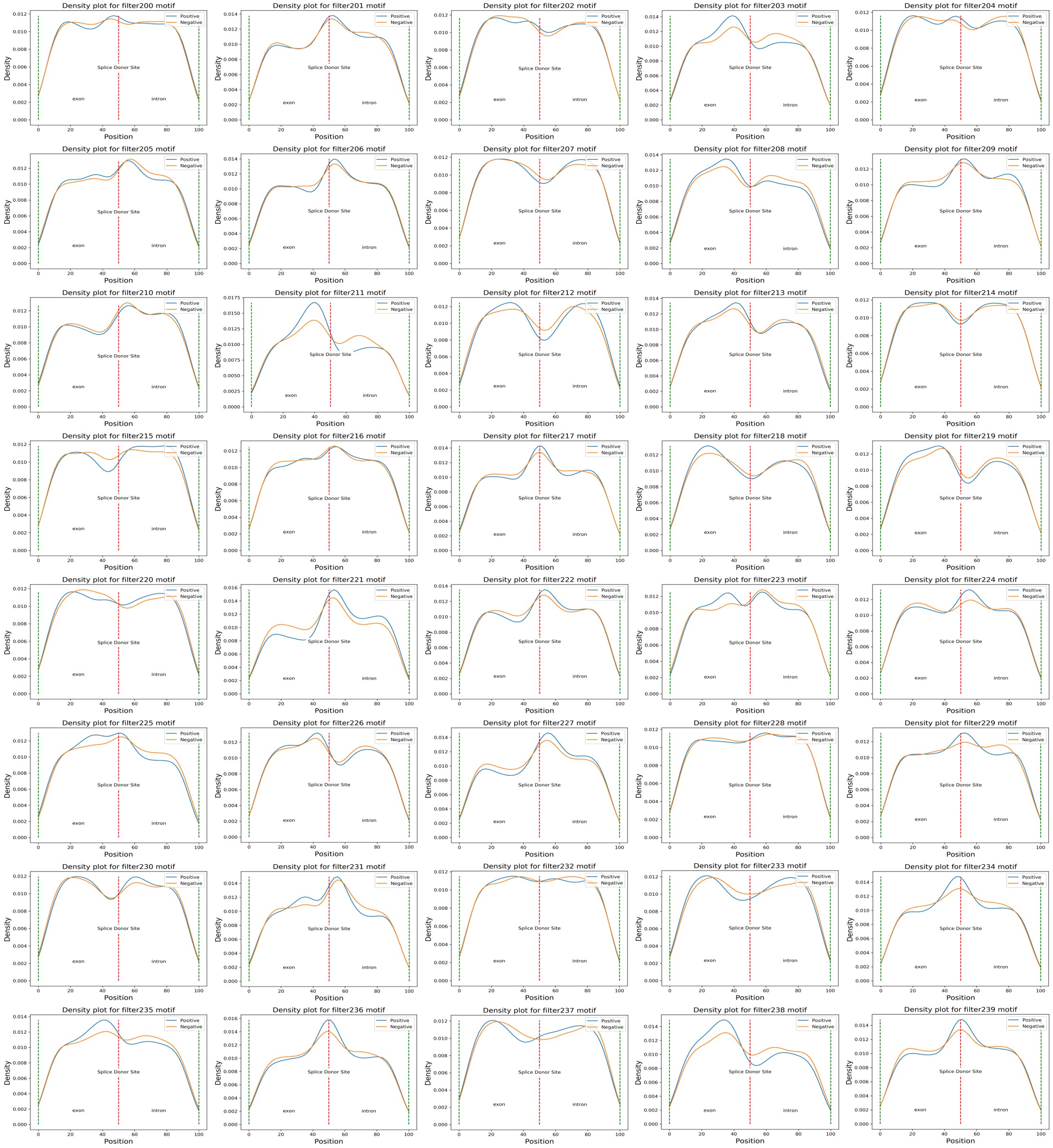


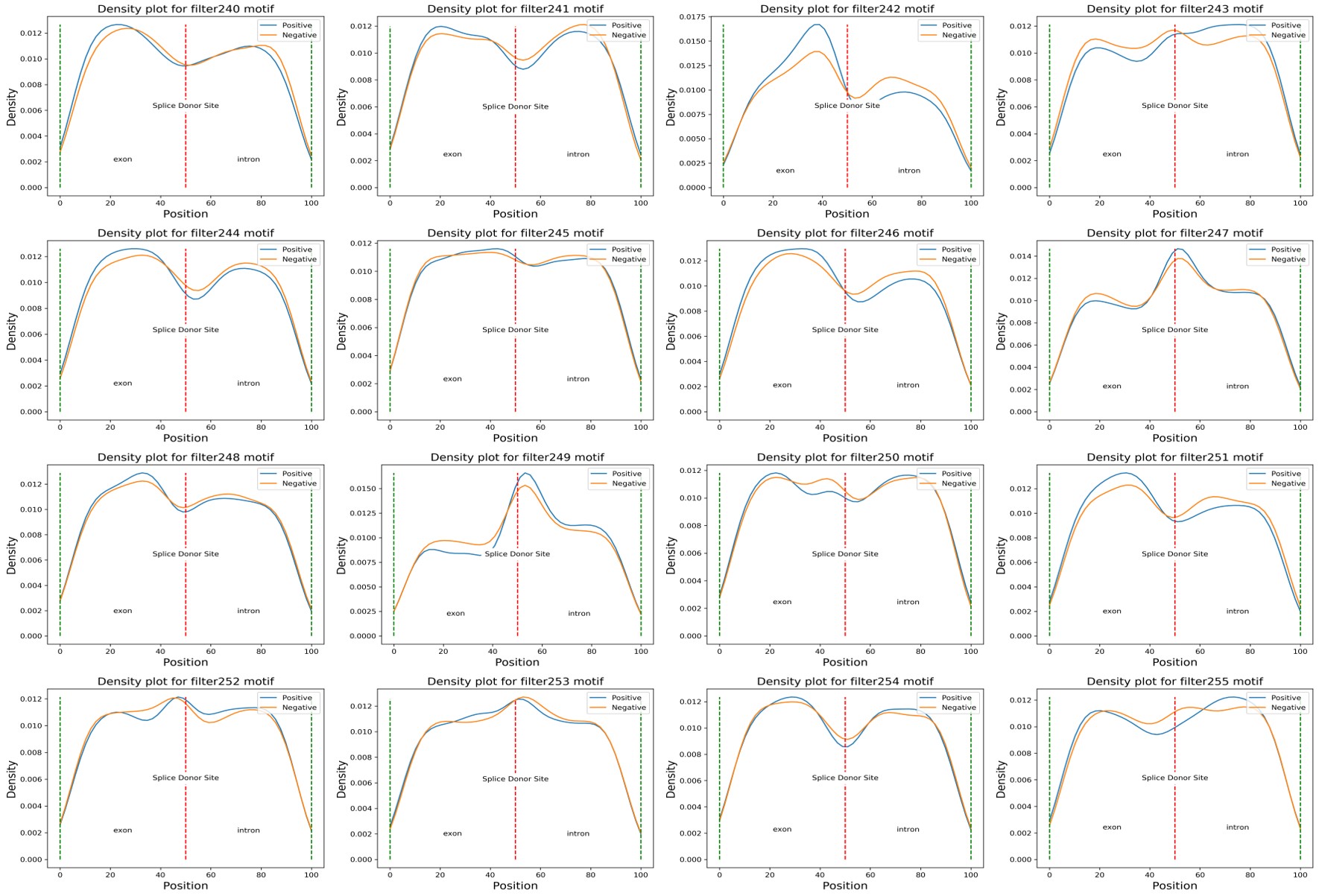


**Figure S7.** Distributions of fruit fly motifs found by circCNN in the positive and negative fruit fly circRNAs input2 (SD input). Here, blue line represents positive samples, orange line represents negative samples, red line represents splice acceptor site, and its left and right are exon and intron, respectively.
